# Structure-Based Design of CBP/EP300 Degraders: When Cooperativity Overcomes Affinity

**DOI:** 10.1021/jacsau.4c00292

**Published:** 2024-08-08

**Authors:** Iván Cheng-Sánchez, Katherine Gosselé, Leonardo Palaferri, Eleen Laul, Gionata Riccabella, Rajiv K. Bedi, Yaozong Li, Anna Müller, Ivan Corbeski, Amedeo Caflisch, Cristina Nevado

**Affiliations:** † Department of Chemistry, University of Zurich, Winterthurerstrasse 190, Zurich CH-8057, Switzerland; ‡ Department of Biochemistry, University of Zurich, Winterthurerstrasse 190, Zurich CH-8057, Switzerland

**Keywords:** PROTAC, fragment docking, cooperativity, structure-based design, chameleon effect, CBP, EP300, bromodomain

## Abstract

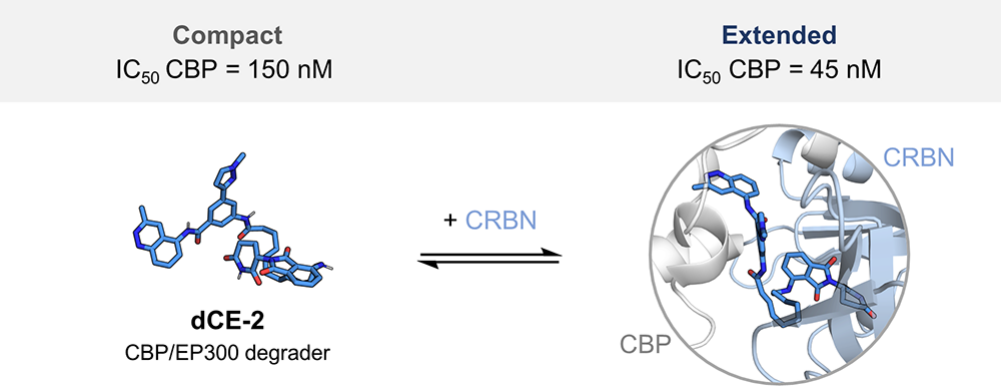

We present the development of **dCE-2**, a structurally novel PROTAC targeting the CREB-binding protein (CBP) and E1A-associated protein (EP300)—two homologous multidomain enzymes crucial for enhancer-mediated transcription. The design of **dCE-2** was based on the crystal structure of an in-house bromodomain (BRD) inhibitor featuring a 3-methyl-cinnoline acetyl-lysine mimic acetyl-lysine mimic discovered by high-throughput fragment docking. Our study shows that, despite its modest binding affinity to CBP/EP300-BRD, **dCE-2**’s remarkable protein degradation activity stems from its good cooperativity, which we demonstrate by the characterization of its ternary complex formation both *in vitro* and *in cellulo*. Molecular dynamics simulations indicate that in aqueous solvents, this active degrader populates both folded and extended conformations, which are likely to promote cell permeability and ternary complex formation, respectively.

## Introduction

Proteolysis targeting chimeras (PROTACs) are an emerging class of small molecules that induce targeted protein degradation by hijacking the cellular proteolysis machinery. Structurally, PROTACs are heterobifunctional molecules that consist of a ligand for the target protein of interest (POI) connected via a linker to a ligand for an E3 ubiquitin ligase. PROTACs initiate a degradation process by establishing a ternary complex involving the POI and the E3 ligase, which end up in close proximity and result in the polyubiquitination and subsequent proteasomal degradation of the POI.^1−4^ Unlike classical small molecule drugs that rely on an occupancy-driven mechanism, PROTACs achieve a complete loss of function of the target protein following a brief binding event. In addition, PROTACs can operate in a catalytic fashion and enhance specificity for close homologues through additional protein–protein interactions (PPIs) between the POI and the E3 ligase.^5−7^

Despite their advantages, PROTACs are significantly larger than the POI ligands from which they derive and generally suffer from poor pharmacokinetic profiles and low cell permeability.^8−10^ To address these problems, recent efforts have been devoted to understanding the physicochemical properties and structure–property relationships of PROTACs.^11−17^ Besides classical parameters, the ability to quickly form a ternary complex stabilized by protein–protein interactions (PPIs) plays a crucial role in PROTAC-mediated degradation which, together with their catalytic mechanism, can compensate for some of the above-mentioned issues.^8^ The different binding affinities of PROTACs to each target protein in the presence of the other is referred to as cooperativity (α) and corresponds to the ratio between the degrader’s binary and ternary affinity. Individually measuring binary ligand affinity during an early PROTAC screening can generate valuable information on structure–activity relationships (SAR) but does not account for the influence of PPIs on ternary complex stability. Therefore, rational analysis to understand PPIs is essential in PROTAC development, but it generally requires obtaining ternary complex crystal or cryo-EM structures, which is far from being a routine task.^18−23^ In consequence, PROTAC development relies heavily on large empirical data sets of synthesized compounds so that methods to better understand the correlation between the physicochemical properties of PROTACs and their target degradation ability are in high demand.

Herein, we present the structure-based development of a novel degrader, **dCE-2**, targeting CREB-binding protein (CBP) and E1A-associated protein (EP300). CBP and EP300 are two transcriptional cofactors that regulate gene expression^24−26^ through numerous PPIs^27^ and by acetylating histone and nonhistone proteins.^28,29^ CBP/EP300 are implicated in a wide range of diseases, such as cancer, inflammation, and developmental disorders.^30−34^ Several CBP/EP300 PROTACs based on different bromodomain (BRD)^35−38^ and histone acetyl transferase (HAT)^39,40^ ligands have been reported by our^41^ and other groups.^42−50^ In this work, we used high-throughput docking^51^ to identify an unprecedented 3-methylcinnoline fragment as an acetyl-lysine mimic. Subsequent structure-based optimization of this fragment led to the discovery of a BRD inhibitor,^52^ which was further developed into PROTAC **dCE-2**. In-depth characterization of our degrader showed that, despite its modest binding affinity to CBP/EP300-BRD, it induces robust ternary complex formation with good cooperativity, both *in vitro* and *in cellulo*. Explicit water molecular dynamics (MD) simulations suggest that **dCE-2** is able to populate both folded and extended conformations, which are likely to promote cell permeability and ternary complex formation, respectively, thus signalling the need for a broader set of parameters to streamline the design of efficient PROTACs.

## High-Throughput Fragment Docking and Warhead Selection

**Figure 1:**
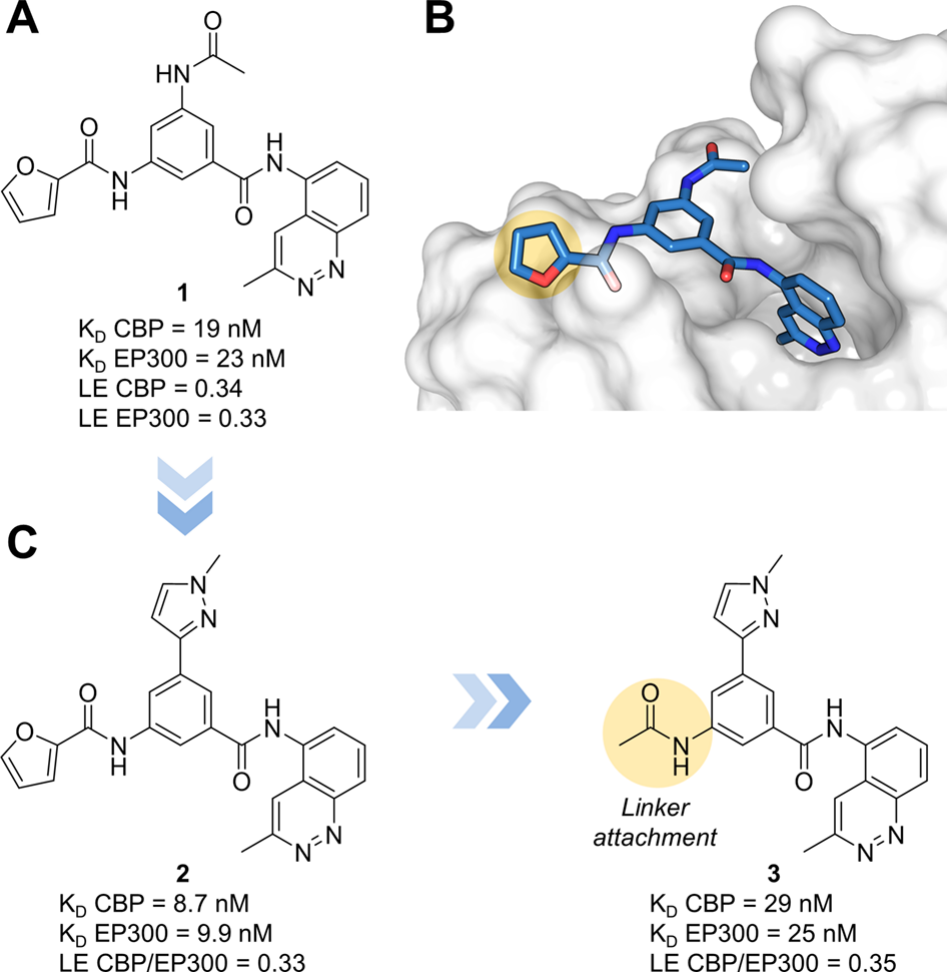
CBP/EP300-BRD ligands selected for structure-based optimization and PROTAC development. (A) Chemical structure, CBP/EP300 *K*_D_ values, and ligand efficiency (LE) of **1**. (B) Crystal structure of **1** in complex with CBP-BRD (PDB: 6SQM). (C) Chemical structure, CBP/EP300 *K*_D_ values, and LE of compounds **2** and **3**. Linker attachment site highlighted in yellow. *K*_D_ values were determined using BROMOscan technology.

At the beginning of this study, we decided to identify a potent and selective fragment hit using docking into the bottom of the acetyl-lysine pocket. The docking program SEED^53^ was employed as it is very efficient (about 2 s per fragment, see the Supporting Information) and has produced hit fragments for a large variety of protein targets of pharmaceutical relevance.^54−60^ The binding energy evaluation in the program SEED is based on the CHARMM force field and an implicit model of the solvent.^53,61−63^ From the *in silico* screening of a library of about 500 small molecules (mainly heteroaromatics with molecular weight (MW) below 300 g/mol),^64^ a 3-methylcinnoline moiety emerged as the fragment with the most favorable SEED-predicted binding energy (−19.9 kcal/mol), favorably comparing to previously reported *N*,*N*-dimethylacetamide (−19.5 kcal/mol)^65^ and acetophenone (−16.3 kcal/mol)^66^ scaffolds. Additionally, the methylcinnoline fragment displayed selectivity for CBP over the N-terminal bromodomain of BRD4 (BRD4(1)), with the predicted binding energy for BRD4(1) being −16.5 kcal/mol. The fragment-growing strategy was inspired by the visual analysis of the overlap of the docked pose of 3-methylcinnoline in the CBP bromodomain and the crystal structure of the complex with a previously reported acetophenone-based ligand also developed in-house (compound 16 of ref (51); PDB code 5NLK) which suggested the replacement of the acetophenone group with the 3-methylcinnoline to generate compound **1** (Figure 1A). Subsequent optimization for *in cellulo* and *in vivo* applications resulted in compound **2**.^52^ To ease PROTAC development and linker attachment, the furane of **2** was replaced by a methyl group leading to compound **3**. Compound **3** showed very good binding affinity (*K*_D_ CBP/EP300 = 29/35 nM) and an even better ligand efficiency (LE CBP/EP300 = 0.35) when compared to **2**. Based on the crystal structure of **1** in complex with CBP/EP300-BRD (PDB: 6SQM) and assuming a similar binding mode, the acetamide of **3** was chosen as a prominent position for future conjugation with the linker moieties.

## Protac Synthesis and Screening

**Figure 2:**
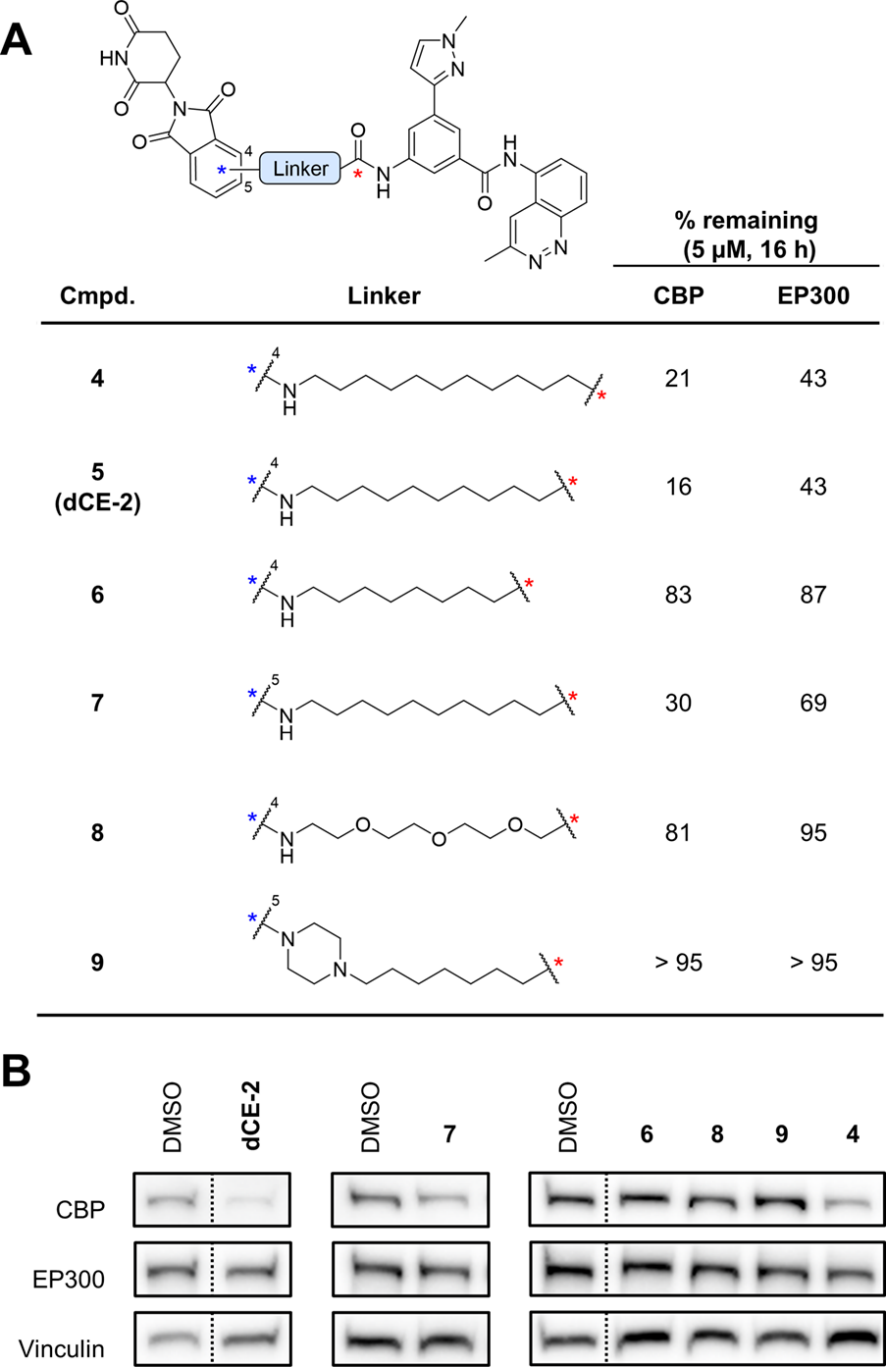
Refined PROTAC screen. (A) Chemical structures of PROTAC molecules and quantification of CBP and EP300 levels by Western blotting following 16 h treatment of LP1 cells with 5 μM compound. Vinculin was used as a loading control for normalization. (B) Representative images of Western blots quantified in (A), all images in Figure S2.

A small library^67^ of potential degraders was prepared by conjugating various linkers at the acetamide vector site and targeting the cereblon (CRBN) E3 ligase using thalidomide as a ligand (Figure S1). To our pleasant surprise, compound **4**—featuring an 11-atom aliphatic linker—showed significant degradation of CBP, and to a lesser extent EP300, in the multiple myeloma LP1 cell line (5 μM compound, 16 h treatment). Next, linkers of different lengths and alternative points for the connection to the thalidomide ligand were explored resulting in compounds **4–9** (Figure 2). This small SAR campaign revealed the key role played by the linker length and the attachment point. Compared to our initial hit **4** (% remaining CBP/EP300 = 21/43), shortening the 11-carbon aliphatic linker by a single atom (**5**, **dCE-2**) slightly improved the degradation of CBP (% remaining = 16). However, further shortening the linker (8 carbon atoms, compound **6**) resulted in an abrupt loss of degradation (% remaining CBP/EP300 = 83/87), likely due to steric clashes between CBP/EP300 and the ligase. As **dCE-2** bears the most favorable linker length, we performed an optimization of the linker composition using this length. Conjugation via the 5′ position to thalidomide (**7**) led to a slight decrease in degradation potency (% remaining CBP/EP300 = 30/69) while further attempts to improve solubility or cellular permeability through PEG (**8**, % remaining CBP/EP300 = 81/95) or piperazine groups (**9**, % remaining CBP/EP300 > 95) significantly reduced degradation. Thus, we selected **dCE-2** for in-depth characterization.

## Biological Characterization of Protac **dCE-2**

**Figure 3:**
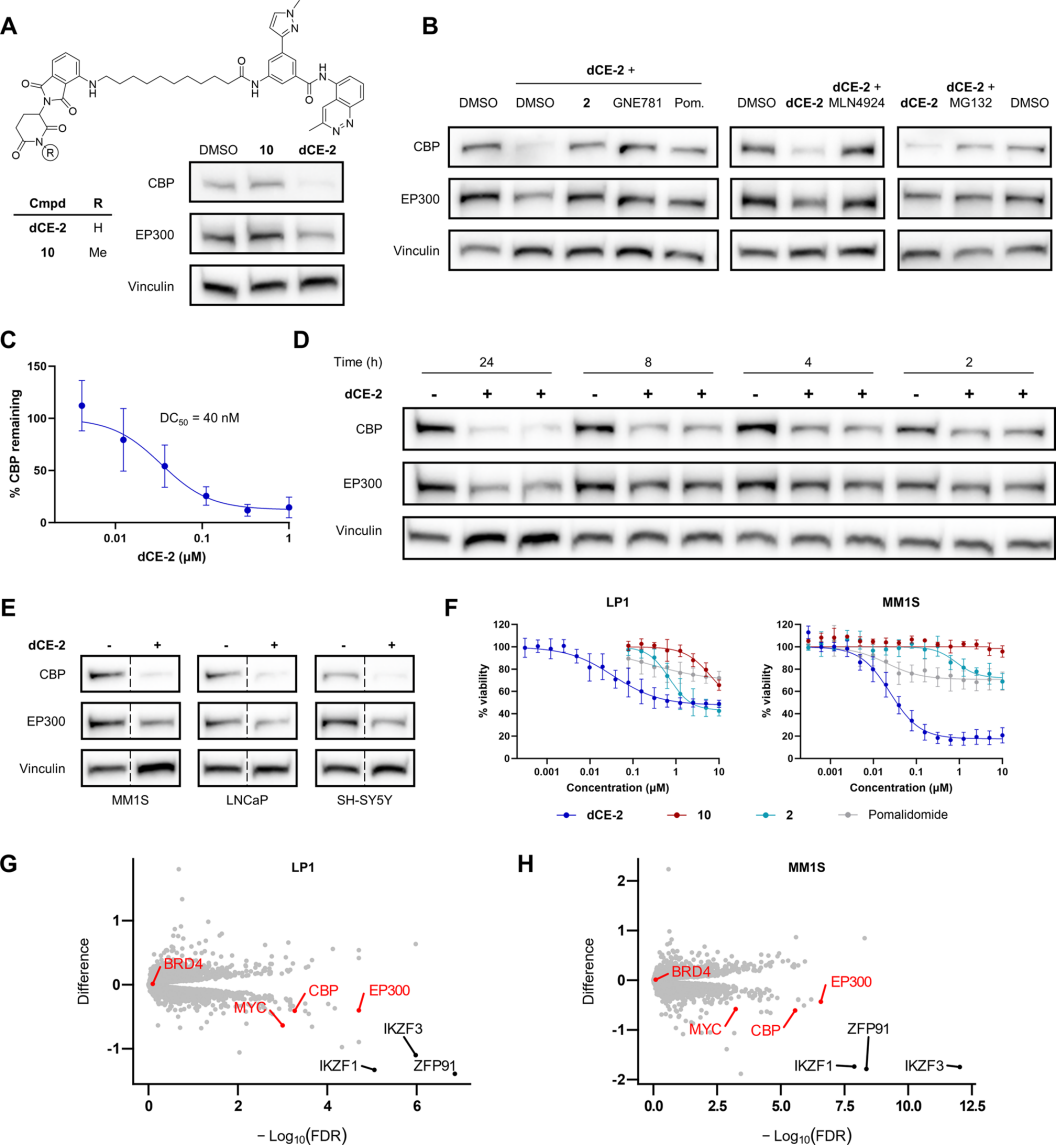
Characterization of **dCE-2**. (A) Chemical structure of **dCE-2** and its negative control **10** and Western blot measurements of CBP and EP300 levels in LP1 cells after 16 h treatment with 1 μM of these compounds. (B) Western blot measurements of CBP and EP300 levels in LP1 cells pretreated for 1 h with 10 μM **2**/10 μM GNE-781/50 μM pomalidomide followed by 16 h 1 μM **dCE-2** (left); pretreated for 2 h with 1 μM MLN4924 followed by 16 h 1 μM **dCE-2** (center); pretreated for 30 min with 10 μM MG132 followed by 6 h 1 μM **dCE-2** (right). (C) Dose response measurements of CBP levels by Western blot after 16 h treatment of LP1 cells with **dCE-2**. Western blot images used for quantification in Figure S4. (D) Time course measurements of CBP and EP300 levels following treatment of LP1 cells with 1 μM **dCE-2**. (E) Western blot measurements of CBP and EP300 levels in various cell lines following 16 h treatment with 1 μM **dCE-2**. (F) LP1 and MM1 swith 1 μM **dCE-2** compared to DMSO-treated cells. Highlighted are the most significantly altered proteins (black, abs(difference) > 1 and −Log_10_(FDR) > 5) and (in red) CBP, EP300, MYC, and BRD4.

To confirm that **dCE-2** induces CBP degradation through the expected PROTAC mechanism, we synthesized an analogue unable to bind CRBN by *N*-methylation of the thalidomide moiety (**10**, Figure 3A). As expected, this modification abrogated the degradation of CBP/EP300 (Figure 3A). Similarly, CBP degradation by **dCE-2** could be prevented through cotreatment with two structurally distinct CBP/EP300-BRD binders, **2**,^52^ and GNE-781,^35^ as well as with the CRBN ligand pomalidomide, confirming that degradation requires the engagement of both CBP-BRD and CRBN (Figure 3B, left). Additionally, CBP/EP300 degradation could also be blocked using the neddylation inhibitor MLN4924 and the proteasome inhibitor MG132 (Figure 3B, center and right). Furthermore, treatment with **dCE-2** led to a mild upregulation in *crebbp* and *ep300* expression as measured by RT-qPCR (Figure S3). **dCE-2** is a highly potent and efficient CBP PROTAC, able to reach a *D*_max_ > 85% with a DC_50_ of 40 nM in LP1 cells after 16 h (Figure 3C). CBP/EP300 degradation begins to occur within 2 h but requires 16–24 h to reach maximal degradation (Figure 3D). Furthermore, **dCE-2** is an active degrader across a wide range of cancer cell lines including an additional multiple myeloma cell line (MM1S), as well as the prostate cancer line LNCaP and the neuroblastoma line SH-SY5Y (Figure 3E). Interestingly, the bias for CBP degradation over EP300 was consistent across all cell lines.

**dCE-2** displayed antiproliferative effects in LP1 (GI_50_ = 1.5 μM) and MM1S (GI_50_ = 35 nM) cells at lower concentrations than both the parent inhibitor **2**, pomalidomide, and negative control **10** (Figure 3F), thus highlighting the advantage of protein degradation over simple inhibition. On the other hand, despite clear CBP degradation, **dCE-2** has a limited effect on the proliferation of LNCaP and SH-SY5Y cells, indicating that either faster or more complete degradation of CBP/EP300 is required to inhibit the growth of these cells (Figure S5 and accompanying text).

CBP and EP300 were identified in global proteomics as two of the most downregulated proteins in both LP1 and MM1S cells following 16 h treatment with 1 μM **dCE-2** (Figure 3G, H), confirming their degradation in an antibody-independent manner. Furthermore, MYC, a well-established downstream target of CBP and EP300, was also highly downregulated in both cell lines. In contrast, the level of BRD4, a common off-target of CBP/EP300-BRD inhibitors, was not changed (verified by Western blot, Figure S6), confirming the specificity of **dCE-2** over other BRD-containing proteins. In both cell lines, the most strongly downregulated proteins were ZFP91 and IKZF1/3, all known substrates of immunomodulatory imide drugs (IMiDs).^68^ Future work on modifying the CRBN binding moiety of **dCE-2** would be required to reduce the degradation of these proteins while maintaining the desired effects on CBP/EP300.

## Binary Affinity, Ternary Complex, and Cooperativity

**Figure 4:**
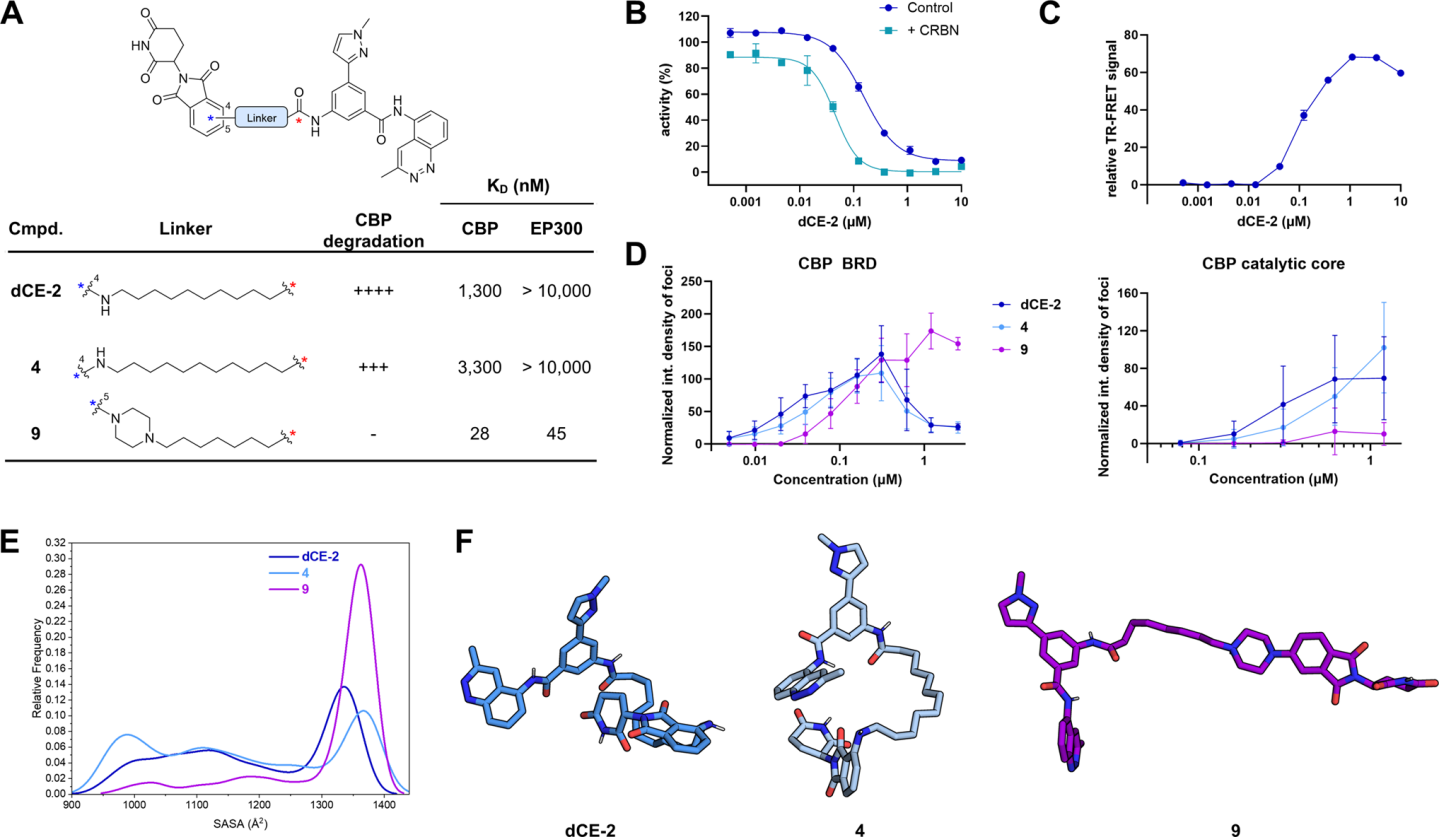
Mechanistic studies. (A) Chemical structures, CBP degradation efficiency, and binary affinities to CBP- and EP300-BRDs of selected compounds. *K*_D_ values were determined using BROMOscan technology. (B) **dCE-2** binding to CBP-BRD in the presence and absence of high concentrations of CRBN, as determined through competition with acetylated peptide binding using TR-FRET. (C) CBP-BRD:**dCE-2**:CRBN ternary complex formation as determined by TR-FRET. (D) Cellular ternary complex formation as determined by the FluoPPI technology using the CBP-BRD or CBP catalytic core. (E, F) Explicit water MD simulations of **dCE-2**, **4**, and **9**: (E) distribution of solvent accessible surface area (SASA) values along the 2.5 μs sampling of each PROTAC molecule and (F) representative conformer of the most populated cluster.

The binding affinity of **dCE-2** to the CBP- and EP300-BRDs was determined through a commercial service utilizing a ligand binding site-directed competition assay (BROMOscan, Figure 4A). A significant loss of potency compared to the parent compound **3** (>40-fold for CBP and >400-fold for EP300) and a slight preference toward CBP binding was observed in these measurements. We subsequently determined the CBP-BRD IC_50_ using an in-house TR-FRET-based competition assay, confirming the modest affinity of this PROTAC by another method (IC_50_ = 150 nM, Figure 4B). However, the binding of **dCE-2** was significantly improved in the presence of high concentrations of the CRBN C-terminal thalidomide binding domain (ternary IC_50_ = 45 nM, Figure 4B), demonstrating that formation of the CBP:**dCE-2**:CRBN ternary complex has good positive cooperativity (α = 3.4). This is in contrast to the highly active CBP/EP300 PROTAC dCBP-1^42^ which did not show any cooperativity in this assay (Figure S7). We were also able to observe robust formation of the ternary complex using CBP-BRD and CRBN-thalidomide binding domain labeled with a TR-FRET pair. This assay afforded a classical hook curve with a good peak height at around 1 μM **dCE-2** (Figure 4C), which is consistent with a low-affinity compound displaying positive cooperativity.^23^ As expected, the negative control compound **10** did not show cooperativity or ternary complex formation (Figure S8). Together, this biochemical data support the ability of **dCE-2** to act as a PROTAC despite its modest (high nanomolar–low micromolar) affinity for CBP.

It is apparent that **dCE-2** is an efficient PROTAC functioning through the expected mechanism; however, its binary affinity to CBP is modest, especially in comparison to the parent small-molecule ligand **3**. To explore which factors may affect affinity, *K*_D_ values (BROMOscan) were determined for all the PROTACs summarized in Figure 2A (Figure S9). This revealed that despite having the same moiety for the bromodomain and the same moiety for cereblon, the *K*_D_ of these PROTACs is highly variable and very sensitive to subtle changes in the linker length and composition. Interestingly an inverse correlation between the *K*_D_ and degradation ability was apparent (Figure S9), with the two potent degraders (**dCE-2** and **4**) showing the strongest cooperativity (for cooperativity values of all compounds in this study, see Figure S10).

We then turned our attention to the ability of these molecules to successfully form a ternary complex *in cellulo*. Thus, **dCE-2** and **4**—the two active degraders with modest (high nanomolar–low micromolar) affinity—were measured together with **9**, which shows low nanomolar affinity but was unable to induce degradation of CBP/EP300 (Figure 4A), in a ternary complex formation assay using FluoPPI^69^ (fluorescent-based technology detecting protein–protein interaction). This method enables ternary complex formation to be observed in live cells through the formation of fluorescent foci. Despite their differences in affinity and ability to induce degradation, **dCE-2**, **4**, and **9** were all able to induce good ternary complex formation in cells with CRBN and CBP-BRD (Figure 4D). Interestingly, the peak position of the hook curve, which is dependent upon a combination of the binary affinities for the CBP-BRD and CRBN,^23^ occurs at a higher PROTAC concentration for **9**. Assuming that the CRBN affinity of **9** is not significantly worse than **dCE-2** and **4**, this suggests that the effective concentration of **9** is lower in cells, indicating relatively poor membrane permeability of this compound. Together, this data with the CBP-BRD cannot explain the difference in degradation ability of these compounds. In contrast, only the active degraders **dCE-2** and **4**, but not the inactive PROTAC **9**, are able to induce ternary complex formation with the CBP catalytic core (Figure 4D). This highlights that regions of the CBP protein beyond the BRD are involved in ternary complex formation, and thus suggests that the increased rigidity afforded by the piperazine group in **9** may hamper viable ternary complex formation in this case.

As the CBP-binding moiety is identical for these PROTACs, with the variations in linker occurring far from the bromodomain binding pocket, we hypothesized that the discrepancies in affinity could stem from differences in the intramolecular folding of the molecules. Furthermore, differences in folding would also contribute to cellular permeability by masking H-bond donors and acceptors and reducing the surface area of the molecule.^70^ Thus, MD simulations were performed to map the conformations adopted by these PROTACs and their solvent accessible surface area (SASA) in an aqueous environment. The SASA correlates with extended (higher values) and compact (lower values) conformations of the molecule. Cluster analysis shows that **dCE-2** and **4** populate both compact and extended conformations, with 42 and 36% of the conformers having a SASA larger than 1300 Å^2^, respectively. In contrast, compound **9** predominantly populates extended conformations with 69% of the conformers having a SASA higher than 1300 Å^2^. These differences can explain, at least in part, the less favorable *K*_D_ values of **dCE-2** as its compact folding in water may impair binary binding in biochemical assays but aid its cell permeation. On the other hand, the flexible linker of **dCE-2** can allow the population of extended conformations required for productive ternary complex formation. These results are in line with previous studies in which PROTACs with a chameleonic behavior—i.e., the ability to mutate their conformation in environments with different polarity—showed improved aqueous solubility and cell permeability.^14^

## Conclusions

In this work, we report the discovery and characterization of a novel CBP/EP300 degrader **dCE-2**. This PROTAC is based on an in-house-developed CBP/EP300 ligand, **3** (*K*_D_ CBP/EP300 = 29/25 nM). The development of the small-molecule ligand **3** was based on an unprecedented 3-methylcinnoline acetyl-lysine mimic identified by high-throughput docking, followed by fragment growing and subsequent optimization based on the crystal structure of a closely related analogue. Our protein structure-based analysis enabled the identification of a suitable attachment point within this ligand, which upon connection to a 10-atom aliphatic linker and a thalidomide CRBN E3 ligand resulted in **dCE-2**. Interestingly, this PROTAC is active across multiple cell lines (LP1, MM1S, LNCaP, and SH-SY5Y) reaching its peak performance after 16 h (DC_50_ = 40 nM in LP1 cells). Furthermore, we show that **dCE-2** can form a ternary complex with CBP and CRBN both *in cellulo* (FluoPPI) and *in vitro* (TR-FRET) with high cooperativity (α = 3.4). Notably, MD simulations helped us rationalize why despite the modest *K*_D_ values of **dCE-2** toward CBP/EP300 bromodomains, this PROTAC could degrade both proteins in a highly efficient manner: its ability to switch between a compact and an extended conformation might impair binding in biochemical assays but guarantee improved cell permeability. Thus, in contrast to small-molecule inhibitor development, binary affinity should not be the only parameter in early PROTAC screening. Collectively, our results led to the development of a novel CBP/EP300 PROTAC that further expands the toolbox of chemical probes to deconvolute the role of such proteins in disease development. Furthermore, by combining biological, biochemical, and computational techniques, we shed light on the correlation between binding affinity and degradation of structurally close degraders, thus highlighting that a multidisciplinary approach is essential to fully understand PROTAC SAR.

## Supporting Information Available

Methods, general procedures, experimental procedures, and compound characterization (PDF)

## Abbreviations

PROTAC: proteolysis targeting chimera
CBP: CREB-binding protein
EP300: E1A-associated protein
HAT: histone acetyltransferase
BRD: bromodomain
Ac-lysine: acetylated lysine
LE: ligand efficiency
SAR: structure–activity relationship
FluoPPI: fluorescent-based technology detecting protein–protein interactions
POI: protein of interest
PPIs: protein–protein interactions
cryoEM: cryogenic electron microscopy
CRBN: cereblon
MD: molecular dynamics
SASA: solvent accessible surface area
TR-FRET: time-resolved fluorescence energy transfer
ZFP91: zinc finger protein 91
IKZF1/3: IKAROS family zinc finger 1/3
PEG: polyethylenglycol
h: hours
