# Supplementary material for: Structure-Based Design of CBP/EP300 Degraders: When Cooperativity Overcomes Affinity
Source: JACS Au. 2024 Aug 8;4(9):3466–74. doi: 10.1021/jacsau.4c00292 (PMC11423305; doi:10.1021/jacsau.4c00292)
Supplement: Supplementary file 1 — au4c00292_si_001.pdf [file au4c00292_si_001.pdf]

Structure-based Design of CBP/EP300 Degraders: When Cooperativity Overcomes Affinity

Iván Cheng-Sánchez,[a,‡] Katherine Gosselé,[a,b,‡] Leonardo Palaferri,[a,‡] Eleen Laul,[a] Gionata Riccabella,[a] Rajiv K. Bedi,[b] Yaozong Li,[b] Anna Müller,[b] Ivan Corbeski,[b] Amedeo Caflisch[b,*] and Cristina Nevado[a,*]

[a] Department of Chemistry, University of Zurich, Winterthurerstrasse 190, CH-8057 Zurich, Switzerland

[b] Department of Biochemistry, University of Zurich, Winterthurerstrasse 190, CH-8057, Zurich, Switzerland

[‡] These authors contributed equally

Supporting Information

Table of Contents

1. Supplementary Figures and Schemes S2
2. Methods and General Procedures S9
3. Experimental Procedures and Compound Characterization S17
4. ^{1}H and ^{13}C NMR Spectra S38
5. LCMS Data S67
6. References S70

1. Supplementary Figures and Schemes

Figure S1. Initial broad PROTAC screen.

Chemical structures of PROTAC molecules and quantification of CBP and EP300 protein by Western blotting following treatment of LP1 cells. Vinculin was used as a loading control for normalization.

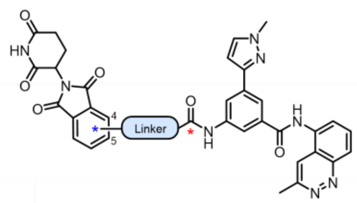

Cmpd	Linker	% remaining (5 µM, 16 h)	
		CBP	EP300
4	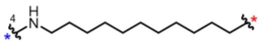	21	43
S1	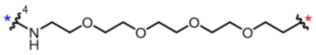	> 95	> 95
S2	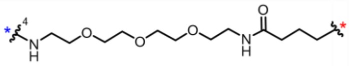	> 95	> 95
S3	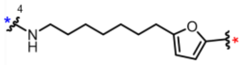	47	64
S4	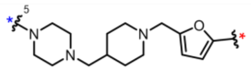	76	> 95

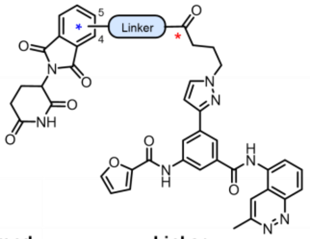

Cmpd	Linker	% remaining (5 µM, 16 h)	
		CBP	EP300
S5	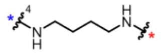	81	90
S6	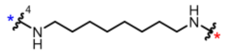	82	92
S7	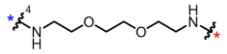	90	> 95
S8	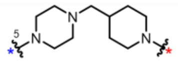	> 95	> 95
S9	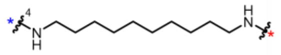	65	69

Images used for the quantification: Solid boxes encompass bands from same membrane, dashed lines show cropping of non-adjacent bands for clarity. For quantification, compound treated lanes were normalized to DMSO treated lanes on the same membrane. (Several compounds were measured at the same time, so DMSO treated lanes are replicated here between compounds).

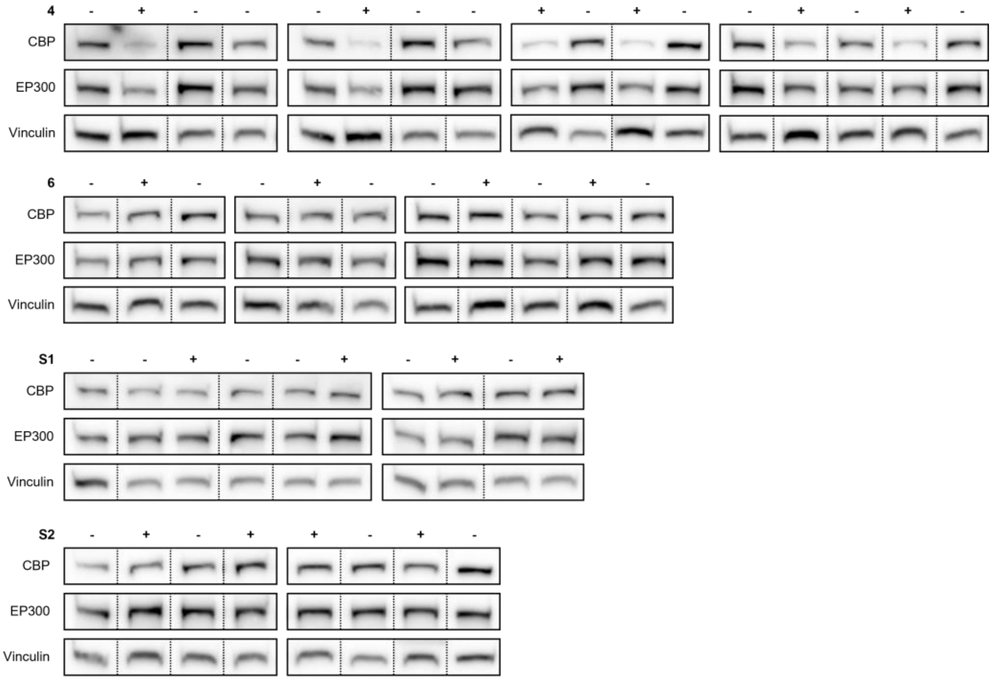

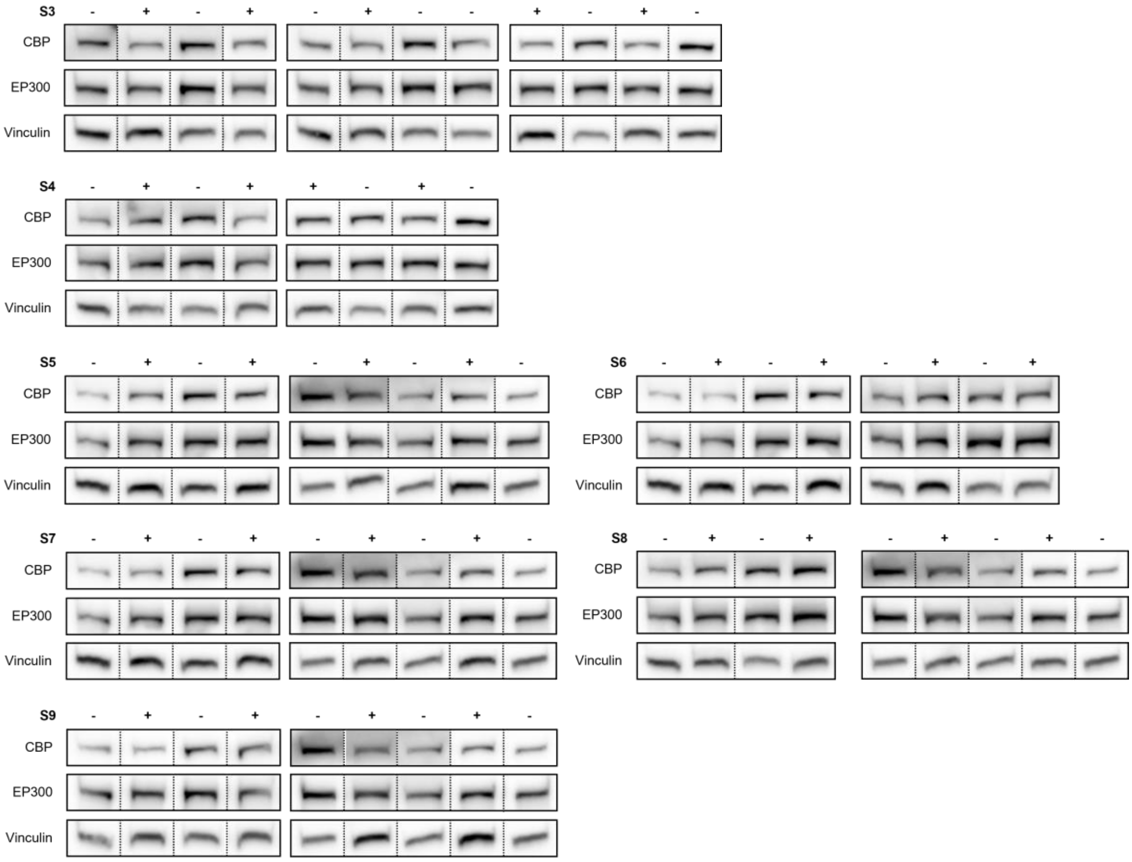
S3
CBP
EP300
Vinculin
S4
S5
S6
S7
S8
S9

Figure S2. Western blot images used for the quantification shown in Figure 2A.

Solid boxes encompass bands from same membrane, dashed lines show cropping of non-adjacent bands for clarity. For quantification, compound treated lanes were normalized to DMSO treated lanes on the same membrane. (Several compounds were measured at the same time, so DMSO treated lanes are replicated here between compounds). Compounds **4** and **6** were included in the initial screen (Figure S1).

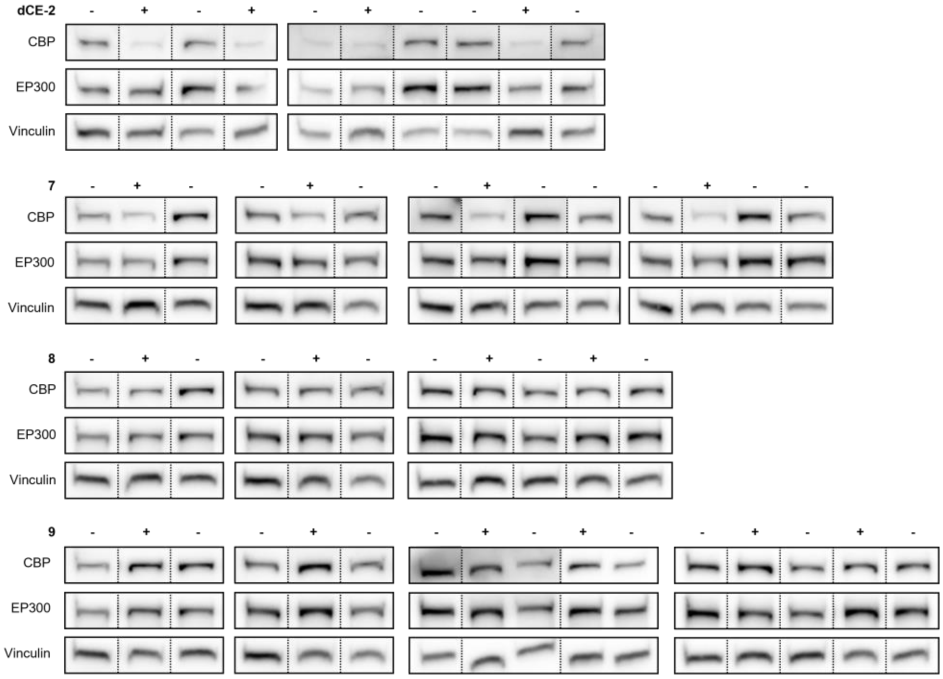

Figure S3: Gene expression analysis by RT-qPCR following treatment of LP1 cells for 16 h with 1 µM **dCE-2**. *myc* was included as a control to confirm the expected decreased in expression upon degradation of CBP/EP300.[1] Expression was normalized to *hprt*.

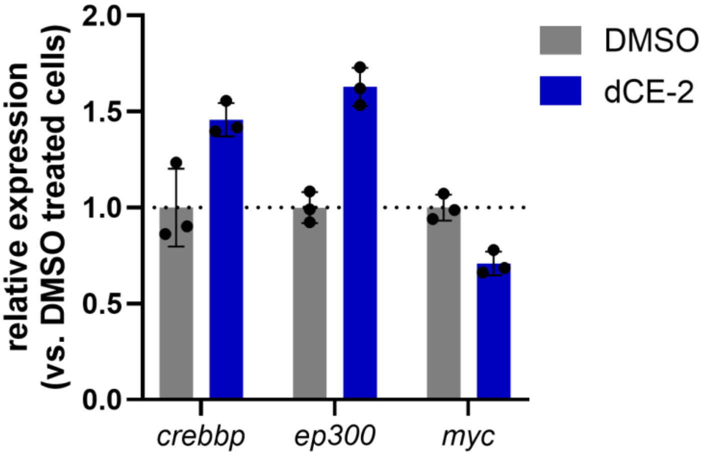

Figure S4. Western blot images used for the quantification of **dCE-2** shown in Figure 3C.

Solid boxes encompass bands from same membrane, dashed lines show cropping of non-adjacent bands for clarity. For quantification, compound treated lanes were

normalized to DMSO treated lanes on the same membrane. (Several compounds were measured at the same time, so DMSO treated lanes are replicated here between compounds).

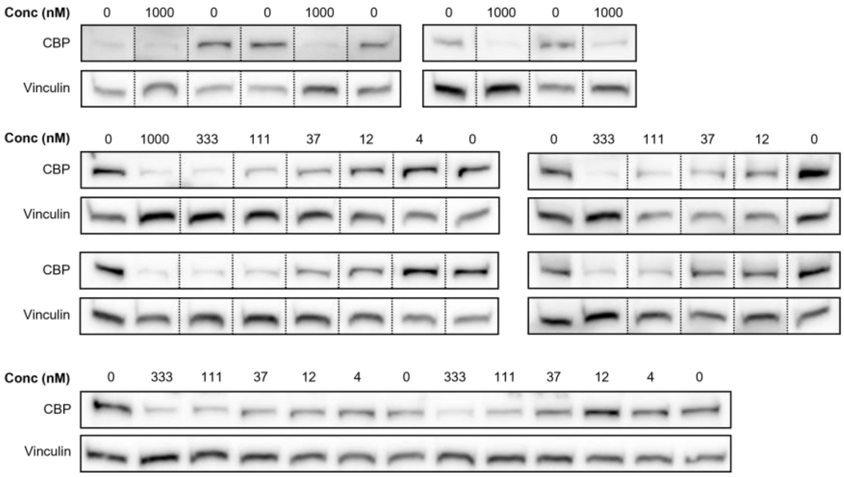

Figure S5. Anti-proliferation in additional cell lines. LNCaP (A and C) and SH-SY5Y (B) cell viability following 3 day compound treatment, and LNCaP following 6 day treatment (D), determined using resazurin.

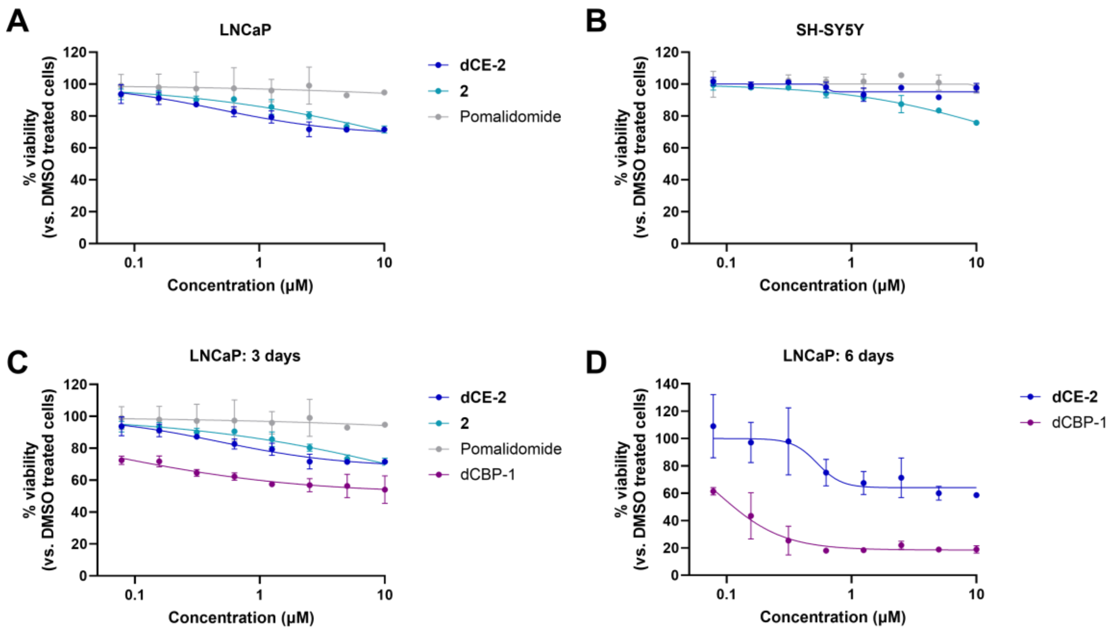

After 3 days of treatment, **dCE-2** only has a mild effect on the proliferation of LNCaP and SH-SY5Y cells (Figure S5A and B). These results are in contrast to recent studies using the new CBP/EP300 PROTACs CBPD-268 and CBPD-409[2,3] which have enhanced anti-proliferative activity in LNcaP cells relative to their parent inhibitors. However, although these PROTACs have effects at very low concentrations, their effect plateaus and a substantial proportion of cells remain viable at high compound concentrations. Similarly, our work with the first published CBP/EP300 PROTAC, dCBP-1, showed that it is able to inhibit proliferation at nM concentrations, but, despite its rapid and complete degradation of CBP and EP300, the maximal effect of this PROTAC is also limited under the conditions we used (Figure S5C). Through this work it was clear to us that these PROTACs did not elicit a toxic effect on cells, but instead blocked or reduced the rate of

proliferation. Thus, the extent of the effect on viability is highly dependent upon the growth of the DMSO treated cells during the treatment period, and so upon the timeframe of the treatment. As we used a relatively short timeframe, we repeated the treatment of LNCaP cells with **dCE-2** and dCBP-1 with a 6 day treatment and saw an increased effect on cell viability relative to DMSO treated cells with both compounds (Figure S5D). The plateau was reached at a lower cell viability for dCBP-1 than **dCE-2** which is not directly related to differences in their DC_{50} values but could be related to either: the rates of degradation (**dCE-2** D_{max} reached in 16-24 h vs. dCBP-1[1] D_{max} reached in 2-3 h, giving the cells differing opportunities to compensate for the reduction in these proteins; the preferential degradation of CBP vs. EP300 with **dCE-2** as the knockdown of EP300 has a stronger effect on LNCaP proliferation than CBP[3]; or the difference in D_{max} values where the small amount of remaining CBP/EP300 with **dCE-2** are sufficient to maintain a reduced but non-zero growth rate.

Figure S6. Western blot masurement of BRD4 levels in LP1 cells treated for 16 h with 1 μM **dCE-2**.

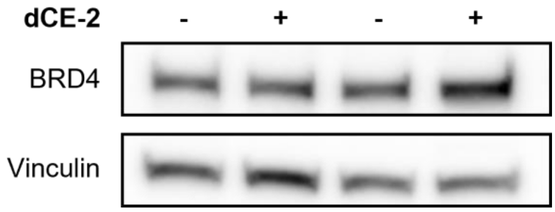

Figure S7: dCBP-1 binding to CBP-BRD in the presence and absence of high concentrations of CRBN, as determined through competition with acetylated peptide binding using TR-FRET. IC_{50}[control] = 1.6 nM, IC_{50}[CRBN] = 1.3 nM, α = 1.2.

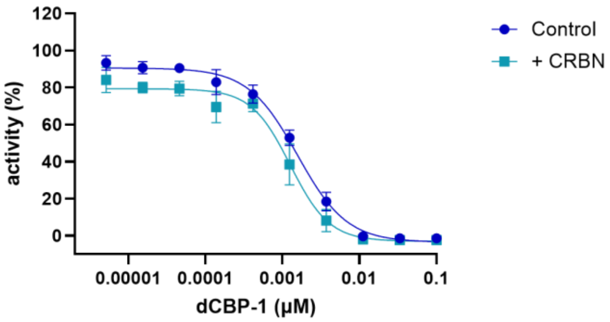

<u>Figure S8: A)</u> **10** binding to CBP-BRD in the presence and absence of high concentrations of CRBN, as determined through competition with acetylated peptide binding using TR-FRET. IC_{50}[control] = 220 nM, IC_{50}[CRBN] = 230 nM, α = 1.0. B) CBP-BRD:**10**:CRBN ternary complex formation as determined by TR-FRET.

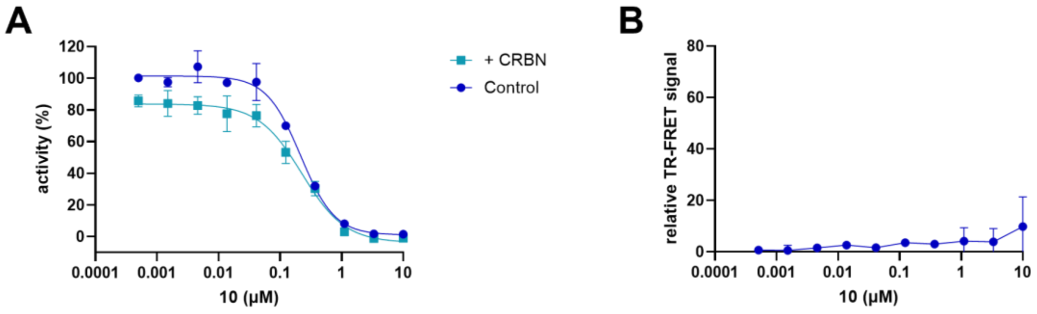

<u>Figure S9.</u> CBP and EP300 binding affinities and degradation of selected PROTACs (WB images in figures S1 an S2).

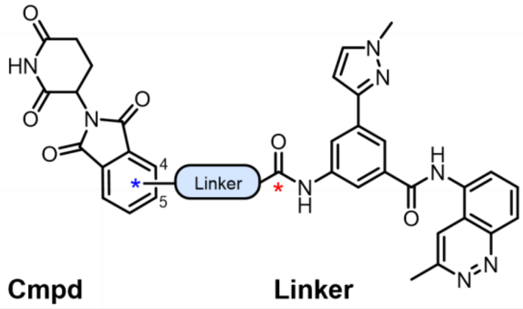

Cmpd	Linker	K_D (nM)		% remaining (5 µM, 16 h)	
		CBP	EP300	CBP	EP300
**4**	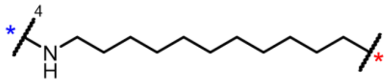	3,300	> 10,000	21	43
**dCE-2**	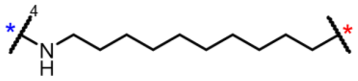	1,300	> 10,000	16	43
**7**	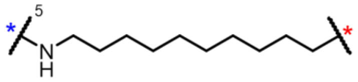	410	300	30	69
**6**	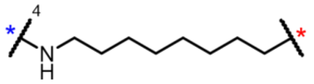	200	290	83	87
**8**	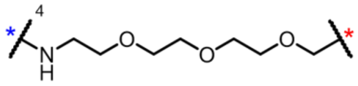	140	170	81	95
**9**	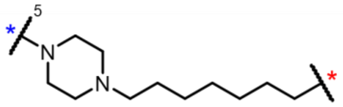	28	45	> 95	> 95

Figure S10: Binding to CBP-BRD in the presence and absence of high concentrations of CRBN, as determined through competition with acetylated peptide binding using TR-FRET.

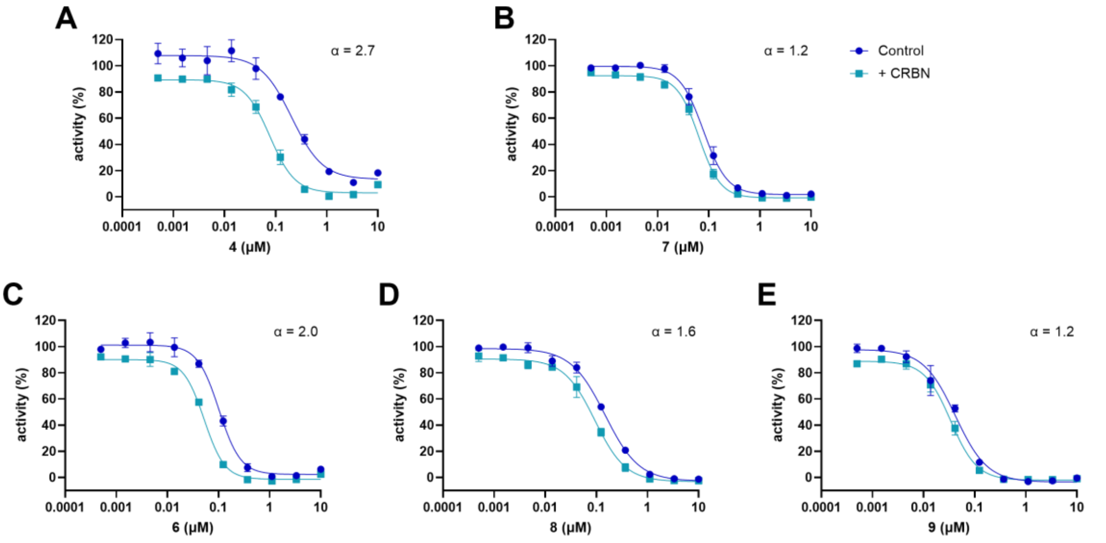

Scheme S1. Synthesis of warhead **S18.**

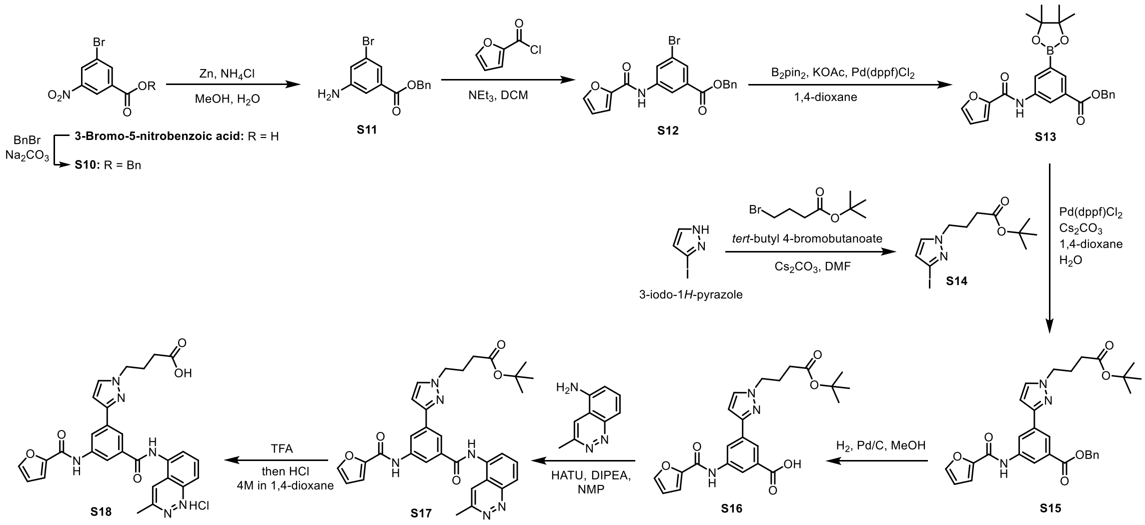

2. Methods and General Procedures

Reagents

MLN4924 was purchased from MedChemExpress (HY-70062), MG132 from Tocris (1748) and pomalidomide from Tokyo Chemical Industry co., ltd (P2074). All other compounds were synthesised in-house: dCBP-1,[1] GNE-781,[4] **1**[5] and **2**[5] were prepared in exactly the same manner as previously reported.

Protein crystallography

Proteins were cloned, purified, crystallized and data processed as previously reported.[5]

BROMO*scan*™

K_D values were determined using BROMO*scan*™ technology, carried out by Eurofins Discovery according to their standard protocol. Briefly *E. coli* were infected with T7 phage strains displaying bromodomains and bromodomain protein was subsequently purified through lysis, centrifugation and filtration. Affinity resins were generated by incubation of streptavidin-coated magnetic beads with biotinylated small molecules or acetylated peptide ligands. Binding reactions were assembled by combining the bromodomains and liganded affinity beads in binding buffer (17 % SeaBlock, 0.33x PBS, 0.04 % Tween 20, 0.02 % BSA, 0.004 % sodium azide and 7.4 mM DTT) with compounds (11-point, 3-fold dilution series) added to give a final DMSO concentration of 0.09 %. Assay plates were incubated for 1 h, prior to washing and elution of beads. The bromodomain concentration in the eluates was measured by qPCR of the DNA tag on the bromodomain.

K_D values were calculated with a standard dose-response curve fitted using the Hill equation:

$$Response = Background + \frac{Signal - Background}{1 + \left({K_D}^{Hill\ Slope} / Dose^{Hill\ Slope}\right)}$$

The Hill Slope was set to -1 and curves fitted using a non-linear least square fit with the Levenberg-Marquardt algorithm.

Ligand efficiency

Ligand efficiency (LE) was calculated as follows: LE = 1.4(pIC_{50})/N
N = Number of heavy atoms

TR-FRET

Cloning: GST-CBP (human, residues 1081-1197) was created by cloning the CBP sequence into the pGEX_6P_1 vector between the EcoRI and XhoI restriction sites using the His-CBP plasmid as a template (Addgene plasmid #38977, a gift from Nicola Burgess-Brown). His-CRBN (human, residues 318-436) was created by cloning into the pETDuet-1 vector between BamHI and XhoI restriction sites, using a template which was a gift from William Kaelin (Addgene plasmid #107374). His-CRBN-Avi was created by inserting a TEV cleavage site (GAGAATCTGTACTTTCAGTCA) to the N-terminus and an Avi-tag (GGGTTGAATGATATTTTCGAAGCACAGAAAATTGAATGGCATGAG) to the C-terminus of CRBN (human, residues 318-442). The construct was cloned into the pETDuet-1 vector between the SacI and HindIII restriction sites. Full-length BirA was cloned into the same vector between the NdeI and XhoI restriction sites.

Protein expression and purification

The GST-CBP, His-CRBN and His-CRBN-Avi constructs were overexpressed in Rosetta (DE3) cells upon induction with 100 μM isopropyl thio-beta-D-galactoside (IPTG) for 20 h at 18 °C. For His-CRBN-Avi, the expression media was supplemented with 50 μM D-biotin. Harvested cells were resuspended in lysis buffer (100 mM Tris-HCl pH 8.0, 500 mM NaCl, 1 mM 1,4-dithiothreitol (DTT), 1 mM phenylmethylsulfonyl fluoride

(PMSF), and 1 mM EDTA) and lysed by sonication. The lysates were clarified by centrifugation (18,000 rpm, 4 °C, 1 h).
For GST-CBP the soluble proteins were loaded onto a column packed with glutathione sepharose 4B (GE Healthcare) and subsequently eluted with 10 mM reduced glutathione in buffer containing 20 mM Tris-HCl pH 8.0, and 150 mM NaCl. Finally, a size exclusion step (HiLoad 16/600 Superdex 200 pg column from GE Healthcare) was used to further purify the protein in buffer containing 50 mM HEPES pH 7.5 and 150 mM NaCl. For His-CRBN and His-CRBN-Avi(biotin), the soluble fractions were loaded onto the HisTrap FF crude column (GE Healthcare) and washed with lysis buffer supplemented with 50 mM imidazole. The proteins were eluted with a buffer containing 250 mM imidazole, 100 mM Tris-HCl pH 8.0 and 500 mM NaCl. For His-CRBN-Avi(biotin) only, recombinant TEV protease was used to cleave the His6-tag during overnight dialysis at 4 °C against buffer containing 100 mM Tris-HCl pH 8.0 and 500 mM NaCl. The dialyzed sample was passed through the HisTrap FF crude column to remove His6-tagged TEV protease and uncleaved protein. In the final step for both His-CRBN and CRBN-Avi(biotin), the size-exclusion HiLoad 16/600 Superdex 200 pg column (GE Healthcare) was used to further purify the protein in 50 mM HEPES pH 7.5, 150 mM NaCl buffer, and for CRBN-Avi(biotin) this also removed the remaining traces of BirA. Each protein was aliquoted, flash frozen in liquid N_2 and stored at −80 °C until use.

HTRF measurements:
All reagents were dissolved in the assay buffer (50 mM HEPES pH 7.5, 150 mM NaCl, 0.1 % BSA). The HTRF donor, the anti-GST Eu^{3+}-labelled antibody (Cisbio, 61GSTKLB) was applied in a constant concentration across all experiments, and the concentration of the HTRF donor, the XL665-conjugated streptavidin (Cisbio, 610SAXLB) was adjusted to maintain the ratio of 1:8 to the biotinylated molecules in order to maintain the detection portion constant during the titrations.[6]
For the CBP-binding and cooperativity measurements, the final assay set up was as follows: 0.1 % DMSO, 10 nM GST-CBP, 60 nM SK-20 (SGRGK(Ac)GGK(Ac)GLGK(Ac)GGAK(Ac)RHRK-biotin), 100 mM KF, 7.5 nM XL665-Streptavidin conjugate and 0.8 nM anti-GST mAb – Eu^{3+}-cryptate. Compounds and His-CRBN were serially diluted in assay buffer to the following concentrations:

**[PROTAC] (µM)**	10	3.3	1.1	0.37	0.12	0.041	0.014	0.0046	0.0015	0.0005
**[His-CRBN] (µM)**	10	4.0	2.0	1.3	1.1	1.037	1.012	1.0041	1.0014	1.0005

The maximal control contained no PROTAC, the blank contained no SK-20 and no PROTAC – these were replaced by the appropriate buffer. Reactions were set up by addition of components as follows: (1) GST-CBP; (2) compound dilution series or DMSO in assay buffer (blank); (3) His-CRBN dilution series or assay buffer; (4) SK-20 or assay buffer (blank); detection buffer. Assays were carried out in duplicate on a Corning 384 U Bottom White Polystyrene plate. The samples were incubated for 3 h (20 °C, dark) and the signal was detected using the Infinite M1000 plate reader (Tecan). After excitation at 317 nm, emission at 620 and 665 nm was recorded. The ratio of the emissions, $F = \frac{\text{acceptor}_{665\text{ nm}}}{\text{donor}_{620\text{ nm}}}$, was considered for further analysis. The activity values of each compound were determined according to the following equation:

$$activity\ (\%) = \frac{\Delta F}{\Delta F_{DMSO}} * 100 = \frac{F-F_b}{F_t-F_b} * 100\,,$$

where F_t is the TR-FRET signal in the absence of any compound (maximal control, 100 % activity), F_b the TR-FRET signal in the absence of the GST-CBP (blank, 0 % activity) and F the TR-FRET signal in the presence of the compound. Dose-response curves were plotted in GraphPad Prism 10.2.3 and fitted with nonlinear regression “log(inhibitor) vs. normalized response – variable slope”, from which IC_{50} values were determined. The

cooperativity factor (α) was defined as the ratio of the IC_{50} values in the absence and presence of His-CRBN.

For the ternary complex experiments, the final assay composition was: 0.1 % DMSO, 5 nM GST-CBP, 5 nM CRBN-Avi(biotin), 100 mM KF, 0.625 nM XL665-Streptavidin conjugate and 0.8 nM anti-GST mAb – Eu^{3+}-cryptate. The maximal control contained 40 nM dCBP-1, the blank contained no PROTAC. Hook curves were generated by normalization to the maximal control.

Cell culture

LP1 cells were cultured in DMEM (Gibco, 41966-029) supplemented with 15 % FBS (Sigma, F9665); MM1S and LNCaP cells in RPMI 1640 ATCC modification (Gibco, A1049101) supplemented with 10 % FBS (Gibco, 10270106); SH-SY5Y cells in DMEM/F12 (Gibco, 11320074) supplemented with 1 % non-essential amino acids (Gibco, 11140-035) and 15 % FBS (Gibco, 10270106); and U2OS cells in DMEM (Gibco, 41966-029) supplemented with 10 % FBS (Gibco, 10270106). All cell line media were additionally supplemented with 1 % penicillin/streptomycin (Gibco, 15070-063), and cells maintained in a humidified incubator with 5 % CO_2 at 37 °C. The identity of all lines (except for MM1S which were recently purchased from ATCC) was authenticated by STR profiling (performed by Microsynth).

Quantification of CBP/EP300 degradation by Western blotting

LP1 cells plated on 6 well plates were treated with the stated compounds at a final concentration of 0.1 % DMSO for the stated time. Cells were harvested on ice, washed in PBS and lysed in RIPA buffer (50 mM tris pH 7.5, 2 mM EDTA, 150 mM NaCl, 0.5 % sodium deoxycholate, 1 % Triton X100) supplemented with cOmplete™ protease inhibitor cocktail (Roche). Protein concentrations were determined using the Pierce™ BCA Protein Assay Kit (Thermo Scientific, 23227) and equal amounts were separated on 4-12 % ExpressPlus™ PAGE gels (GenScript, M41215). Proteins were transferred to PVDF membranes, the membranes were blocked with 5 % milk (Merk Millipore, 115363) in PBS-T and proteins were stained with the following antibodies at 4 °C overnight: CBP (Santa Cruz, sc-7300, diluted 1:200), EP300 (Cell Signalling, D2X6N, diluted 1:1000), BRD4 (Bethyl Laboratories, A301-985A50, diluted 1:2000) and vinculin (Invitrogen, 42H89L44, diluted 1:1000). For detection, membranes were incubated for 1 h at room temperature with HRP-conjugated secondary antibodies (Southern Biotech, anti-rabbit 6415-05 and anti-mouse 6440-05, both 1:5000 dilution), and were imaged using SuperSignal™ West Pico PLUS Chemiluminescent Substrate (Thermo Scientific, 34580) and the Vilber Fusion-FX7 imager. Bands were quantified using ImageJ software and protein levels normalized to protein from DMSO treated cells run on the same membrane. For dose response curves, data was fitted using a four-parameter dose-response curve in GraphPad Prism v. 9.5.1, where the top is fixed at 100 %. The DC_{50} was defined as the PROTAC concentration resulting in 50 % remaining protein.

RT-qPCR

LP1 cells plated on 6 well plates were treated with the stated compounds at a final concentration of 0.1 % DMSO for the stated time. RNA was extracted using the NucleoSpin® RNA kit (Machery-Nagel) and 500 ng used for cDNA synthesis with the High-Capacity cDNA Reverse Transcription kit (Applied Biosystems). RT-qPCR reactions were performed using *Power*SYBR™ green PCR Master Mix (Applied Biosystems) and a LightCycler® 480 (Roche). cDNA was denatured for 10 min at 95 °C followed by 40 cycles of 15 s at 95 °C and 1 min at 60 °C. C_p values were determined as the maximum of the second derivative and target gene expression was compared to that of *hprt* from the same sample using the $2^{-\Delta Cp}$ method. Gene expression was then normalized to the average of the DMSO treated samples.

The primer sequences used were:

Gene Name	Gene ID	Forward Primer (5'-3')	Reverse primer (5'-3')
hprt	NM_000194.3	CCCTGGCGTCGTGATTAGTG	TCGAGCAAGACGTTCAGTCC
myc	NM_001354870.1	GTAGTGGAAAACCAGCC	AGAAATACGGCTGCACCGAG
crebbp	NM_004380.3	TGTCACAGGGACAGGTGC	TGTCGTGTGCTGGAGAGATG
ep300	NM_001429.4	CAGCCTGCAACTCCACTTTC	AGGCTGCTTCTCAGCAATGG

Cell viability
Cells were plated onto 96 well plates at the following densities in 90 µl volume: $3x10^4$ LP1 cells/well or $5x10^3$ LNCaP cells/well both plated same day as treatment, or $2x10^4$ MM1S cells/well or $1.5x10^3$ SH-SY5Y cells/well both plated day prior to treatment. Compounds were added in 10 µl to give the stated concentrations with a final concentration of 0.1 % DMSO. Following 3 days of compound treatment, resazurin (Acros Organics, 189900010) was added at a final concentration of 86 µM and incubated for the following times: MM1S 5 h, LP1 3 h, LNCaP 1 h 30 min, SH-SY5Y 6 h. Fluorescence (excitation = 560 nm, emission = 590 nm) was measured using a spectraMax M5 microplate reader (Molecular Devices) and cell viability was calculated relative to DMSO treated cells on the same plate. Data was fitted using a four-parameter dose-response curve in GraphPad Prism v. 9.5.1, where the top is fixed at 100 %. GI_{50} values were calculated as the concentration required to inhibit cell viability to 50 % relative to DMSO treated cells after 3 days.

Proteomics

Sample digestion and clean up: Treated cells were washed in PBS and cell pellets were then lysed in 4 % SDS/Tris-HCl, treated with High Intensity Focused Ultrasound (HIFU) for 1 min at an ultrasonic amplitude of 80 % and boiled at 95 °C for 10 min. Ten units of Benzonase were added after diluting SDS to 1 % followed by centrifugation at 20000 x g for 10 min. The protein concentration was estimated using the Lunatic UV/Vis polychromatic spectrophotometer (Unchained Labs). For each sample, 10 µg of proteins were reduced with 2 mM TCEP (tris(2-carboxyethyl)phosphine) and alkylated with 15 mM chloroacetamide at 30 °C for 30 min. Samples were processed using the single-pot solid-phase enhanced sample preparation (SP3). The SP3 protein purification, digest and peptide clean-up were performed using a KingFisher Flex System (Thermo Fisher Scientific) and Carboxylate-Modified Magnetic Particles (GE Life Sciences; GE65152105050250, GE45152105050250).[7,8] Beads were conditioned following the manufacturer's instructions, consisting of 3 washes with water at a concentration of 1 µg/µl. Samples were diluted with 100 % ethanol to a final concentration of 50 % ethanol. The beads, wash solutions and samples were loaded into 96 deep well- or micro-plates and transferred to the KingFisher. The following steps were carried out on the robot: collection of beads from the last wash, protein binding to beads, washing of beads in wash solutions 1-3 (80 % ethanol), protein digestion (overnight at 37 °C with a trypsin:protein ratio of 1:50 in 50 mM Triethylammoniumbicarbonat (TEAB)) and peptide elution from the magnetic beads using MilliQ water. The digest solution and water elution were combined and dried to completeness.

TMT labelling and peptide fractionation: 50 µg TMTpro 18-plex reagent (Thermo Fisher Scientific) was dissolved in 5 µl of anhydrous acetonitrile (Sigma-Aldrich) and added to 10 µg peptides in 15 µl of 50 mM TEAB, pH 8.5. The solution was gently mixed and incubated for 60 min at room temperature. The reaction was quenched by adding

1.2 µl of 5 % hydroxylamine (Thermo Fisher Scientific). The combined TMT sample was created by mixing equal amounts of each TMT channel together. Labeled peptides were offline pre-fractionated using high pH reverse phase chromatography. Peptides were separated on an XBridge Peptide BEH C18 column (130 Å, 3.5 µm, 4.6 mm X 250 mm, Waters) using a 72 min linear gradient from 5-40 % acetonitrile / 9 mM NH_4HCO_2. Every minute a new fraction was collected and concatenated into 12 final fractions.

LC-MS/MS analysis: Mass spectrometry analysis was performed on an Orbitrap Exploris 480 mass spectrometer (Thermo Fisher Scientific) equipped with a Nanospray Flex Ion Source (Thermo Fisher Scientific) and coupled to an M-Class UPLC (Waters). Solvent composition at the two channels was 0.1 % formic acid for channel A and 0.1 % formic acid, 99.9% acetonitrile for channel B. Column temperature was 50 °C. Peptides were loaded on a commercial nanoEase MZ Symmetry C18 Trap Column (100 Å, 5 µm, 180 µm x 20 mm, Waters) connected to a nanoEase MZ C18 HSS T3 Column (100 Å, 1.8 µm, 75 µm x 250 mm, Waters). Peptides were eluted at a flow rate of 300 nL/min. After a 3 min initial hold at 5 % B, a gradient from 5 to 22 % B in 80 min and 22 to 32 % B in additional 10 min was applied. The column was cleaned after the run by increasing to 95 % B and holding 95 % B for 10 min prior to re-establishing loading condition for another 10 min.The mass spectrometer was operated in data-dependent mode (DDA) with a maximum cycle time of 3 s, with spray voltage set to 2.6 kV, funnel RF level at 40 % and heated capillary temperature of 275 °C. Full-scan MS spectra (350−1’500 m/z) were acquired at a resolution of 120’000 at 200 m/z after accumulation to a target value of 3’000’000 or for a maximum injection time of 45 ms. Precursors with an intensity above 5’000 were selected for MS/MS. Ions were isolated using a quadrupole mass filter with 0.7 m/z isolation window and fragmented by higher-energy collisional dissociation (HCD) using a normalized collision energy of 32 %. HCD spectra were acquired at a resolution of 30’000 with turboTMT on and maximum injection time was set to Auto. The normalized automatic gain control (AGC) was set to 100 %. Charge state screening was enabled such that singly, unassigned and charge states higher than six were rejected. Precursor masses previously selected for MS/MS measurement were excluded from further selection for 20 s, and the exclusion window was set at 10 ppm. The samples were acquired using internal lock mass calibration on m/z 371.1012 and 445.1200.
The mass spectrometry proteomics data were handled using the local laboratory information management system (LIMS).[9]

Data analysis: The acquired shotgun MS data were processed for identification and quantification using Fragpipe 19.0 (Philosopher 4.8.1).[10] Spectra were searched against a Uniprot *Homo sapiens* reference proteome (reviewed canonical version from 2023-03-30, concatenated to its reversed decoyed fasta database and common protein contaminants) using MSFragger 3.6 and Percolator. Proteins were identified using at least one peptide. TMT modification on peptide N-termini and Lysine side chains as well as carbamidomethylation of cysteine were set as fixed modification, while methionine oxidation was set as variable. Enzyme specificity was set to trypsin/P allowing a minimal peptide length of 7 amino acids and a maximum of two missed cleavages. Reporter ion intensities were extracted with 20 ppm integration tolerance. For peptide and protein quantification the co-isolation filter was set to 50 %. The R package prolfqua[11] was used to analyze the differential expression and to determine group differences, confidence intervals, and false discovery rates for all quantifiable proteins. Starting with the psm.tsv file generated by FragPipe, which does report the peptide spectrum match abundances (psm) for each TMT label, we filtered the psm's by purity_threshold = 0.5, PeptideProphetProb = 0.9. Next, we determined protein abundances by aggregating the psm abundances to peptidoform abundances and then employed the Tukeys-Median Polish to estimate protein abundances. Furthermore, before fitting the linear models, we transformed the protein abundances using the variance stabilizing normalization.[12] To analyze the differential expression, data was fitted to a linear model that explains the

observed protein abundances using the R function *lm* for each protein, with samples grouped by compound treatment. Differences between PROTAC treated and DMSO treated samples were computed, and a null hypothesis significance test was performed. Vaiance shrinkage was performed[13] to increase the power of the analysis, and finally the false discovery rate (FDR) was computed using the Benjamini-Hochberg procedure.[14]

Fluorescent-based technology detecting protein-protein interactions (FluoPPI)
Full length human CRBN was cloned into the phAG-MCL plasmid and the CBP-BD (residues 1081-1211) or the CBP catalytic core (residues 1081-1700) were cloned into the pAsh-MCL plasmid (plasmids from MBL, AM-8011M). These plasmids were transfected in a 1:1 ratio into U2OS cells using lipofectamine, 24 h before re-plating onto 96 well plates. Transfected cells were treated with compounds for 6 h, before fixation with 4 % PFA and the nuclei stained with DAPI. Images of foci and nuclei were obtained using an Evos M7000 at 20x magnification using GFP and DAPI light cubes respectively. Image J was used for quantification. Nuclei were counted by subtracting the background, before thresholding using the Huang algorithm, running the watershed algorithm, and counting particles with a size of 400 – infinity pixels. The integrated density of foci was calculated by thresholding using the Intermodes algorithm to select regions with an area of 3 – 1000 pixels and a circularity ≥ 0.8, which were mapped back to the original image to find the integrated density. Finally, normalized density was calculated by dividing foci integrated density by nuclear count, before normalizing to the response to 0.5 µM (BD) or 2 µM (catalytic core) dCBP-1 measured on the same plate to correct for differences between experimental runs.

SEED
SEED is an open-source code for high-throughput fragment docking into a rigid protein structure (https://gitlab.com/CaflischLab/SEED). A library of 419 heteroaromatics[15] was docked by SEED into the crystal structure of the CBP bromodomain (PDB code 3P1C) and the BRD4(1) bromodomain (PDB code 3MXF). The SEED docking of 419 fragments required about 15 minutes of a single core of a Xeon® Processor E3-1245 at 3.5 GHz. The CHARMM36[16] and CGenFF force fields[15] were used for the protein and fragments, respectively. The SEED total energy consists of van der Waals energy and electrostatic energy. The latter is the sum of protein (i.e, bromodomain) desolvation, fragment desolvation, and intermolecular interaction screened by the solvent which is treated implicitly by an efficient numerical implementation of the generalized Born approximation.[17] The value of the dielectric constant was 2 and 78.5 for the regions of the volume occupied by the solute and solvent, respectively.

Molecular dynamics simulations
We used a standard molecular dynamics (MD) protocol as follows. The 3D models of eight PROTAC molecules were built in Maestro,[18] and their protonation states were determined via MarvinSketch.[19] They were then solvated with a 60 Å rhombic dodecahedron (RHDO) water box to ensure at least a 12 Å buffer water layer between the system and the boundary of the water box. A modified TIP3P model[20] was used to describe the bulk water. Each simulation system contained Na^+ and Cl^- ions to approximate an ionic strength of 150 mM. The PROTAC molecules were minimized and equilibrated with CHARMM (version c42b2)[21] using the CHARMM CGenFF force field.[15] The equilibration phases lasted 1 ns in the NPT ensemble. Electrostatic interactions were evaluated using the particle mesh Ewald summation (PME) method,[22] and truncation of all nonbonded interactions occurred at 12Å. Production MD simulations were performed using the NAMD program (version 2.13)[23] in the NPT ensemble with a temperature of 300 K and pressure of 1 atm kept constant by the Nosé–Hoover Langevin piston method with a 200 ps piston period and 100 ps piston decay time.[24,25] The length of the covalent bonds involving hydrogen atoms was constrained by the SHAKE algorithm. The integration time step was set to 2 fs. Each system was replicated 10 times, and each

replica lasted 500 ns. The statistical distribution analysis (Kernel Smooth) was done with OriginPro 2021 (9.8.0.200).[26]

We used a clustering method to analyze the conformational diversity of each of three PROTAC molecules, i.e., **dCE-2**, **4** and **9**. For each PROTAC 250 snapshots saved every 20 ns along the cumulative sampling of 5 μs were selected (Boltzmann distributed). Clustering for each individual PROTAC was carried out by Root-Mean-Square Deviation (RMSD) matrix analysis. Specifically, we first mutually superposed the 250 snapshots to each other using their bromodomain binding moiety. We then calculated the RMSD value for each pair of snapshots, forming a 250×250 RMSD matrix. We then applied the clustering algorithm in the routine "CORREL" of CHARMM[21]to the RMSD matrix. The RMSD threshold value for clustering was set to 1 Å. As a result, approximately 10 clusters were obtained for each PROTAC molecule. Finally, the cluster centers were used as the representative conformations.

Characterization of compounds

NMR spectra were recorded on AV 300, AV2 400 or AV2 500 MHz Bruker spectrometers. The spectra are calibrated to the residual ^{1}H and ^{13}C signals of the solvents. Chemical shifts are reported in ppm with the resonance resulting from incomplete deuteration of the solvent as the internal standard. Multiplicities are abbreviated as follows: singlet (s), doublet (d), triplet (t), quartet (q), doublet-doublet (dd), quintet (quint), multiplet (m), and broad (bs). High resolution electrospray ionization mass spectrometry, HRMS (ESI), was performed on a Dionex Ultimate 3000 UHPLC system (ThermoFischer Scientifics, Germering, Germany) connected to a QExactive MS with a heated ESI source (ThermoFisher Scientific, Bremen, Germany); mass calibration to <2 ppm accuracy with Pierce® ESI calibration solns. (ThermoFisher Scientific, Rockford, USA). LC-MS analysis of small molecules (≤ ca. 2000 Da) was carried out by a less than 5 min HPLC gradient elution based on H_2O + 0.02 % TFA (mobile phase A1) and ACN + 0.02% TFA (mobile phase B1).

Synthetic and purification methods

Unless otherwise stated, reactions were carried out under a nitrogen atmosphere using standard Schlenk-techniques. All reagents were used as received unless otherwise noted. Solvents were purchased in the best quality available, degassed by purging thoroughly with nitrogen and dried over activated molecular sieves of appropriate size. Reactions were monitored by thin layer chromatography (TLC) using Merck TLC silica gel 60 F_{254} and using UV light (254 nm) as visualizing agent and acidic ceric ammonium molybdate/ phosphomolybdic acid or potassium permanganate solutions and heat as developing agents. Column chromatography was performed over silica gel (230-400 mesh) under nitrogen pressure. Reversed-phase HPLC purification was performed on a Shimadzu Nexera system, equipped with a an Agilent Zorbax 300SB-C18 semi-preparative column (9.4 x 250 mm 5-micron) using: a) 20 to 50% acetonitrile in water buffered with 0.1% trifluoroacetic acid as eluent (3.5 mL/min) or b) 20 to 50% acetonitrile in water buffered with 0.1% formic acid as eluent (8 mL/min). All biologically tested compounds are >95% pure by LC-MS.

General Procedures

General procedure 1: *t*Butyl ester removal followed by salt exchange

TFA (100 equiv) was added to a solution of a *t*butyl ester derivative (1.0 equiv) in DCM (0.1 M) and the resulting reaction mixture was stirred at 20 ºC for 30 min. After this time, the organic solvent and excess of TFA were removed under reduced pressure to afford the corresponding carboxylic acid trifluoroacetate salt which was used in the next step

without further purification. Crude carboxylic acid obtained above was dissolved into HCl (4M in 1,4-dioxane, 20 equiv) and the mixture was stirred at 20 ºC for 15 min. After this time the reaction mixture was concentrated under reduced pressure and triturated with diethyl ether and pentane to afford the corresponding carboxylic acid hydrochloric salt which was used in the next step without further purification.

General procedure 2. *N*-Boc deprotection

HCl (4 M in 1,4-dioxane, 80 equiv) was added to a solution of a *N*-Boc derivative (1.0 equiv) in DCM (0.1 M) while stirring at 0 °C. After 6 h, the organic solvent was removed by bubbling nitrogen gas for 15 min. Trituration with diethylether delivered the corresponding hydrochloric salt which was used in the next step without further purification.

General procedure 3A. PROTAC synthesis by amide coupling from S19

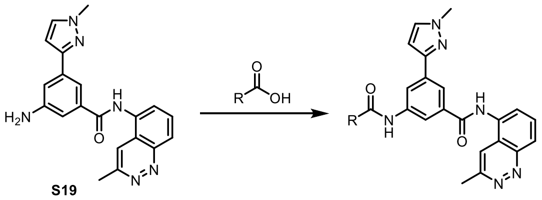

Carboxylic acid (1.2 equiv), HOBt·H_2O (1.1 equiv) and EDC·HCl (2.4 equiv) were dissolved in DMF (0.5 mL) and the resulting reaction mixture was stirred at 23 ºC for 1 h. After this time, the solution was transferred to a 5 mL round bottom flask containing a mixture of **S19**[5] (1.0 equiv) and proton sponge (3.0 equiv), and stirred at 23 ºC for 15 h. Then, the organic solvent was removed under reduced pressure to obtain a crude mixture which was purified by preparative TLC, followed by trituration to afford the corresponding PROTAC.

General procedure 3B. PROTAC synthesis by amide coupling from S18

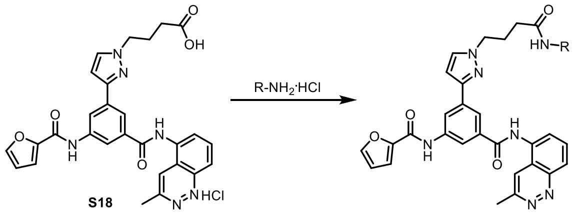

Carboxylic acid **S18** (1.0 equiv), HOBt·H_2O (1.1 equiv) and EDC·HCl (2.4 equiv) were dissolved in DMF (0.5 mL) and the resulting reaction mixture was stirred at 23 ºC for 1 h. After this time, the solution was transferred to a 5 mL round bottom flask containing a mixture of the amine salt (1.2 equiv) and proton sponge (3.0 equiv), and stirred at 23 ºC for 15 h. Then, the organic solvent was removed under reduced to obtain a crude mixture which was purified by preparative TLC, followed by trituration to afford the corresponding PROTAC.

3. Experimental Procedures and Compound Characterization

Compound 3

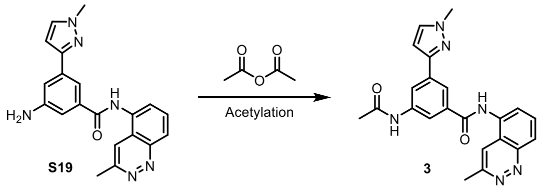

To **S19**[5] (10 mg, 0.028 mmol, 1.0 equiv) and triethylamine (5.6 mg, 0.056 mmol, 2.0 equiv) were added 0.28 mL of a solution of acetic anhydride (4.1 mg, 0.056 mmol, 2.0 equiv) in dry DMF (0.2 M) and the reaction mixture was stirred at 20 °C for 19 hours. After this time the reaction mixture was concentrated under reduced pressure and the crude purified by flash chromatography (2 to 10% MeOH in DCM) to afford **3** as a white solid (7.5 mg, 67%). ^{1}H NMR (500 MHz, DMSO-*d₆*) δ 10.67 (s, 1H), 10.18 (s, 1H), 8.36 – 8.33 (m, 1H), 8.31 (t, *J* = 1.8 Hz, 1H), 8.18 – 8.12 (m, 2H), 8.03 (s, 1H), 7.96 – 7.88 (m, 2H), 7.79 (d, *J* = 2.2 Hz, 1H), 6.74 (d, *J* = 2.3 Hz, 1H), 3.92 (s, 3H), 2.89 (s, 3H), 2.09 (s, 3H). ^{13}C NMR (126 MHz, DMSO-*d₆*) δ ^{13}C NMR (126 MHz, DMSO) δ 168.58, 166.42, 153.28, 149.30, 149.06, 139.87, 139.77, 135.12, 134.12, 133.25, 132.57, 129.61, 127.09, 126.69, 122.46, 119.10, 118.45, 117.75, 102.85, 38.74, 24.05, 21.70. HRMS (ESI): m/z calcd for $C_{22}H_{21}N_6O_2$ [M + H]$^+$: 401.17205, found 401.17163.

Compound S21

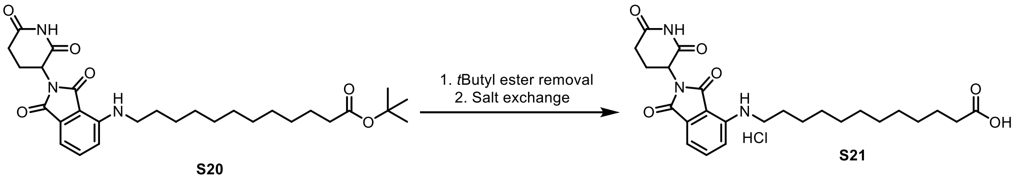

Compound **S21** (~0.243 mmol, yellow solid) was prepared from **S20**[27] (128 mg, 0.2430 mmol, 1.0 equiv) according to the general procedure 1 and used in the next step without further purification.

Compound 4

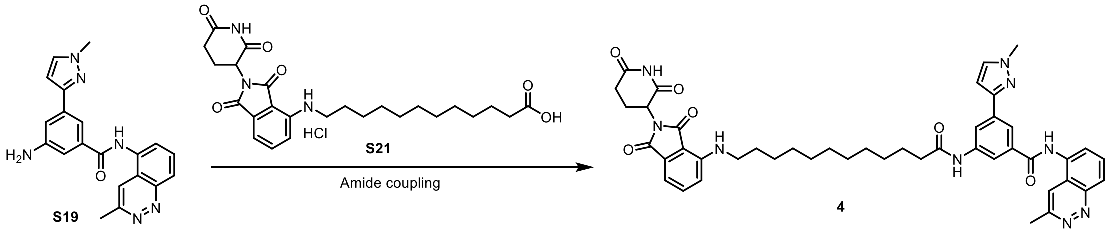

4 was prepared from aniline **S19**[5] (10 mg, 0.0279 mmol, 1.0 equiv) and **S21** (17 mg, 0.0341 mmol, 1.20 equiv) according to a modified general procedure 3A, by using DIPEA (0.016 mL, 0.0959 mmol, 3.44 equiv) and DMSO (0.3 mL) instead of proton sponge and DMF. The crude mixture was purified by preparative TLC (1:2:17 MeOH:EtOAc:DCM) followed by trituration with diethyl ether and pentane to afford **4** (7 mg, 31%) as a yellow solid. ^{1}H NMR (400 MHz, Methanol-*d₄*) δ 8.36 (dt, *J* = 8.5, 1.2 Hz, 1H), 8.26 (t, *J* = 1.8 Hz, 1H), 8.22 (t, *J* = 1.6 Hz, 1H), 8.18 (t, *J* = 1.8 Hz, 1H), 8.12 (t, *J* = 0.9 Hz, 1H), 8.00 (dd, *J* = 7.4, 1.2 Hz, 1H), 7.93 (dd, *J* = 8.6, 7.4 Hz, 1H), 7.65 (d, *J* = 2.3 Hz, 1H), 7.51 (dd, *J* = 8.5, 7.1 Hz, 1H), 7.00 (dd, *J* = 7.8, 3.5 Hz, 2H), 6.72 (d, *J* = 2.3 Hz, 1H), 5.04

(dd, J = 12.5, 5.5 Hz, 1H), 3.96 (s, 3H), 3.27 (d, J = 6.9 Hz, 2H), 2.94 (s, 3H), 2.90 – 2.77 (m, 1H), 2.78 – 2.66 (m, 2H), 2.44 (t, J = 7.4 Hz, 2H), 2.13 – 2.05 (m, 1H), 1.74 (q, J = 7.3 Hz, 2H), 1.62 (q, J = 7.1 Hz, 2H), 1.45 – 1.30 (m, 14H). ^{13}C NMR (100 MHz, DMSO-d_6) δ 172.82, 171.63, 170.11, 168.95, 167.31, 166.47, 153.30, 149.32, 149.07, 146.44, 139.90, 136.28, 135.12, 134.12, 133.27, 132.58, 132.19, 129.64, 127.12, 126.72, 122.48, 119.05, 118.50, 117.72, 117.19, 110.37, 108.99, 102.84, 48.54, 41.83, 36.43, 30.98, 30.71, 28.99, 28.97, 28.92, 28.80, 28.77, 28.67, 28.64, 26.31, 25.09, 22.16, 21.71, 15.18. HRMS (ESI): m/z: calcd. for $C_{45}H_{50}N_9O_6^+$ [M + H]$^+$: 812.38786, found: 812.38730.

Compound S23

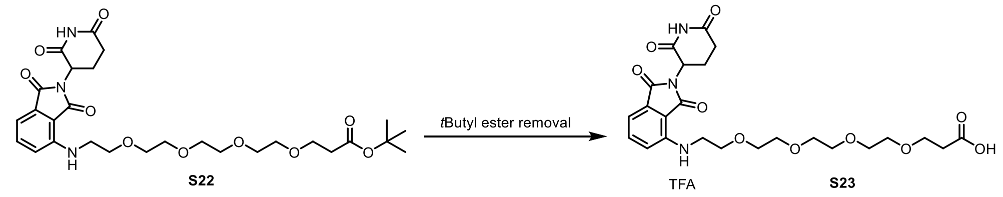

TFA (0.24 mL, 3.29 mmol, 100 equiv) was added to a solution of **S22**[28] (19 mg, 0.0329 mmol, 1.0 equiv) in DCM (5 mL) and the resulting reaction mixture was stirred for 30 min at 23 °C. After this time, the organic solvent and excess of TFA were removed under reduced pressure to afford the corresponding carboxylic acid trifluoroacetate salt **S23** (~0.0335 mmol) which was used in the next step without further purification.

Compound S1

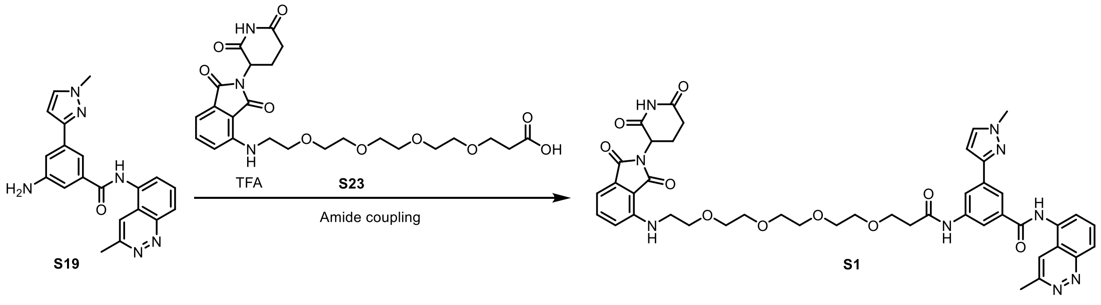

S1 was prepared from **S19**[5] (10 mg, 0.0279 mmol, 1.0 equiv) and **S23** (21 mg, 0.0335 mmol, 1.20 equiv) according to the general procedure 3A. The crude mixture was purified by preparative TLC (5% MeOH in DCM to 10% MeOH in DCM) followed by trituration with diethyl ether and pentane to afford **S1** (4 mg, 17%) as a yellow solid. ^{1}H NMR (400 MHz, Acetone-d_6) δ 9.93 (s, 1H), 9.43 (s, 1H), 8.43 (s, 1H), 8.38 – 8.29 (m, 2H), 8.23 (t, J = 1.6 Hz, 1H), 8.14 (s, 1H), 8.10 (dd, J = 7.4, 1.1 Hz, 1H), 7.90 (dd, J = 8.6, 7.4 Hz, 1H), 7.68 (d, J = 2.3 Hz, 1H), 7.53 (d, J = 8.3 Hz, 1H), 7.02 (d, J = 2.2 Hz, 1H), 6.93 (dd, J = 8.4, 2.2 Hz, 1H), 6.71 (d, J = 2.3 Hz, 1H), 6.31 (t, J = 5.3 Hz, 1H), 5.05 (dd, J = 12.6, 5.4 Hz, 1H), 3.97 (s, 3H), 3.86 (t, J = 5.9 Hz, 2H), 3.70 – 3.54 (m, 14H), 3.41 (q, J = 5.1 Hz, 2H), 2.91 (s, 3H), 2.83 – 2.77 (m, 3H), 2.69 (t, J = 5.9 Hz, 2H), 2.22 – 2.12 (m, 1H). ^{13}C NMR (126 MHz, Acetone-d_6) δ 171.79, 169.74, 169.40 167.77, 167.24, 166.36, 154.44, 153.47, 149.88, 149.51, 139.94, 139.91, 135.53, 134.97, 134.69, 133.25, 131.98, 129.21, 126.93, 126.11, 124.79, 122.44, 119.38, 119.09, 117.64, 117.55, 117.02, 115.82, 102.74, 70.32, 70.29, 70.21, 70.16, 68.95, 66.83, 49.03, 42.83, 38.33, 37.65, 31.10, 22.58, 21.34. 2 signals belonging to aliphatic carbons are not listed as they overlap with the solvent peak. HRMS (ESI): m/z calcd for $C_{44}H_{48}N_9O_{10}$ [M + H]$^+$: 862.35321, found 862.35264.

Compound S24

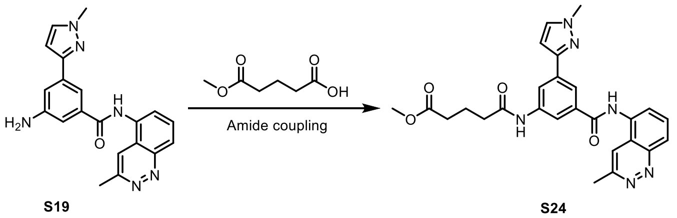

S19[5] (8.0 mg, 0.0223 mmol, 1.0 equiv) was dissolved in DMF (0.2 mL), to which HOBt·H_2O (2.6 mg, 0.0167 mmol, 0.75 equiv), EDC·HCl (13 mg, 0.0670 mmol, 3 equiv) and monomethyl glutarate (4.9 mg, 0.0335 mmol, 1.5 equiv) were added. The solution was stirred at 20 °C for 18 h. The organic solvent was removed under reduced pressure and the crude material was purified by preparative TLC (DCM with 5% MeOH), followed by a trituration in pentane, to afford **S24** (6.0 mg, 55 %) as an of off-white solid. ^{1}H NMR (500 MHz, DMSO-*d*$_6$) δ 10.68 (s, 1H), 10.18 (s, 1H), 8.38 – 8.32 (m, 2H), 8.18 – 8.13 (m, 2H), 8.03 (s, 1H), 7.95 – 7.88 (m, 2H), 7.79 (d, *J* = 2.2 Hz, 1H), 6.75 (d, *J* = 2.2 Hz, 1H), 3.92 (s, 3H), 3.61 (s, 3H), 2.89 (s, 3H), 2.44 – 2.37 (m, 4H), 1.87 (p, *J* = 7.4 Hz, 2H). ^{13}C NMR (126 MHz, DMSO-*d*$_6$) δ 173.05, 170.92, 166.43, 153.28, 149.29, 149.06, 139.78, 135.12, 134.12, 133.26, 132.57, 129.62, 127.09, 126.70, 122.47, 119.12, 118.55, 117.82, 117.70, 102.83, 51.28, 38.73, 35.27, 32.61, 21.70, 20.35. HRMS (ESI): m/z calcd for $C_{26}H_{27}N_6O_4$ $[M + H]^+$: 487.20883, found 487.20848.

Compound S25

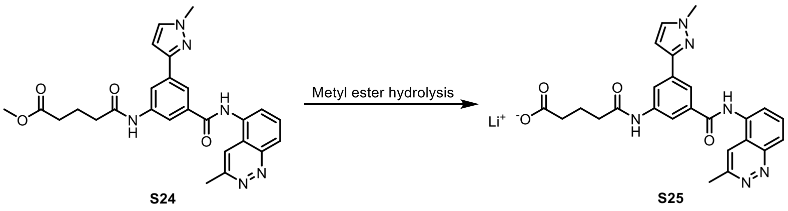

LiOH·H_2O (1.9 mg, 0.0455 mmol, 1.3 equiv) was added to the suspension of **S24** (17 mg, 0.0349 mmol, 1.0 equiv) in THF (0.18 mL) and H_2O (0.18 mL). The mixture was allowed to stir at 20 °C for 48 h. The organic solvent was removed under reduced pressure and the residue diluted with H_2O (0.3 mL). The solution was neutralised to pH 7 by a dropwise addition of HCl (0.02 M in H_2O) after which the solvent was removed under reduced pressure to afford the corresponding lithium carboxylate **S25** (~0.0347 mmol) as a pale yellow solid which was used in the next step without further purification.

Compound S27

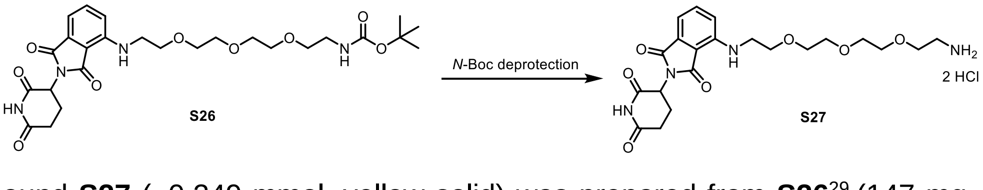

Compound **S27** (~0.249 mmol, yellow solid) was prepared from **S26**[29] (147 mg, 0.268 mmol, 1.0 equiv) according to the general procedure 2 and used in the next step without further purification.

Compound S2

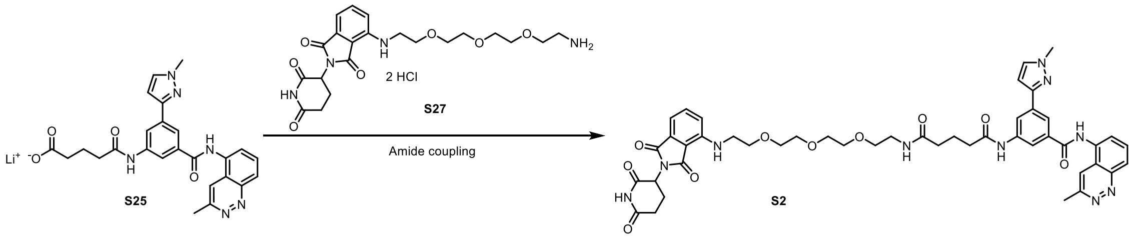

S25 (18 mg, 0.0347 mmol, 1.0 equiv) was dissolved in DMF (0.7 mL), to which HOBt·H_2O (5.3 mg, 0.047 mmol, 1.0 equiv) and EDC·HCl (13 mg, 0.0694 mmol, 2 equiv) were added. The solution was stirred at 20 °C for 45 min after which **S27** (20 mg, 0.0382 mmol, 1.1 equiv) and proton sponge (22 mg, 0.104 mmol, 3 equiv) were added in one portion. The reaction mixture was allowed to stir at 20 °C for 96 h. The organic solvent was then removed under reduced pressure and the crude material was purified by preparative TLC (DCM with 7 to 9% MeOH), followed by a trituration in pentane, to afford the **S2** (21 mg, 67 % over 2 steps from **S24**) as a bright yellow solid. ^{1}H NMR (500 MHz, CD_2Cl_2) δ 9.38 (s, 1H), 9.11 (s, 1H), 8.90 (s, 1H), 8.27 (d, *J* = 8.6 Hz, 1H), 8.15 (s, 1H), 8.09 (s, 1H), 8.02 (s, 1H), 7.93 (d, *J* = 7.4 Hz, 1H), 7.84 (s, 1H), 7.70 (t, *J* = 8.0 Hz, 1H), 7.42 – 7.35 (m, 2H), 6.92 (d, *J* = 7.1 Hz, 1H), 6.80 (d, *J* = 8.6 Hz, 1H), 6.57 (d, *J* = 2.3 Hz, 1H), 6.49 (t, *J* = 5.7 Hz, 1H), 6.37 (t, *J* = 5.5 Hz, 1H), 4.90 – 4.83 (m, 1H), 3.89 (s, 3H), 3.64 (t, *J* = 5.2 Hz, 2H), 3.63 – 3.54 (m, 8H), 3.49 (t, *J* = 5.2 Hz, 2H), 3.36 (dq, *J* = 10.6, 5.3 Hz, 4H), 2.84 (s, 3H), 2.77 – 2.62 (m, 3H), 2.37 (t, *J* = 7.1 Hz, 2H), 2.23 (t, *J* = 7.0 Hz, 2H), 2.09 – 2.01 (m, 1H), 1.95 (p, *J* = 7.0 Hz, 2H). ^{13}C NMR (126 MHz, CD_2Cl_2) δ 173.32, 172.24, 172.18, 169.93, 169.68, 167.94, 167.07, 154.14, 150.37, 149.74, 147.01, 139.74, 136.35, 135.58, 135.17, 132.71(2C), 132.16, 129.49, 127.77, 126.44, 122.73, 120.29, 120.11, 117.83, 117.19, 117.11, 111.62, 110.37, 103.53, 70.99, 70.90, 70.73, 70.55, 69.96, 69.58, 49.30, 42.65, 39.64, 39.39, 36.49, 35.38, 31.80, 23.10, 22.38, 22.06. HRMS (ESI): m/z calcd for $C_{46}H_{51}N_{10}O_{10}$ $[M + H]^+$: 903.37841, found 903.37784.

Compound S30

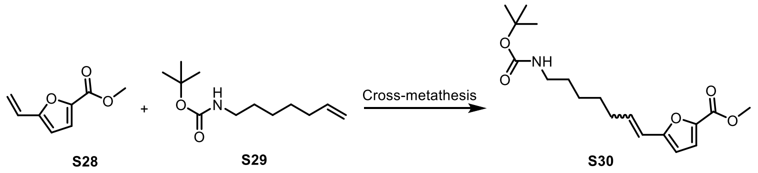

S28[30] (50 mg, 0.329 mmol, 1.0 equiv), **S29**[31] (140 mg, 0.658 mmol, 2 equiv) and Grubbs catalyst 2nd generation (14 mg, 0.016 mmol, 0.05 equiv) were flushed with nitrogen, dissolved in degassed dry DCM (0.6 mL) and the reaction mixture was stirred at 20 °C for 22 hours. After this time, the reaction mixture was diluted with diethyl ether and filtered over celite. The filtrate was concentrated under reduced pressure and the crude residue purified by flash chromatography (5 to 15% EtOAc in cyclohexane) to afford **S30** (82 mg, 74%) as a colourless oil. ^{1}H NMR (500 MHz, $CDCl_3$) δ 7.12 (d, *J* = 3.5 Hz, 1H), 6.48 – 6.38 (m, 1H), 6.27 – 6.18 (m, 2H), 4.51 (s, 1H), 3.88 (s, 3H), 3.11 (q, *J* = 6.7 Hz, 2H), 2.21 (qd, *J* = 7.3, 1.6 Hz, 2H), 1.48 (pd, *J* = 7.5, 2.8 Hz, 4H), 1.43 (s, 9H), 1.40 – 1.30 (m, 2H). ^{13}C NMR (126 MHz, $CDCl_3$) δ 159.37, 157.09, 156.11, 142.81, 134.95, 119.97, 118.21, 107.88, 79.20, 51.93, 40.64, 32.90, 30.09, 28.62, 28.56, 26.45. HRMS (ESI): m/z calcd for $C_{18}H_{28}NO_5$ $[M + H]^+$: 338.19620, found 338.19952

Compound S31

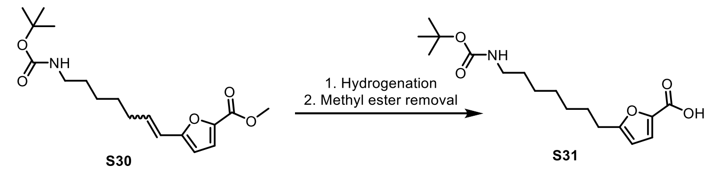

H_2 gas was bubbled into a suspension of **S30** (83 mg, 0.246 mmol, 1.0 equiv) and Pd/C (26 mg, 0.0123, 0.05 equiv) in MeOH (2.5 mL). After bubbling the content of two balloons, an atmosphere of H_2 was maintained at 20 °C for 3.5 h. After this time, the reaction mixture was flushed with nitrogen, filtered over celite and concentrated under reduced pressure to afford the corresponding alkane as a colourless oil (~0.162 mmol) which was used in the next step without further purification.The methyl ester obtained above (55 mg, 0.162 mmol) and LiOH·H_2O (14 mg, 0.325 mmol, 2 equiv) were dissolved in 2:1 THF/H_2O (1.2 mL) and the resulting reaction mixture was stirred at 20 °C for 14 hours. After this time, excess of organic solvent was removed under reduced pressure and the resulting mixture was cooled to 0 °C, acidified with aqueous HCl (0.1 M) until pH ~ 2, extracted with EtOAc, washed with brine and dried over anh. $MgSO_4$ to afford the corresponding carboxylic acid as colourless oil **S31** (~0.162 mmol) which was used in the next step without further purification.

Compound S3

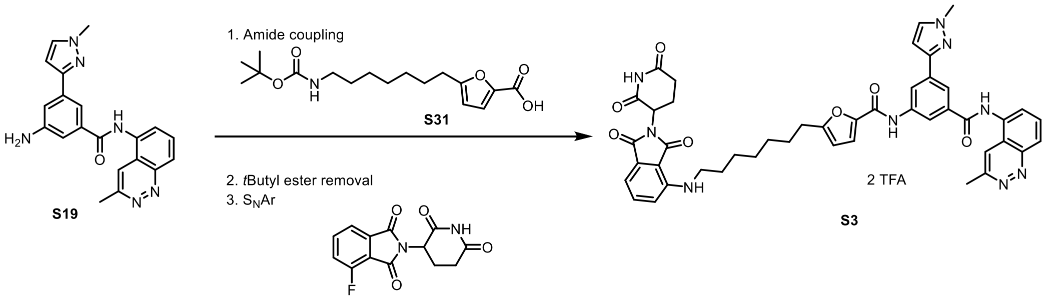

S19[5] (53 mg, 0.148 mmol, 1.0 equiv), **S31** (53 mg, 0.163 mmol, 1.1 equiv), HOBt·H_2O (22 mg, 0.163 mmol, 1.1 equiv), EDC·HCl (38 mg, 0.178 mmol, 1.2 equiv) and DIPEA (52 µL, 0.296 mmol, 2 equiv) were dissolved in DMF (1 mL) and the resulting reaction mixture was stirred at 20 ºC for 16 h. After this time, the reaction mixture was poured onto ice-cold water, filtered and the solid washed with water to afford the amide derivative as a yellow solid (~0.104 mmol) which was used in the following step without further purification. TFA (0.73 mL, 9.76 mmol, 100 equiv) was added to a solution of amide obtained above (65 mg, 0.0976 mmol, 1.0 equiv) in DCM (1 mL) and the resulting reaction mixture was stirred for 30 min at 20 ºC. After this time, the organic solvent and excess of TFA were removed under reduced pressure and the residue was taken up in 5% MeOH in DCM and filtered through amino-drerivatised silica to afford the corresponding amine (~0.0601 mmol) as a yellow solid which was used in the next step without further purification. Amine obtained above (15 mg, 0.0265 mmol, 1.0 equiv), 4-fluorothalidomide[32] (8.8 mg, 0.0318 mmol, 1.2 equiv) and DIPEA (14 uL, 0.0795 mmol, 3 equiv) were dissolved in DMSO (0.15 mL) and the resulting reaction mixture was stirred at 120 °C for 14. After this time, the solution was concentrated under reduced pressure and purified by preparative HPLC (20 to 95% ACN + 0.1% TFA in H_2O + 0.1% TFA) to afford **S3** (2.6 mg, 8% over 3 steps from **S19**) as a yellow solid. ^{1}H NMR (500 MHz, Methanol-*d4*) δ 8.42 – 8.36 (m, 2H), 8.33 (t, *J* = 1.8 Hz, 1H), 8.28 (t, *J* = 1.6 Hz, 1H), 8.19 (d, *J* = 0.9 Hz, 1H), 8.10 – 7.94 (m, 3H), 7.69 (d, *J* = 2.3 Hz, 1H), 7.56 – 7.44 (m, 2H), 7.26 (d, *J* = 3.5 Hz, 1H), 7.09 – 6.98 (m, 3H), 6.77 (d, *J* = 2.3 Hz, 1H), 6.35 – 6.30 (m,

1H), 5.10 – 5.00 (m, 1H), 3.99 (s, 3H), 2.97 (s, 3H), 2.87 – 2.68 (m, 6H), 2.13 – 2.09 (m, 1H), 1.80 (t, J = 7.4 Hz, 2H), 1.68 (d, J = 7.3 Hz, 2H), 1.47 (s, 7H). ^{13}C NMR (126 MHz, CD_2Cl_2) δ 171.64, 169.82, 169.33, 167.89, 166.61, 160.83, 160.12 (d, J = 39.8 Hz), 157.09, 152.65, 149.94, 148.34, 147.39, 145.80, 139.30, 136.43, 135.30, 133.85, 133.41, 133.14, 133.07, 132.78, 129.81, 126.56, 126.20, 125.43, 121.05, 120.82, 119.08, 117.58, 117.07, 111.34, 110.12, 108.93, 104.38, 49.25, 42.94, 39.27, 31.76, 29.39, 29.22, 29.19, 28.49, 28.06, 27.08, 23.04, 20.15. 6 signals belonging to TFA are not listed as they are overlapping with other signals or their itenstiy too low. HRMS (ESI): m/z calcd for $C_{45}H_{43}N_9O_7$ $[M + H]^+$: 822.33582, found 822.33497

Compound S32

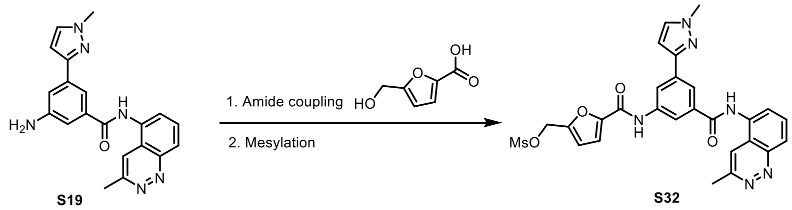

S19 S32

S19[5] (200 mg, 0.558 mmol, 1.0 equiv), 5-(hydroxymethyl)furan-2-carboxylic acid (87 mg, 0.614mmol, 1.1 equiv), HOBt·H_2O (83 mg, 0.614 mmol, 1.1 equiv) and EDC·HCl (235 mg, 1.23 mmol, 2.2 equiv) were dissolved in DMF (9 mL) and the resulting reaction mixture was stirred at 20 ºC for 14 h. After this time, the reaction mixture was poured onto ice-cold water, filtered and the solid washed with sat. aq. $NaHCO_3$ solution and water to achieve the corresponding amide product as a white solid (~0.558 mmol) which was used in the next step without further purification. Amide product obtained above (150 mg, 0.311 mmol, 1.0 equiv), methanesulfonyl chloride (50 µL, 0.645 mmol, 2.1 equiv) and triethylamine (54 µL, 0.389 mmol, 1.25 equiv) were dissolved in DMF (0.6 mL) and the resulting mixture was stirred at 20 °C for 14 hours. After this time, the reaction mixture was poured onto ice-cold water, the solid filtered and washed with 5% LiCl solution and water to achieve **S32** as a pale brown solid (~0.282 mmol) which was used in the next step without further purification.

Compound S34

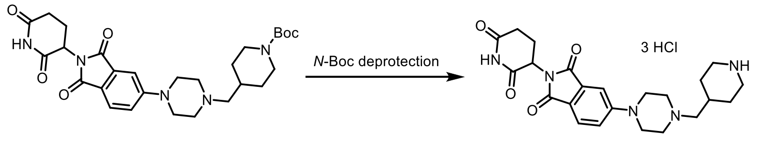

S33 S34

Compound **S34** (~0.424 mmol, yellow solid) was prepared from **S33**[33] (262 mg, 0.486 mmol, 1.0 equiv) according to a modified general procedure 2, by using a 1:1 mixture of DCM/MeOH (5 mL) and stirring the reaction at 4 °C for 10 hours, and used in the next step without further purification.

Compound S4

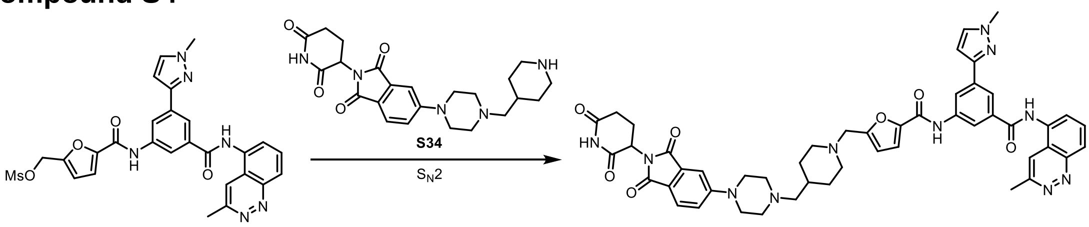

S32 S4

Mesylate **S32** (10 mg, 0.0178 mmol, 1.0 equiv), **S34** (12 mg, 0.0223 mmol, 1.25 equiv), potassium iodide (0.3 mg, 0.00178 mmol, 0.1 equiv) and $NaHCO_3$ (7.5 mg, 0.0890 mmol, 5 equiv) were dissolved in DMF (0.1 mL) and the reaction mixture stirred at 80 °C for 14 h. After this time, the solvent was removed under reduced pressure and the resulting crude purified by preparative TLC (10% MeOH in DCM) and triturated with pentane to achieve **S4** as a yellow solid (3 mg, 19% over 4 steps from **S19**). ^{1}H NMR (500 MHz, DMSO-d_6) δ 11.09 – 11.05 (m, 1H), 10.72 (s, 1H), 10.33 (s, 1H), 8.50 – 8.46 (m, 1H), 8.39 – 8.32 (m, 2H), 8.25 – 8.21 (m, 1H), 8.05 (s, 1H), 7.97 – 7.89 (m, 2H), 7.81 (d, *J* = 2.2 Hz, 1H), 7.67 (d, *J* = 8.5 Hz, 1H), 7.39 (d, *J* = 3.4 Hz, 1H), 7.32 (d, *J* = 2.2 Hz, 1H), 7.24 (dd, *J* = 8.7, 2.3 Hz, 1H), 6.78 (d, *J* = 2.2 Hz, 1H), 6.52 (d, *J* = 3.4 Hz, 1H), 5.06 (dd, *J* = 12.7, 5.5 Hz, 1H), 3.93 (s, 3H), 3.58 (s, 2H), 2.89 (s, 6H), 2.61 – 2.55 (m, 2H), 2.45 (t, *J* = 5.0 Hz, 3H), 2.24 – 2.13 (m, 2H), 2.06 – 1.98 (m, 3H), 1.70 (d, *J* = 12.5 Hz, 2H), 1.55 – 1.46 (m, 1H), 1.26 – 1.09 (m, 3H). ^{13}C NMR (126 MHz, DMSO- d_6) δ 172.82, 170.09, 167.57, 166.99, 166.39, 156.43, 155.76, 155.26, 153.32, 149.29, 149.08, 146.42, 139.28, 135.08, 134.13, 133.85, 133.26, 132.63, 129.65, 127.13, 126.74, 124.89, 122.49, 119.81, 119.69, 119.05, 118.28, 117.73, 115.67, 110.85, 107.85, 102.92, 63.90, 54.54, 52.88, 52.73, 48.76, 46.91, 38.77, 32.43, 30.98, 30.44, 22.18, 21.72. 1 signal belonging to an aromatic carbon (C-H) is not listed as it overlaps with another signal. HRMS (ESI): m/z calcd for $C_{49}H_{50}N_{11}O_7$ $[M + H]^+$: 904.38892, found 904.38906

Compound S10

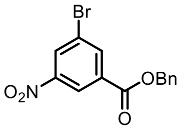

Benzyl bromide (1.07 mL, 8.94 mmol, 1.1 equiv) was added dropwise to a suspension of 3-bromo-5-nitrobenzoic acid (2.0 g, 8.13 mmol, 1.0 equiv) and Na_2CO_3 (1.53 g, 8.94 mmol, 1.1 equiv) in DMF (16 mL), while stirring at 25 °C for 16 h. After this time, the reaction mixture was transferred into a separatory funnel with H_2O and EtOAc, the aqueous phase was extracted three times with EtOAc, the combined organic phases washed three times with a sat. aq. NH_4Cl solution, dried over anh. $MgSO_4$, and the solvent was removed under reduced pressure. The crude was purified by column chromatography (1:4 to 1:1 DCM in hexane) to deliver **S10** (2.54 g, 93%) as a white solid. ^{1}H NMR (400 MHz, $CDCl_3$) δ 8.80 (dd, *J* = 2.1, 1.4, 1H), 8.55 (t, *J* = 2.0, 1H), 8.50 (t, *J* = 1.6, 1H), 7.49 – 7.34 (m, 5H), 5.42 (s, 2H). ^{13}C NMR (100 MHz, $CDCl_3$) δ 163.29, 148.90, 138.41, 135.04, 133.43, 130.66, 128.97, 128.95, 128.79, 123.40, 123.24, 68.21. mp = 85-86 ºC. HRMS (ESI): m/z: calcd. for $C_{14}H_{11}BrNO_4^+$ $[M + H]^+$: 335.97987, found: 335.98000.

Compound S11

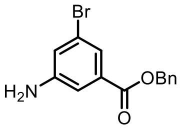

Zn dust (3.73 g, 57.1 mmol, 8.0 equiv) was added to a solution of **S10** (2.4 g, 7.11 mmol, 1.0 equiv) and NH_4Cl (7.64 g, 142.8 mmol, 20 equiv) in 6:1 MeOH:H_2O (240 mL). After vigorously stirring at 25 °C for 15 h, the crude mixture was filtered through a celite plug and washed with MeOH, and the solvent removed under reduced pressure. The crude was transferred to a separatory funnel with DCM and H_2O, and the aqueous phase was extracted three times with DCM. The combined organic phases were washed with brine,

dried over anh. $MgSO_4$, and the organic solvent removed under reduced pressure to deliver **S11** (2.06 g, 93%) as a yellow solid which did not require further purification. ^{1}H NMR (400 MHz, DMSO-d_6) δ 7.48 – 7.32 (m, 5H), 7.18 (dd, *J* = 2.2, 1.5, 1H), 7.14 (t, *J* = 1.6, 1H), 6.96 (t, *J* = 2.0, 1H), 5.75 (s, 2H), 5.30 (s, 2H). ^{13}C NMR (100 MHz, $CDCl_3$) δ 165.49, 147.82, 135.88, 132.73, 128.78, 128.52, 128.43, 123.07, 122.49, 121.90, 114.82, 67.15. mp = 88-89 ºC. HRMS (ESI): m/z: calcd. for $C_{14}H_{13}BrNO_2^+$ $[M + H]^+$: 306.01242, found: 306.01262.

Compound S12

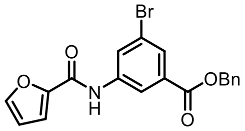

Furoyl chloride (0.64 mL, 6.52 mmol, 1.05 equiv) was added dropwise to a solution of **S11** (1.9 g, 6.21 mmol, 1.0 equiv) and Et_3N (1.29 mL, 9.32 mmol, 1.5 equiv) in DCM (21 mL). After stirring at 25 ºC for 4 h, the reaction mixture was transferred to a separatory funnel with DCM and sat. aq. Na_2CO_3 solution was added. The aqueous phase was extracted three times with DCM. The combined organic phases were washed with brine, dried over anh. $MgSO_4$ and the solvent was removed under reduced pressure. The resulting crude was purified by trituration with diethyl ether and the resulting solid was filtered through a glass frit to deliver **S12** (2.07 g, 68% over two steps from **S11**) as a white solid. ^{1}H NMR (400 MHz, DMSO-d$_6$) δ 10.57 (s, 1H), 8.42 – 8.36 (m, 2H), 7.98 (dd, *J* = 1.7, 0.8, 1H), 7.79 (t, *J* = 1.7, 1H), 7.52 – 7.33 (m, 6H), 6.73 (dd, *J* = 3.5, 1.7, 1H), 5.37 (s, 2H).^{13}C NMR (100 MHz, $CDCl_3$) δ 164.84, 156.13, 147.27, 144.75, 138.89, 135.65, 132.49, 128.83, 128.63, 128.55, 128.49, 127.16, 123.13, 119.46, 116.22, 112.99, 67.46. mp = 94-95 ºC. HRMS (ESI): m/z: calcd. for $C_{19}H_{14}BrNO_4Na^+$ $[M + Na]^+$: 421.99984, found: 422.00006.

Compound S13

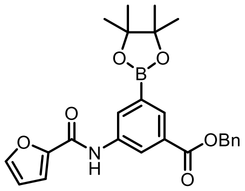

A solution of **S12** (864.5 mg, 2.16 mmol, 1.0 equiv), B_2pin_2 (1.72 g, 6.76 mmol, 3 equiv), KOAc (662.8 mg, 6.75 mmol, 3 equiv), Pd(dppf)Cl_2 (164 mg, 0.224 mmol, 0.1 equiv) in 1,4-dioxane (22 mL) was degassed by bubbling N_2 while stirring at 25 °C for 15 min. The reaction mixture was subsequently stirred at 85 °C for 23 h. After this time, the solvent was removed under reduced pressure, and the crude was purified by column chromatography (7:7 EtOAc : hexane, then 100% EtOAc), followed by trituration with pentane, to obtain **S13** (572 mg, 57%) as a white-brown foam. ^{1}H NMR (400 MHz, $CDCl_3$) δ 8.49 (dd, *J* = 2.4, 1.6, 1H), 8.27 (dd, *J* = 1.6, 1.0, 1H), 8.19 (dd, *J* = 2.4, 1.0, 1H), 8.13 (s, 1H), 7.51 (dd, *J* = 1.8, 0.8, 1H), 7.50 – 7.30 (m, 5H), 7.26 – 7.24 (m, 1H), 6.57 (dd, *J* = 3.5, 1.8, 1H), 5.39 (s, 2H), 1.35 (s, 12H). ^{13}C NMR (100 MHz, $CDCl_3$) δ 166.23, 156.17, 147.73, 144.47, 137.28, 136.18, 132.05, 130.82, 130.55, 128.72, 128.50, 128.37, 123.77, 115.68, 112.83, 84.40, 66.95, 25.02. HRMS (ESI): m/z: calcd. for $C_{25}H_{26}BNO_6Na^+$ $[M + Na]^+$: 470.175454, found: 470.17476.

Compound S14

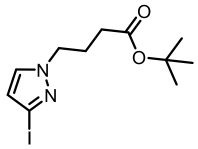

To a suspension of 3-iodo-1*H*-pyrazole (0.6 g, 3.09 mmol, 1.0 equiv) and Cs_2CO_3 (1.51 g, 4.64 mmol, 1.5 equiv) in DMF (6.2 mL), *t*-butyl 4-bromobutanoate (1.38 g, 6.17 mmol, 2.0 equiv) was added while stirring. After 17 h, the reaction mixture was transferred to a separatory funnel with EtOAc and H_2O. The aqueous phase was extracted three times with EtOAc and the combined organic phases were washed with sat. aq. NH_4Cl solution and brine, dried over $MgSO_4$ and the solvent was reduced *in vacuo*. The crude was purified by column chromatography (CH_2Cl_2 to 1:99 MeOH/CH_2Cl_2, then 2.5% to 10% EtOAc : petrol ether 40:60) to deliver the pure product **S14** (554 mg, 1.65 mmol, 53%) as a yellow oil. ^1H NMR (500 MHz, $CDCl_3$) δ 7.20 (d, *J* =2.3, 1H), 6.36 (d, *J* =2.3, 1H), 4.16 (t, *J*=6.8, 2H), 2.17 (td, *J* =6.9, 1.2, 2H), 2.09 (pd, *J* =6.9, 1.2, 2H), 1.41 (s, 9H). ^{13}C NMR (126 MHz, $CDCl_3$) δ 171.97, 131.55, 114.58, 94.17, 80.73, 51.69, 32.02, 28.17, 25.75. HRMS (ESI): m/z: calcd. for $C_{11}H_{18}O_2N_2I^+$ [M + H]$^+$: 337.04075 found: 337.04085.

Compound S15

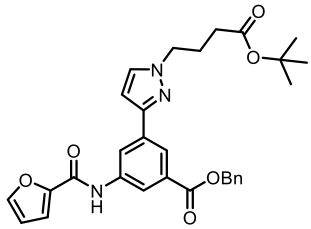

A solution of **S14** (1.28 g, 3.81 mmol, 1.0 equiv), **S13** (1.79 g, 4.0 mmol, 1.05 equiv), Cs_2CO_3 (3.72 g, 11.4 mmol, 3 equiv) and Pd(dppf)Cl_2 (139 mg, 0.191 mmol, 0.05 equiv) in a 9:1 1,4-dioxane/H_2O mixture was degassed by bubling N_2 while stirring at 25 °C for 15 min. The reaction mixture was subsequently heated at 85 °C for 21 h. After this time, the organic solvent was removed under reduced pressure, and the resulting crude mixture dissolved in DCM, filtered through a celite plug and the solvent was removed under reduced pressure. Purification by column chromatography (3:7 to 3:2 EtOAc in hexane, then 1% to 10% Et_2O in 1% MeOH in DCM) delivered **S15** (1.64 g, 82%) as a colorless foam. ^1H NMR (400 MHz, $CDCl_3$) δ 8.38 (t, *J* = 1.9, 1H), 8.27 (t, *J* = 1.8, 1H), 8.25 (t, *J* = 1.5, 1H), 8.20 (s, 1H), 7.53 (dd, *J* = 1.7, 0.8, 1H), 7.51 – 7.27 (m, 6H), 7.27 (d, *J* = 0.8, 1H), 6.62 (d, *J* = 2.2, 1H), 6.58 (dd, *J* = 3.5, 1.8, 1H), 5.40 (s, 2H), 4.22 (t, *J* = 6.5, 2H), 2.29 – 2.15 (m, 4H), 1.45 (s, 9H). ^{13}C NMR (100 MHz, $CDCl_3$) δ 172.11, 166.09, 156.13, 150.16, 147.60, 144.39, 137.98, 136.02, 135.06, 131.43, 130.93, 128.61, 128.31, 128.25, 122.90, 121.17, 119.83, 115.56, 112.70, 103.17, 80.65, 66.90, 51.37, 32.14, 28.12, 25.77. HRMS (ESI): m/z: calcd. for $C_{30}H_{32}N_3O_6$ [M + H]$^+$: 530.22856, found: 530.22889.

Compound S16

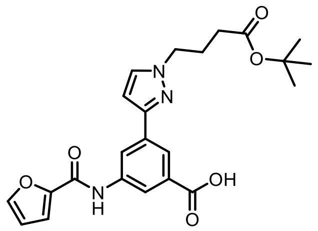

H_2 gas was bubbled to a solution of **S15** (529 mg, 1.0 mmol, 1.0 equiv) and Pd/C (79.9 mg, 15% wt) in MeOH (9 mL). After bubbling the content of three balloons, an atmosphere of H_2 was maintained for 21 h. The reaction mixture was subsequently flushed with nitrogen, filtered over a celite plug, washed with MeOH and the solvent was removed under reduced pressure. The resulting crude was purified by trituration with diethyl ether/pentane to obtain **S16** (349 mg, 79%) as a white foam. ^{1}H NMR (400 MHz, DMSO-*d$_6$*) δ 10.39 (s, 1H), 8.41 (t, *J* = 1.9, 1H), 8.36 (t, *J* = 1.8, 1H), 8.06 (t, *J* = 1.6, 1H), 7.96 (dd, *J* = 1.8, 0.8, 1H), 7.80 (d, *J* = 2.3, 1H), 7.41 (dd, *J* = 3.5, 0.8, 1H), 6.72 (dd, *J* = 3.5, 1.7, 1H), 6.70 (d, *J* = 2.3, 1H), 4.18 (t, *J* = 6.9, 2H), 2.21 (t, *J* = 7.4, 2H), 2.09 – 1.96 (m, 2H), 1.39 (s, 9H). ^{13}C NMR (100 MHz, Methanol-*d$_4$*) δ 173.91, 169.54, 159.03, 151.84, 148.87, 139.94, 135.87, 133.35, 133.00, 123.83, 123.16, 122.27, 116.48, 113.29, 104.10, 81.77, 52.16, 49.85, 33.09, 28.34, 26.97. HRMS (ESI): m/z: calcd. for $C_{23}H_{25}N_3O_6Na^+$ $[M + Na]^+$: 462.16356, found: 462.16380.

Compound S17

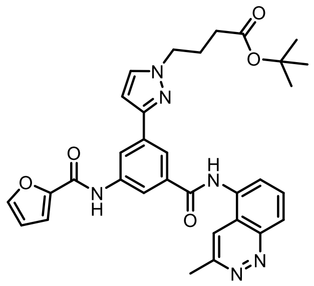

DIPEA (0.32 mL, 1.88 mmol, 3.0 equiv) was added dropwise to a solution of **S16** (275 mg, 0.62 mmol, 1.0 equiv) and HATU (262 mg, 0.69 mmol, 1.1 equiv) in NMP (6.3 mL). After stirring at 25 º C for 1 h, 3-methylcinnolin-5-amine (199 mg, 1.25 mmol, 2.0 equiv) was added, and the reaction mixture was heated at 80 °C for 96 h. Then, the reaction mixture was diluted with EtOAc and H_2O, and the aqueous phase was extracted three times with EtOAc. The combined organic phases were washed four times with a sat. aq. NH_4Cl solution, dried over anh. $MgSO_4$ and the solvent was removed under reduced pressure. Purification of the resulting crude by column chromatography (6% MeOH in DCM, then 8% MeOH : 25% toluene in DCM) delivered **S17** (107.5 mg, 30%) as a pale yellow foam.^{1}H NMR (400 MHz, Methanol-*d$_4$*) δ 8.40 – 8.34 (m, 3H), 8.30 (t, *J* = 1.6, 1H), 8.15 (d, *J* = 0.9, 1H), 8.02 (dd, *J* = 7.4, 1.1, 1H), 7.94 (dd, *J* = 8.6, 7.4, 1H), 7.79 (dd, *J* = 1.8, 0.8, 1H), 7.70 (d, *J* = 2.3, 1H), 7.33 (dd, *J* = 3.5, 0.8, 1H), 6.77 (d *J* = 2.3, 1H), 6.68 (dd, *J* = 3.5, 1.8, 1H), 4.27 (t, *J* = 6.8, 2H), 2.95 (d, *J* = 0.7, 3H), 2.35 – 2.22 (m, 2H), 2.22 – 2.12 (m, 2H), 1.44 (s, 9H). ^{13}C NMR (101 MHz, Methanol-*d$_4$*) δ 173.88, 169.54, 159.09, 155.45, 151.77, 150.72, 148.85, 146.99, 140.17, 136.50, 136.28, 134.14, 133.08, 131.31, 129.04, 128.18, 125.08, 122.65, 122.01, 120.68, 120.28, 116.66, 113.36, 104.24, 81.77, 52.24, 33.12, 28.34, 26.98, 21.88. HRMS (ESI): m/z: calcd. for $C_{32}H_{33}N_6O_5^+$ $[M + H]^+$: 581.25069, found: 581.25126.

Compound S18

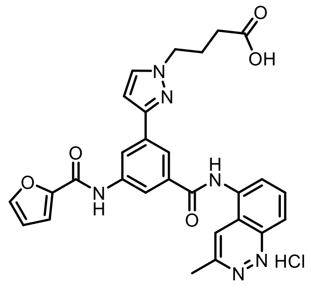

Compound **S18** (~0.105 mmol, yellow solid) was prepared from **S17** (85 mg, 0.146 mmol, 1.0 equiv) according to the general procedure 1 and used in the next step without further purification.

Compound S36

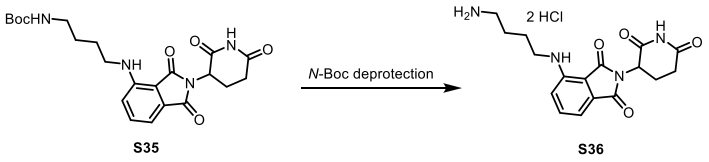

Compound **S36** (~0.314 mmol, yellow solid) was prepared from **S35**[34] (139.5 mg, 0.314 mmol, 1.0 equiv) according to the general procedure 2 and used in the next step without further purification.

Compound S5

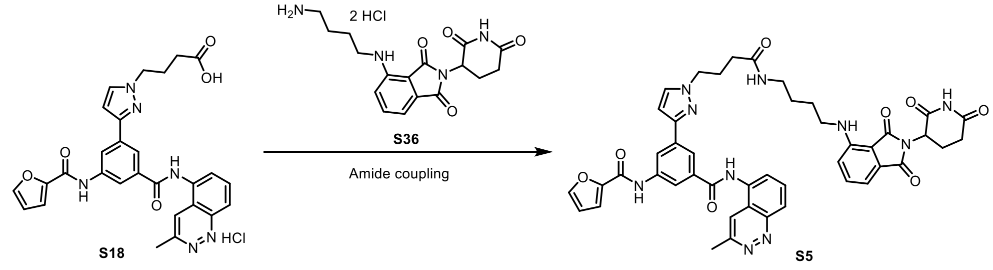

S5 was prepared from **S18** (12.9 mg, 0.023 mmol, 1.0 equiv) and **S36** (11.6 mg, 0.0277 mmol, 1.2 equiv) according to the general procedure 3B. The crude mixture was purified by preparative TLC (6% MeOH in DCM, then 50% EtOAc in DCM, then 6% MeOH in DCM) followed by trituration with diethyl ether to give **S5** (1.0 mg, 5%) as a yellow solid. ^{1}H NMR (400 MHz, Acetone-d_6) δ 8.57 (dd, J = 2.1, 1.5, 1H), 8.50 – 8.47 (m, 1H), 8.34 (dt, J = 8.6, 1.1, 1H), 8.30 (t, J = 1.6, 1H), 8.16 (s, 1H), 8.11 (dd, J = 7.5, 1.1, 1H), 7.90 (dd, J = 8.6, 7.4, 1H), 7.80 (dd, J = 1.7, 0.8, 1H), 7.73 (d, J = 2.3, 1H), 7.60 – 7.47 (m, 1H), 7.34 (dd, J = 3.5, 0.8, 1H), 7.27 (s, 1H), 7.06 (d, J = 8.6, 1H), 6.99 (dd, J = 7.1, 0.6, 1H), 6.74 (d, J = 2.3, 1H), 6.68 (dd, J = 3.5, 1.7, 1H), 6.39 (s, 1H), 5.04 (dd, J = 12.6, 5.4, 1H), 4.26 (t, J = 6.2, 2H), 3.37 (q, J = 6.7, 2H), 3.27 (q, J = 6.5, 2H), 2.93 (s, 3H), 2.81 – 2.65 (m, 3H), 2.22 – 2.15 (m, 5H), 1.71 (p, J = 7.0, 2H), 1.62 (q, J = 7.7, 7.2, 2H). ^{13}C NMR (126 MHz, Acetone-d_6) δ 172.67, 172.20, 170.30, 170.23, 168.28, 167.16, 157.25, 154.39, 150.72, 150.40, 148.90, 147.68, 146.21, 140.16, 136.93, 136.53, 135.98, 134.13, 133.59, 132.32, 130.11, 127.81, 126.95, 123.30, 120.90, 120.87,

119.31, 117.89, 117.62, 115.78, 113.11, 111.27, 110.77, 103.51, 52.11, 49.83, 42.68, 39.17, 33.26, 31.97, 27.35, 23.42, 22.23. 2 signals belonging to aliphatic carbons are not listed as they overlap with the solvent peak. HRMS (ESI): m/z: calcd. for $C_{45}H_{43}N_{10}O_8^+$ $[M + H]^+$: 851.32653, found: 851.32619.

Compound S38

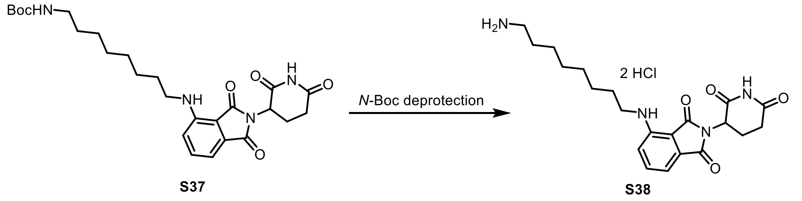

Compound **S38** (~0.047 mmol, yellow solid) was prepared from **S37**[35] (25 mg, 0.0499 mmol, 1.0 equiv) according to the general procedure 2 and used in the next step without further purification.

Compound S6

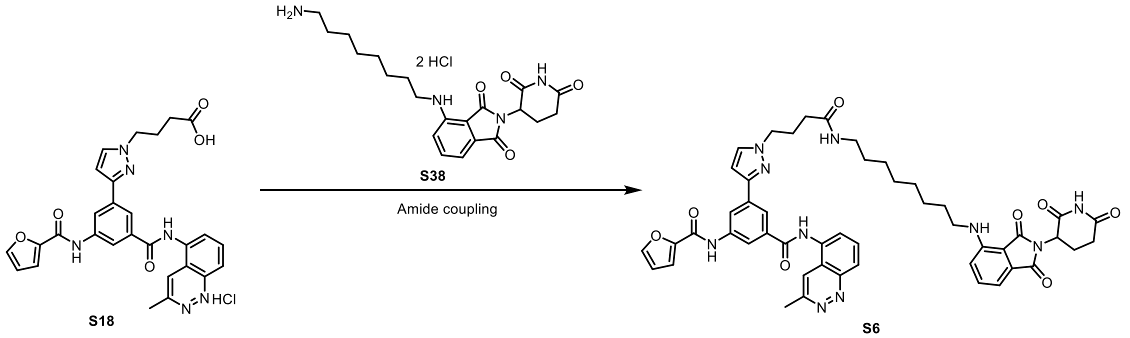

S6 was prepared from **S18** (15 mg, 0.027 mmol, 1.0 equiv) and **S38** (15.2 mg, 0.032 mmol, 1.2 equiv) according to the general procedure 3B. The crude mixture was purified by preparative TLC (6% MeOH in DCM) followed by trituration with diethyleter to give **S6** (8.0 mg, 33%) as a yellow solid. ^{1}H NMR (400 MHz, Acetone-d_6) δ 8.58 (t, *J* = 1.8, 1H), 8.51 (t, *J* = 1.9, 1H), 8.36 (d, *J* = 8.7, 1H), 8.32 (t, *J* = 1.6, 1H), 8.17 (s, 1H), 8.13 (dd, *J* = 7.4, 1.1, 1H), 7.92 (dd, *J* = 8.6, 7.4, 1H), 7.82 (d, *J* = 1.8, 1H), 7.75 (d, *J* = 2.3, 1H), 7.58 (dd, *J* = 8.5, 7.0, 1H), 7.35 (dd, *J* = 3.4, 0.8, 1H), 7.15 (s, 1H), 7.07 (d, *J* = 8.6, 1H), 7.02 (d, *J* = 7.0, 1H), 6.76 (d, *J* = 2.3, 1H), 6.70 (dd, *J* = 3.5, 1.7, 1H), 6.39 (s, 1H), 5.07 (dd, *J* = 12.6, 5.4, 1H), 4.35 – 4.16 (m, 2H), 3.35 (q, *J* = 6.7, 2H), 3.19 (q, *J* = 6.7, 2H), 2.92 (s, 3H), 2.84 – 2.69 (m, 3H), 2.28 – 2.17 (m, 4H), 1.67 (p, *J* = 7.1, 2H), 1.48 (d, *J* = 6.8, 2H), 1.41 (d, *J* = 7.6, 2H), 1.31 (d, *J* = 11.2, 6H). ^{13}C NMR (126 MHz, Acetone-d_6) δ 171.78, 171.13, 169.46, 169.33, 167.41, 166.25, 156.33, 153.48, 149.83, 149.52, 149.90, 148.03, 146.86, 145.32, 139.28, 136.05, 135.66, 135.10, 133.25, 132.73, 131.43, 129.23, 126.93, 126.04, 122.41, 119.97, 118.41, 116.98, 116.69, 114.88, 112.24, 110.35, 109.89, 102.60, 54.59, 51.26, 48.96, 42.08, 38.74, 32.38, 31.09, 29.70, 29.20, 29.05, 26.62, 26.57, 26.48, 22.54, 21.35. HRMS (ESI): m/z: calcd. for $C_{49}H_{51}N_{10}O_8^+$ $[M + H]^+$: 907.38858, found: 907.38830.

Compound S40

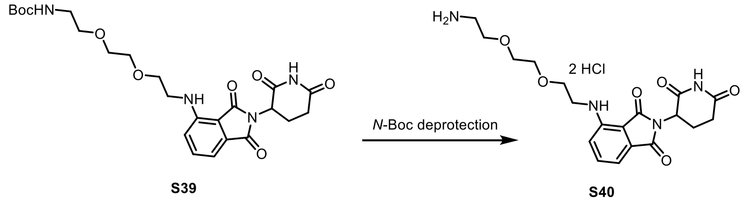

Compound **S40** (~0.470 mmol, yellow solid) was prepared from **S39**[36] (237 mg, 0.47 mmol) according to the general procedure 2 and used in the next step without further purification.

Compond S7

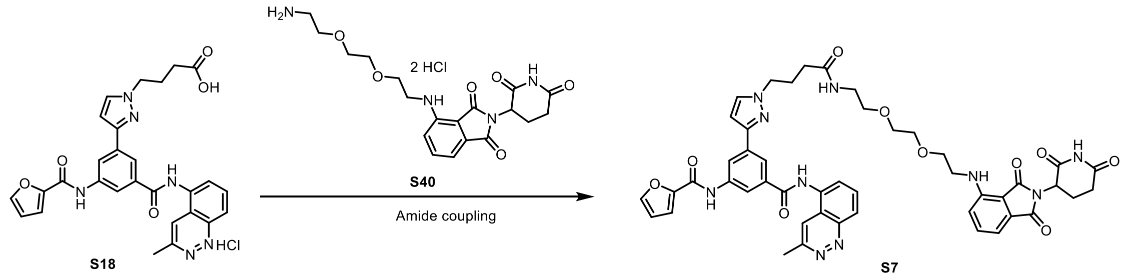

S7 was prepared from **S18** (15 mg, 0.027 mmol, 1.0 equiv) and **S40** (15.2 mg, 0.032 mmol, 1.2 equiv) according to the general procedure 3B. The crude was purified by preparative TLC (8% MeOH in DCM) followed by trituration with diethylether to obtain **S7** (10 mg, 41%) as a yellow solid.^{1}H NMR (400 MHz, Acetone-d$_6$) δ 8.54 (dd, *J* = 2.1, 1.5, 1H), 8.50 (dd, *J* = 2.1, 1.6, 1H), 8.34 (dt, *J* = 8.6, 1.1, 1H), 8.30 (t, *J* = 1.6, 1H), 8.16 (s, 1H), 8.10 (dd, *J* = 7.4, 1.1, 1H), 7.90 (dd, *J* = 8.6, 7.4, 1H), 7.81 (dd, *J* = 1.7, 0.8, 1H), 7.73 (d, *J* = 2.3, 1H), 7.59 – 7.49 (m, 1H), 7.34 (dd, *J* = 3.5, 0.8, 1H), 7.06 (d, *J* = 8.5, 1H), 7.00 (dd, *J* = 7.1, 0.6, 1H), 6.72 (d, *J* = 2.3, 1H), 6.68 (dd, *J* = 3.5, 1.7, 1H), 6.57 (t, *J* = 5.6, 1H), 5.06 (dd, *J* = 12.5, 5.4, 1H), 4.25 (t, *J* = 6.4, 2H), 3.68 (dd, *J* = 5.7, 4.9, 2H), 3.63 – 3.56 (m, 4H), 3.52 (t, *J* = 5.5, 2H), 3.47 (q, *J* = 5.6, 2H), 3.35 (t, *J* = 5.5, 2H), 2.80 – 2.67 (m, 2H), 2.27 – 2.12 (m, 6H). 1 signal belonging to the methyl group is not listed as it overlaps with the peak of water.^{13}C NMR (126 MHz, Acetone-d$_6$) δ 171.83, 171.43, 169.44, 169.40, 167.39, 166.25, 165.20, 159.41, 156.33, 153.50, 153.01, 149.83, 149.51, 146.75, 145.32, 139.27, 136.88, 136.02, 132.66, 131.54, 129.21, 126.97, 126.93, 126.12, 126.07, 119.98 119.88, 116.99, 116.92, 114.87, 112.23, 110.64, 110.14, 102.59, 70.30, 69.97, 69.60, 69.13, 51.13, 48.98, 41.97, 38.84, 32.29, 31.08, 26.45, 22.56, 21.35. HRMS (ESI): m/z: calcd. for $C_{47}H_{47}N_{10}O_{10}^+$ [M + H]$^+$: 911.34711, found: 911.34646.

Compound S8

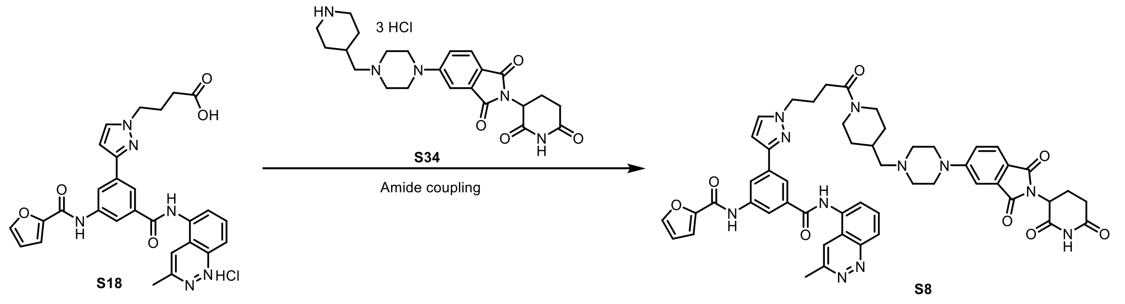

S8 was prepared from **S18** (12.6 mg, 0.0224 mmol, 1.0 equiv) and **S34** (14.8 mg, 0.0269 mmol, 1.2 equiv) according to the general procedure 3B. The crude was purified by preparative TLC (6% MeOH in DCM) followed by trituration with pentane to give **S8** (6.2 mg, 29%) as a yellow solid. ^{1}H NMR (400 MHz, Acetone-d_6) δ 8.72 – 8.66 (m, 1H), 8.65 – 8.60 (m, 1H), 8.35 – 8.29 (m, 2H), 8.22 (s, 1H), 8.13 (d, *J* = 7.3 Hz, 1H), 7.89 (dd, *J* = 8.6, 7.4 Hz, 1H), 7.80 (d, *J* = 1.5 Hz, 1H), 7.73 (d, *J* = 2.3 Hz, 1H), 7.64 (d, *J* = 8.5 Hz, 1H), 7.51 – 7.45 (m, 1H), 7.28 (d, *J* = 2.3 Hz, 1H), 7.22 (dd, *J* = 8.6, 2.3 Hz, 1H), 6.77 (d, *J* = 2.3 Hz, 1H), 6.68 (dd, *J* = 3.5, 1.8 Hz, 1H), 5.07 (dd, *J* = 12.6, 5.4 Hz, 1H), 4.52 (d, *J* = 13.3 Hz, 1H), 4.36 – 4.24 (m, 2H), 3.95 – 3.81 (m, 1H), 3.05 – 2.95 (m, 3H), 2.84 – 2.70 (m, 8H), 2.59 – 2.49 (m, 5H), 2.37 (t, *J* = 7.0 Hz, 2H), 2.25 – 2.12 (m, 5H), 1.78 (t, *J* = 13.0 Hz, 3H), 1.36 – 1.21 (m, 2H). ^{13}C NMR (126 MHz, Acetone-d_6) δ 171.77, 169.50, 169.29, 167.66, 167.05, 166.19, 156.39, 155.69, 153.46, 149.90, 149.53, 148.05, 145.34, 139.43, 135.49, 135.11, 134.39, 133.36, 131.46, 129.20, 126.82, 126.04, 124.67, 122.43, 120.08, 119.92, 118.41, 117.86, 117.15, 114.83, 112.16, 107.86, 102.63, 63.91, 52.97, 51.23, 49.17, 47.28, 45.20, 41.32, 33.35, 31.11, 30.40, 26.00, 22.54, 21.35. HRMS (ESI): m/z: calcd. for $C_{51}H_{52}N_{11}O_8^+$ [M + H]$^+$: 946.39948, found 946.40014.

Compound S42

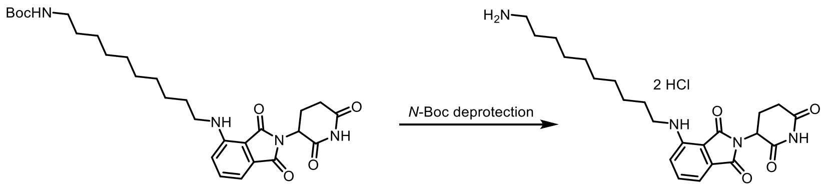

Compound **S42** (~0.470 mmol, yellow solid) was prepared from **S41**[37] (260 mg, 0.492 mmol, 1.0 equiv) according to the general procedure 2 and used in the next step without further purification.

Compound S9

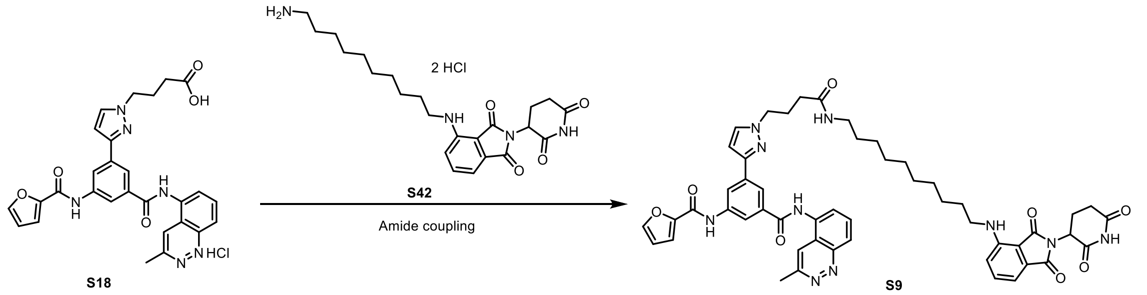

S9 was prepared from **S18** (10.3 mg, 0.0184 mmol, 1.0 equiv) and **S42** (11.25 mg, 0.0224 mmol, 1.23 equiv) according to the general procedure 3B. The crude was purified by preparative TLC (8% MeOH in 1:1 EtOAc:DCM, 4% MeOH in DCM) to give **S9** (1.16 mg, 7%) as a yellow solid. ^{1}H NMR (400 MHz, Acetone-d_6) δ 8.57 (t, *J* = 1.8, 1H), 8.51 (t, *J* = 1.9, 1H), 8.34 (dt, *J* = 8.5, 1.1, 1H), 8.30 (t, *J* = 1.6, 1H), 8.16 (s, 1H), 8.11 (d, *J* = 7.5, 1H), 7.90 (dd, *J* = 8.6, 7.4, 1H), 7.83 – 7.78 (m, 1H), 7.74 (d, *J* = 2.3, 1H), 7.56 (dd, *J* = 8.5, 7.1, 1H), 7.34 (dd, *J* = 3.5, 0.8, 1H), 7.06 (d, *J* = 8.6, 1H), 7.01 (d, *J* = 7.1, 1H), 6.75 (d, *J* = 2.3, 1H), 6.69 (dd, *J* = 3.5, 1.8, 1H), 6.39 (s, 1H), 5.06 (dd, *J* = 12.5, 5.4, 1H), 4.31 – 4.21 (m, 2H), 3.39 – 3.28 (m, 2H), 3.17 (t, *J* = 7.0, 2H), 2.90 (s, 3H), 2.82 – 2.67 (m, 2H), 2.19 (tt, *J* = 6.0, 3.0, 5H), 1.66 (p, *J* = 7.2, 2H), 1.46 (t, *J* = 7.1, 2H), 1.43 – 1.38 (m, 2H), 1.27 (s, 11H).^{13}C NMR (126 MHz, Acetone-d_6) δ 172.68, 172.03, 170.34,

170.21, 168.30, 167.13, 157.24, 154.37, 150.73, 150.41, 148.91, 147.76, 146.21, 140.19, 136.95, 136.53, 135.98, 134.15, 133.62, 132.31, 130.11, 127.80, 126.92, 123.30, 120.87, 119.31, 117.89, 117.57, 115.76, 113.11, 111.22, 110.77, 103.48, 120.85, 52.15, 49.85, 43.11, 42.99, 39.78, 39.65, 33.27, 31.98, 27.60, 27.54, 27.37, 23.42, 22.24. 4 signals belonging to aliphatic carbons are not listed as they overlap with the solvent peak. HRMS (ESI): m/z: calcd. for $C_{51}H_{55}N_{10}O_8^+$ $[M + H]^+$: 935.41989, found 935.42010.

Compound S44

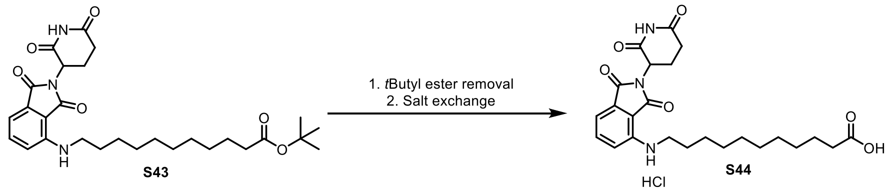

Compound **S44** (~0.043 mmol, yellow solid) was prepared from **S43**[38] (23 mg, 0.0440 mmol, 1.0 equiv) according to the general procedure 1 and used in the next step without further purification.

Compound 5 (dCE-2)

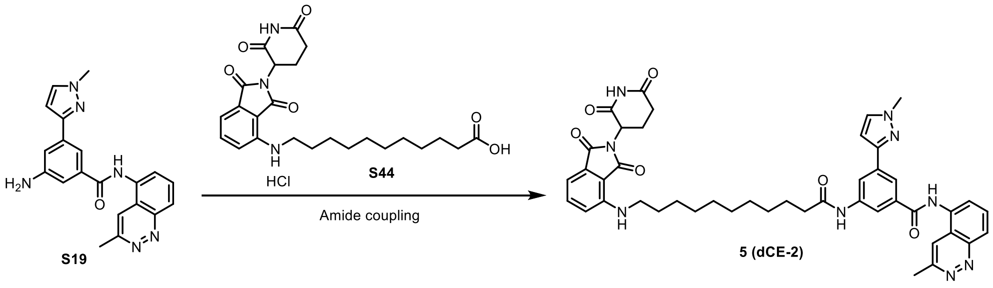

dCE-2 was prepared from aniline **S19**[5] (13 mg, 0.0363 mmol, 1.0 equiv) and carboxylic acid hydrochloric salt **S44** (22 mg, 0.0430 mmol, 1.20 equiv) according to the general procedure 3A. The crude mixture was purified by preparative TLC (8:1:1 DCM:EtOAc:MeOH) followed by trituration with diethyl ether and pentane to afford **dCE-2** (11 mg, 38%) as a yellow solid. ^{1}H NMR (400 MHz, DMSO-d_6) δ 11.10 (s, 1H), 10.69 (s, 1H), 10.14 (s, 1H), 8.36 – 8.33 (m, 2H), 8.17 – 8.13 (m, 2H), 8.02 (t, *J* = 0.9 Hz, 1H), 7.92 (s, 1H), 7.91 (d, *J* = 1.3 Hz, 1H), 7.79 (d, *J* = 2.2 Hz, 1H), 7.56 (dd, *J* = 8.5, 7.1 Hz, 1H), 7.07 (d, *J* = 8.7 Hz, 1H), 7.01 (d, *J* = 7.0 Hz, 1H), 6.75 (d, *J* = 2.3 Hz, 1H), 6.52 (t, *J* = 6.1 Hz, 1H), 5.04 (dd, *J* = 12.9, 5.4 Hz, 1H), 3.91 (s, 3H), 3.34 – 3.22 (m, 4H), 2.88 (s, 3H), 2.62 – 2.54 (m, 1H), 2.55 – 2.49 (m, 2H), 2.06 – 1.97 (m, 1H), 1.65 – 1.50 (m, 4H), 1.38 – 1.22 (m, 12H). ^{13}C NMR (126 MHz, DMSO-d_6) δ 173.29, 172.09, 170.57, 169.42, 167.77, 166.93, 153.76, 149.79, 149.54, 146.90, 140.37, 136.74, 135.59, 134.58, 133.74, 133.04, 132.66, 130.10, 127.57, 127.18, 122.95, 119.51, 118.96, 118.27, 118.17, 117.65, 110.83, 109.46, 103.30, 49.00, 42.29, 39.21, 36.90, 31.45, 29.45, 29.35, 29.25, 29.21, 29.15, 29.11, 26.79, 25.55, 22.62, 22.18. HRMS (ESI): m/z calcd for $C_{44}H_{48}N_9O_6$ $[M + H]^+$: 798.37221, found 798.37211.

Compound S46

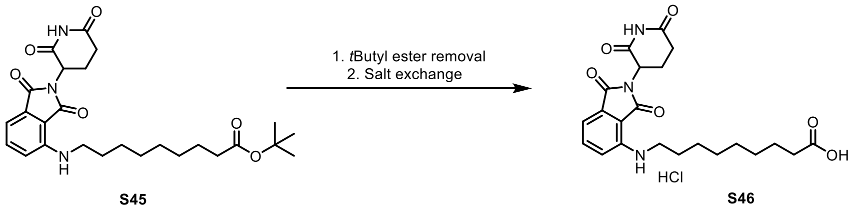

Compound **S46** (~0.277 mmol, yellow solid) was prepared from **S45**[38] (140 mg, 0.288 mmol, 1.0 equiv) according to the general procedure 1 and used in the next step without further purification.

Compound 6

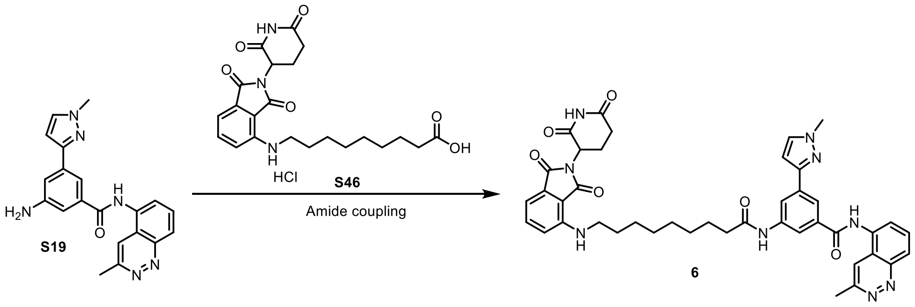

6 was prepared from **S19**[5] (10 mg, 0.0279 mmol, 1.0 equiv) and **S46** (14 mg, 0.0307 mmol, 1.1 equiv) according to the general procedure 3A. The crude mixture was purified twice by preparative TLC (5%MeOH in EtOAc and then 5% MeOH in DCM) followed by trituration with pentane to afford **6** (12 mg, 0.0156 mmol, 55 %) as a yellow solid. ^{1}H NMR (500 MHz, DMSO-d_6) δ 11.08 (s, 1H), 10.68 (s, 1H), 10.13 (s, 1H), 8.39 – 8.31 (m, 2H), 8.16 (dd, *J* = 4.3, 2.0 Hz, 2H), 8.02 (d, *J* = 2.8 Hz, 1H), 7.95 – 7.88 (m, 2H), 7.79 (d, *J* = 2.2 Hz, 1H), 7.56 (dd, *J* = 8.6, 7.0 Hz, 1H), 7.08 (d, *J* = 8.6 Hz, 1H), 7.00 (d, *J* = 7.0 Hz, 1H), 6.74 (d, *J* = 2.2 Hz, 1H), 6.52 (t, *J* = 5.9 Hz, 1H), 5.04 (dd, *J* = 12.8, 5.4 Hz, 1H), 3.92 (s, 3H), 3.31 – 3.25 (m, 2H), 2.88 (s, 4H), 2.66 – 2.51 (m, 2H), 2.34 (t, *J* = 7.4 Hz, 2H), 2.06 – 1.97 (m, 1H), 1.59 (dq, *J* = 21.4, 7.1 Hz, 4H), 1.38 – 1.28 (m, 8H). ^{13}C NMR (126 MHz, Methanol-d_4) δ 175.03, 174.63, 171.69, 170.79, 169.53, 169.31, 155.42, 151.68, 150.69, 148.28, 140.83, 137.20, 136.44, 136.08, 134.11, 133.88, 133.75, 131.30, 129.03, 128.16, 125.05, 121.57, 121.45, 120.27, 119.82, 117.95, 111.68, 110.92, 104.33, 50.16, 43.38, 39.09, 37.97, 32.19, 30.22, 30.20, 30.16, 30.08, 27.82, 26.71, 23.79, 21.87. HRMS (ESI): m/z calcd for $C_{42}H_{44}N_9O_6$ [M + H]$^+$: 770.34091, found 770.34089.

Compound S48

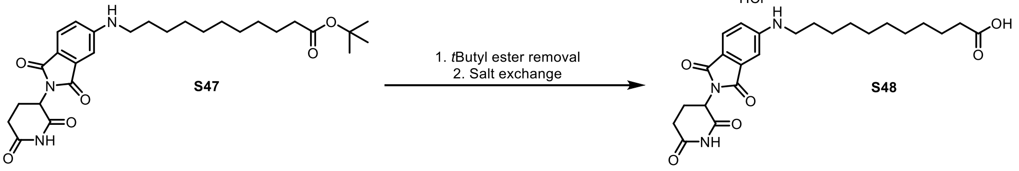

Compound **S48** (~0.180 mmol, yellow solid) was prepared from **S47**[39] (123 mg, 0.239 mmol, 1.0 equiv) according to the general procedure 1 and used in the next step without further purification.

Compound 7

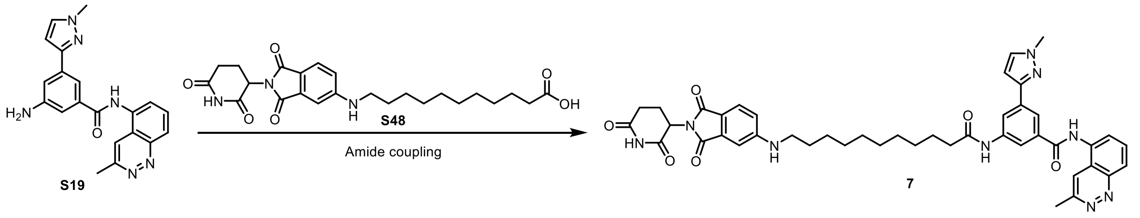

7 was prepared from aniline **S19**[5] (11 mg, 0.0307 mmol, 1.0 equiv) and carboxylic acid hydrochloric salt **S48** (18 mg, 0.0368 mmol, 1.2 equiv) according to a modified general procedure 3A, using DIPEA (16 µL, 0.0921 mmol, 3 equiv) instead of proton sponge. The crude mixture was purified by preparative TLC (63% DCM, 32% EtOAc, 5% MeOH) followed by trituration with MeOH and diethyl ether to afford **7** (12 mg, 50%) as a yellow solid. ^{1}H NMR (500 MHz, DMSO-*d*$_6$) δ 11.05 (s, 1H), 10.68 (s, 1H), 10.13 (s, 1H), 8.37 – 8.31 (m, 2H), 8.18 – 8.13 (m, 2H), 8.02 (s, 1H), 7.95 – 7.87 (m, 2H), 7.79 (d, *J* = 2.2 Hz, 1H), 7.54 (d, *J* = 8.4 Hz, 1H), 7.08 (t, *J* = 5.4 Hz, 1H), 6.93 (d, *J* = 2.1 Hz, 1H), 6.82 (dd, *J* = 8.5, 2.1 Hz, 1H), 6.74 (d, *J* = 2.2 Hz, 1H), 5.02 (dd, *J* = 12.7, 5.4 Hz, 1H), 3.92 (s, 3H), 3.13 (q, *J* = 6.5 Hz, 2H), 2.92 – 2.81 (m, 4H), 2.65 – 2.56 (m, 2H), 2.37 – 2.32 (m, 3H), 2.02 – 1.94 (m, 1H), 1.65 – 1.52 (m, 4H), 1.33 – 1.29 (m, 11H). ^{13}C NMR (126 MHz, DMSO-*d*$_6$) δ 172.83, 171.62, 170.19, 167.71, 167.16, 166.46, 154.46, 153.29, 149.32, 149.08, 139.90, 135.12, 134.20, 134.11, 133.29, 132.57, 129.63, 127.10, 126.71, 125.10, 122.49, 119.05, 118.49, 117.80, 117.72, 115.75, 102.84, 48.61, 42.47, 38.74, 36.43, 30.98, 29.02, 28.90, 28.81, 28.65, 28.24, 26.54, 25.09, 22.24, 21.71. 3 signals belonging to aromatic (2) and aliphatic (1) carbons are not listed as they overlap with other signals. HRMS (ESI): m/z calcd for $C_{44}H_{48}N_9O_6$ [M + H]$^+$: 798.37221, found 798.37156

Compound S50

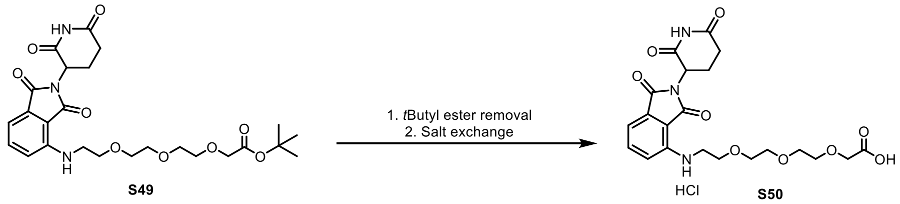

Compound **S50** (~0.121 mmol, yellow solid) was prepared from **S49**[40] (94 mg, 0.181 mmol, 1 equiv) according to the general procedure 1 and used in the next step without further purification.

Compound 8

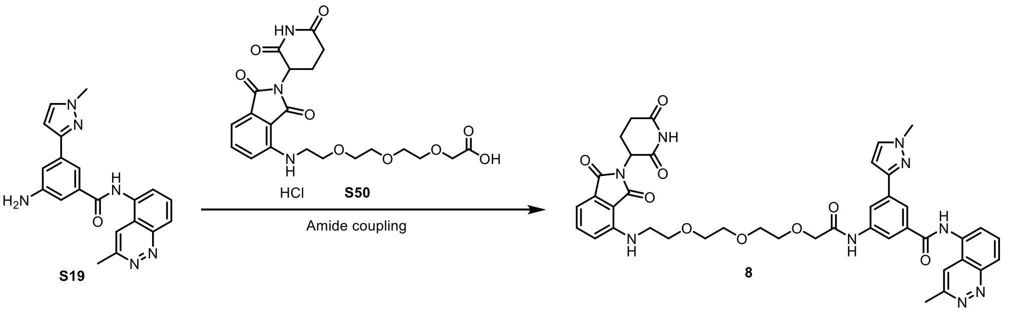

8 was prepared from aniline **S19**[5] (10 mg, 0.0287 mmol, 1.0 equiv) and carboxylic acid hydrochloric salt **S50** (22 mg, 0.0440 mmol, 1.58 equiv) according to a modified general procedure 3A, by using DIPEA (0.014 mL, 0.0837 mmol, 3.0 equiv) instead of proton sponge, and stirred at 60 ºC for 1 h. The crude mixture was purified by preparative TLC (10% MeOH in DCM in 2:1 DCM:EtOAc) followed by trituration with diethyl ether and pentane to afford **8** (9.1 mg, 40%) as a yellow solid. ^{1}H NMR (400 MHz, Acetone-*d$_6$*) δ 9.93 (s, 1H), 9.90 (s, 1H), 9.36 (s, 1H), 8.41 (t, J = 1.8 Hz, 1H), 8.32 (dt, J = 8.6, 1.1 Hz, 1H), 8.29 (t, J = 1.9 Hz, 1H), 8.20 (t, J = 1.6 Hz, 1H), 8.11 (t, J = 1.0 Hz, 1H), 8.07 (dd, J = 7.4, 1.1 Hz, 1H), 7.87 (dd, J = 8.6, 7.4 Hz, 1H), 7.65 (d, J = 2.3 Hz, 1H), 7.46 (dd, J = 8.6, 7.1 Hz, 1H), 6.95 (dd, J = 8.1, 4.5 Hz, 2H), 6.69 (d, J = 2.3 Hz, 1H), 6.50 (t, J = 5.7 Hz, 1H), 5.02 (dd, J = 12.6, 5.4 Hz, 1H), 4.12 (s, 2H), 3.95 (s, 3H), 3.85 – 3.74 (m, 6H), 3.71 (t, J = 5.3 Hz, 2H), 3.42 (q, J = 5.4 Hz, 2H), 3.31 (d, J = 5.2 Hz, 2H), 3.13 (q, J = 5.4 Hz, 1H), 2.93 (dd, J = 5.3, 3.3 Hz, 1H), 2.88 (s, 3H), 2.76 – 2.71 (m, 1H), 2.25 – 2.17 (m, 1H).^{13}C NMR (100 MHz, Acetone-*d$_6$*) δ 172.73, 170.38, 170.18, 169.65, 168.24, 167.10, 154.33, 150.66, 150.40, 147.62, 139.95, 136.73, 136.37, 135.89, 134.22, 133.49, 132.82, 130.06, 127.88, 127.02, 123.32, 120.62, 120.33, 118.83, 117.78, 117.65, 111.38, 111.01, 103.67, 72.08, 71.19, 71.09, 71.04, 70.94, 70.04, 49.90, 42.95, 39.23, 32.05, 23.41, 22.25. HRMS (ESI): m/z: calcd. For $C_{41}H_{42}O_9N_9^+$ $[M + H]^+$: 804.31055, found: 804.30916.

Compound S52

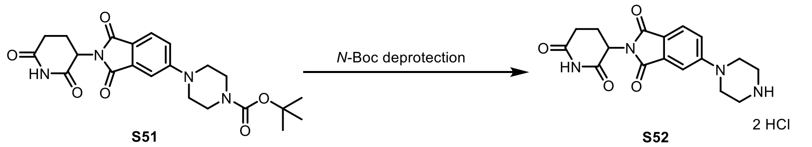

Compound **S52** (~1.07 mmol, yellow solid) was prepared from **S51**[41] (473 mg, 1.07 mmol, 1.0 equiv) according to the general procedure 2 and used in the next step without further purification.

Compound S53

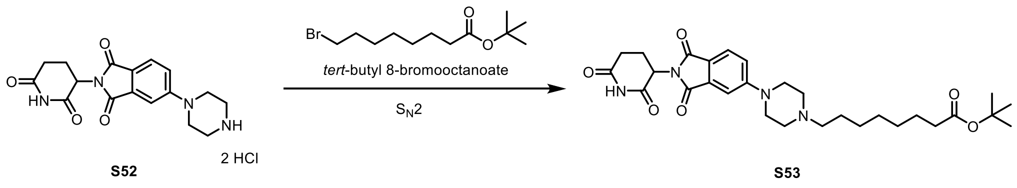

In a flame-dried flask, $NaHCO_3$ (50 mg, 0.600 mmol, 5 equiv) was dried under reduced pressure for 1 h. To it was added **S52** (50 mg, 0.120 mmol, 1 equiv) and anhydrous DMF (1.2 mL). Finally *tert*-Butyl 8-bromooctanoate (37 mg, 0.132 mmol, 1.1 equiv) was added with a pipette. The suspension was warmed up and stirred 2 h at 50 °C and 17 h at 80 °C. After which additional *tert*-butyl 8-bromooctanoate (17 mg, 0.0600 mmol, 0.5 equiv) was added and the reaction mixture was stirred at 80 °C for an additional 5 h. The organic solvent was then removed under reduced pressure and the crude material was purified by silica flash column chromatography (0 to 3% MeOH in DCM) to afford the title compound (45 mg, 69 % over 2 steps from **S51**) as a yellow solid. ^{1}H NMR (400 MHz, $CDCl_3$) δ 8.56 (s, 1H), 7.67 (d, *J* = 8.5 Hz, 1H), 7.27 (d, *J* = 2.4 Hz, 1H), 7.04 (dd, *J* = 8.5, 2.4 Hz, 1H), 4.97 – 4.87 (m, 1H), 3.42 (t, *J* = 5.1 Hz, 4H), 2.91 – 2.64 (m, 3H), 2.58 (t, *J* = 5.1 Hz, 4H), 2.42 – 2.33 (m, 2H), 2.19 (t, *J* = 7.5 Hz, 2H), 2.14 – 2.07 (m, 1H), 1.61 – 1.47 (m, 4H), 1.43 (s, 9H), 1.34 – 1.30 (m, 6H). ^{13}C NMR (101 MHz, $CDCl_3$) δ 173.39, 171.37, 168.57, 168.06, 167.38, 155.63, 134.37, 125.45, 119.53, 117.95, 108.72, 80.07,

58.62, 52.72, 49.25, 47.53, 35.67, 31.57, 29.30, 29.13, 28.25, 27.44, 26.77, 25.13, 22.87. HRMS (ESI): m/z calcd for $C_{29}H_{41}N_4O_6$ [M + H]$^+$: 541.30206, found 541.30172.

Compound S54

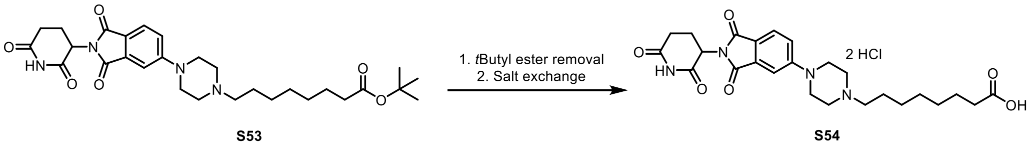

Compound **S54** (~0.0502 mmol, off white solid) was prepared from **S53** (40 mg, 0.0740 mmol, 1.0 equiv) according to a modified general procedure 1, by leaving the reaction to stir for 21 h, and used in the next step without further purification.

Compound 9

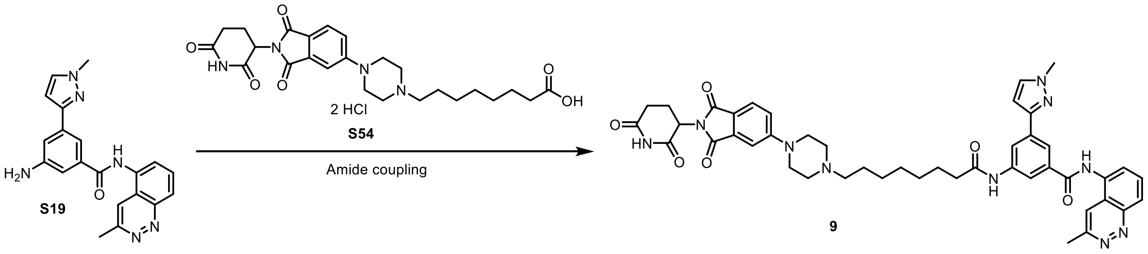

9 was prepared from aniline **S19**[5] (10 mg, 0.0279 mmol, 1 equiv) and carboxylic acid hydrochloric salt **S54** (17 mg, ~0.0307 mmol, 1.1 equiv) according to the general procedure 3A. The crude mixture was purified by two subsequent preparative TLCs (Firstly: DCM with 20% EtOAc, 5% MeOH and 0 to 1% Et_3N; secondly: pretreated TLC in DCM with 2% Et_3N, eluent for purification DCM with 5% MeOH and 1% Et_3N). This was followed with a purification by reversed-phase HPLC using a Shimadzu Nexera system, equipped with a an Agilent Zorbax 300SB-C18 semi-preparative column (9.4 x 250 mm 5-micron) using 20 to 50% acetonitrile in water buffered with 0.1% formic acid as eluent (8 mL/min) to afford **9** (9 mg, 39 %) as a yellow-brown solid. ^{1}H NMR (500 MHz, Acetone-d_6) δ 10.00 (s, 1H), 9.86 (s, 1H), 9.38 (s, 1H), 8.41 (s, 1H), 8.36 – 8.31 (m, 2H), 8.21 (s, 1H), 8.12 (s, 1H), 8.08 (d, J = 7.2 Hz, 1H), 7.89 (t, J = 8.0 Hz, 1H), 7.67 (d, J = 2.3 Hz, 1H), 7.63 (d, J = 8.5 Hz, 1H), 7.28 (d, J = 2.4 Hz, 1H), 7.22 (dd, J = 8.4, 2.4 Hz, 1H), 6.69 (d, J = 2.3 Hz, 1H), 5.06 (dd, J = 12.7, 5.4 Hz, 1H), 3.95 (s, 3H), 3.46 (t, J = 5.1 Hz, 4H), 2.56 (t, J = 5.1 Hz, 4H), 2.46 (t, J = 7.3 Hz, 2H), 2.37 (t, J = 7.3 Hz, 2H), 2.19 – 2.16 (m, 1H), 1.79 – 1.72 (m, 2H), 1.57 – 1.50 (m, 2H), 1.45 – 1.38 (m, 6H); 6 H are not listed as they overlap with the water signal; ^{13}C NMR (126 MHz, Acetone-d_6) δ 172.7, 172.4, 170.2, 168.5, 167.9, 167.2, 156.6, 154.3, 150.8, 150.4, 141.2, 136.5, 135.9, 135.3, 134.3, 132.8, 130.1, 127.9, 127.1, 125.5, 123.4, 120.2, 120.0, 119.8, 118.7, 118.5, 117.8, 108.7, 103.6, 59.0, 53.6 (2C), 50.1, 48.3 (2C), 39.2, 37.7, 32.0, 27.9, 27.4, 26.1, 23.4, 22.2; 2 signals belonging to aliphatic carbons are not listed as they overlap with the solvent peak. HRMS (ESI): m/z calcd for $C_{45}H_{49}N_{10}O_6$ [M + H]$^+$: 825.38311, found 825.38459.

Compound S56

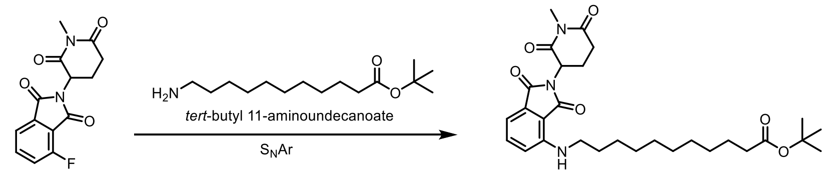

S55[42] (102 mg, 0.3530 mmol, 1.0 equiv) and *tert*-butyl 11-aminoundecanoate (100 mg, 0.3880 mmol, 1.10 equiv) were dissolved in DMSO (1.7 mL) and to this solution was added DIPEA (0.12 mL, 0.7060 mmol, 2.0 equiv). The resulting reaction mixture was heated at 130 ºC for 15 h. After this time, DMSO was removed under reduced pressure and the resulting crude mixture was purified by column chromatography (1% MeOH in DCM to 1.5% MeOH in DCM) to afford **S56** (100 mg, 54%) as a yellow foam. ^{1}H NMR (400 MHz, Methanol-d_4) δ 7.57 (dd, *J* = 8.6, 7.1 Hz, 1H), 7.09 – 7.03 (m, 2H), 5.10 (dd, *J* = 12.9, 5.4 Hz, 1H), 3.40 – 3.33 (m, 2H), 3.17 (s, 3H), 2.96 – 2.87 (m, 2H), 2.78 – 2.64 (m, 1H), 2.22 (t, *J* = 7.4 Hz, 2H), 2.16 – 2.06 (m, 1H), 1.69 (p, *J* = 7.1 Hz, 2H), 1.62 – 1.52 (m, 2H), 1.46 (s, 9H), 1.43 – 1.30 (m, 12H). ^{13}C NMR (126 MHz, Methanol-d_4) δ 173.78, 172.28, 170.06, 169.43, 167.96, 146.95, 135.84, 132.53, 116.59, 110.30, 109.56, 79.92, 49.42, 42.00, 35.04, 31.09, 29.15, 29.05, 28.98, 28.91, 28.89, 28.68, 26.95, 26.54, 25.92, 24.81, 21.65. HRMS (ESI): m/z calcd for $C_{29}H_{42}N_3O_6$ [M + H]$^+$: 528.30736, found 528.30771.

Compound S57

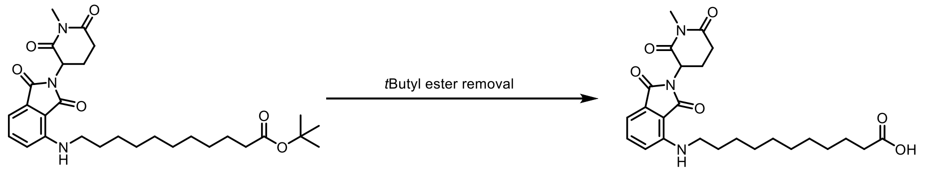

TFA (0.20 mL, 2.70 mmol, 100 equiv) was added to a solution of **S56** (14 mg, 0.0270 mmol, 1.0 equiv) in DCM (5 mL) and the resulting reaction mixture was stirred at 23 ºC for 30 min. After this time, the organic solvent and excess of TFA were removed under reduced pressure in rotavapor to afford the corresponding carboxylic acid trifluoroacetate salt **S57** (~0.0270 mmol) which was used in the next step without further purification.

Compound 10

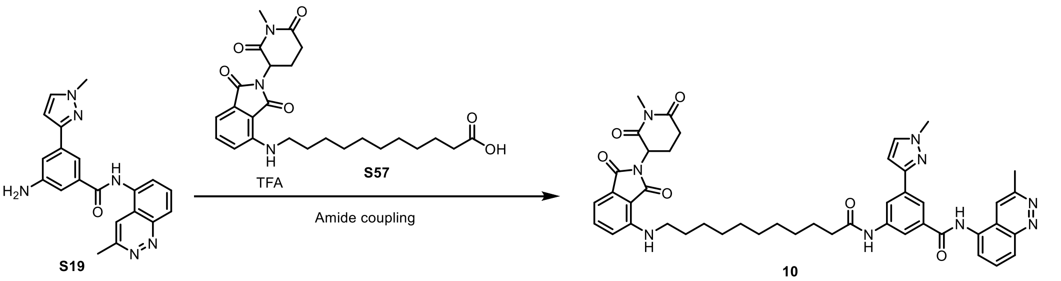

10 was prepared from aniline **S19**[5] (8.0 mg, 0.0220 mmol, 1.0 equiv) and carboxylic acid trifluoroacetate salt **S57** (16 mg, 0.0268 mmol, 1.20 equiv) according to the general procedure 3A. The crude mixture was purified by preparative TLC (9:0.5:0.5 DCM:EtOAc:MeOH) followed by trituration with diethyl ether and pentane to afford **10**

(2.8 mg, 16%) as a yellow solid. ^{1}H NMR (400 MHz, Acetone-d_6) δ 8.38 (t, *J* = 1.8 Hz, 1H), 8.36 – 8.31 (m, 2H), 8.20 (t, *J* = 1.6 Hz, 1H), 8.17 – 8.11 (m, 1H), 8.08 (dd, *J* = 7.5, 1.2 Hz, 1H), 7.93 – 7.85 (m, 1H), 7.67 (d, *J* = 2.3 Hz, 1H), 7.57 (dd, *J* = 8.5, 7.1 Hz, 1H), 7.08 (d, *J* = 8.5 Hz, 1H), 7.01 (d, *J* = 7.1 Hz, 1H), 6.69 (d, *J* = 2.3 Hz, 1H), 6.40 (t, *J* = 6.1 Hz, 1H), 5.07 (dd, *J* = 13.0, 5.3 Hz, 1H), 3.95 (s, 3H), 3.36 (q, *J* = 6.7 Hz, 2H), 3.08 (s, 3H), 2.90 (s, 3H), 2.80 – 2.64 (m, 1H), 2.45 (t, *J* = 7.4 Hz, 2H), 2.22 – 2.12 (m, 3H), 2.06 – 1.94 (m, 1H), 1.79 – 1.62 (m, 4H), 1.48 – 1.25 (m, 11H). ^{13}C NMR (126 MHz, Acetone-d_6) δ 171.53, 171.38, 169.54, 169.47, 167.44, 166.30, 153.46, 149.92, 149.53, 146.90, 140.15, 136.07, 135.53, 134.95, 133.32, 132.75, 131.94, 129.20, 126.90, 126.12, 122.47, 119.18, 118.88, 118.38, 117.51, 117.05, 116.69, 110.34, 102.69, 49.56, 42.12, 38.31, 36.82, 31.38, 28.32, 27.65, 26.69, 26.16, 25.32, 21.79, 21.33. 4 signals belonging to aliphatic carbons are not listed as they overlap with other signals the region between 28-29 ppm. HRMS (ESI): m/z calcd for $C_{45}H_{50}N_9O_6$ [M + H]$^+$: 812.38786, found 812.38771.

4. ^{1}H and ^{13}C NMR Spectra

Compound 3. ^{1}HNMR (500 MHz, DMSO-d_6) and ^{13}C NMR (126 MHz, DMSO-d_6).

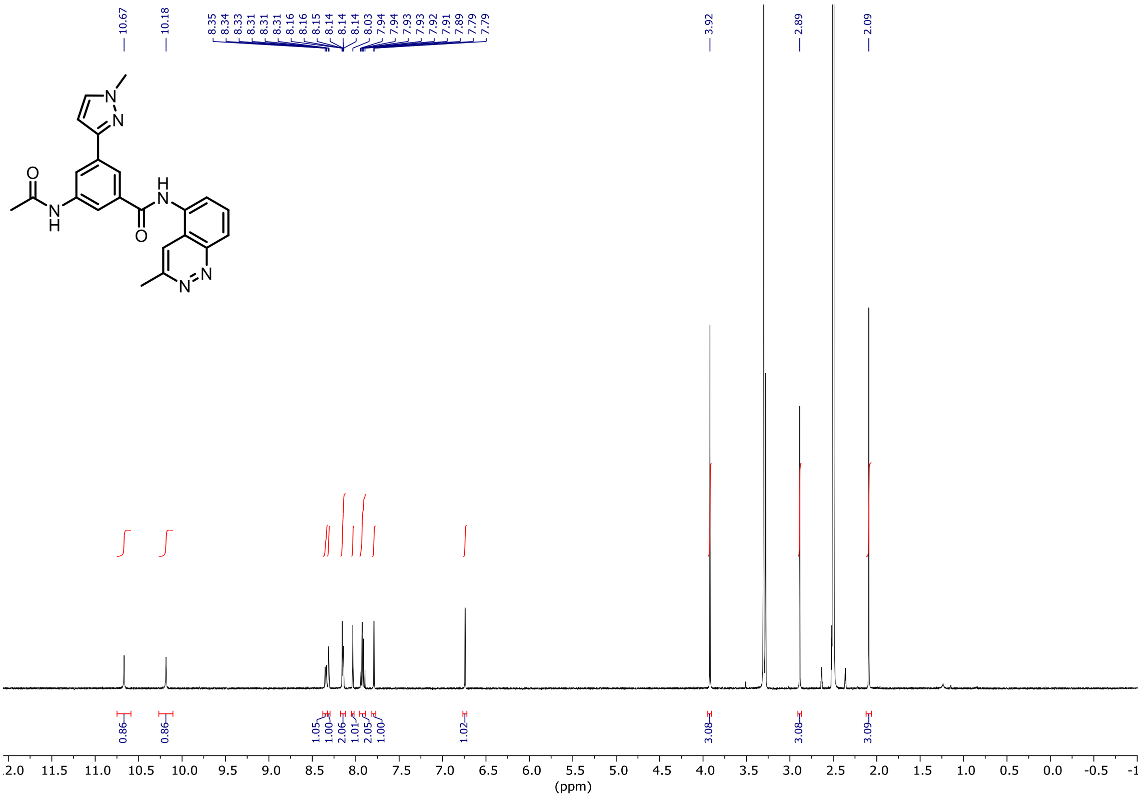

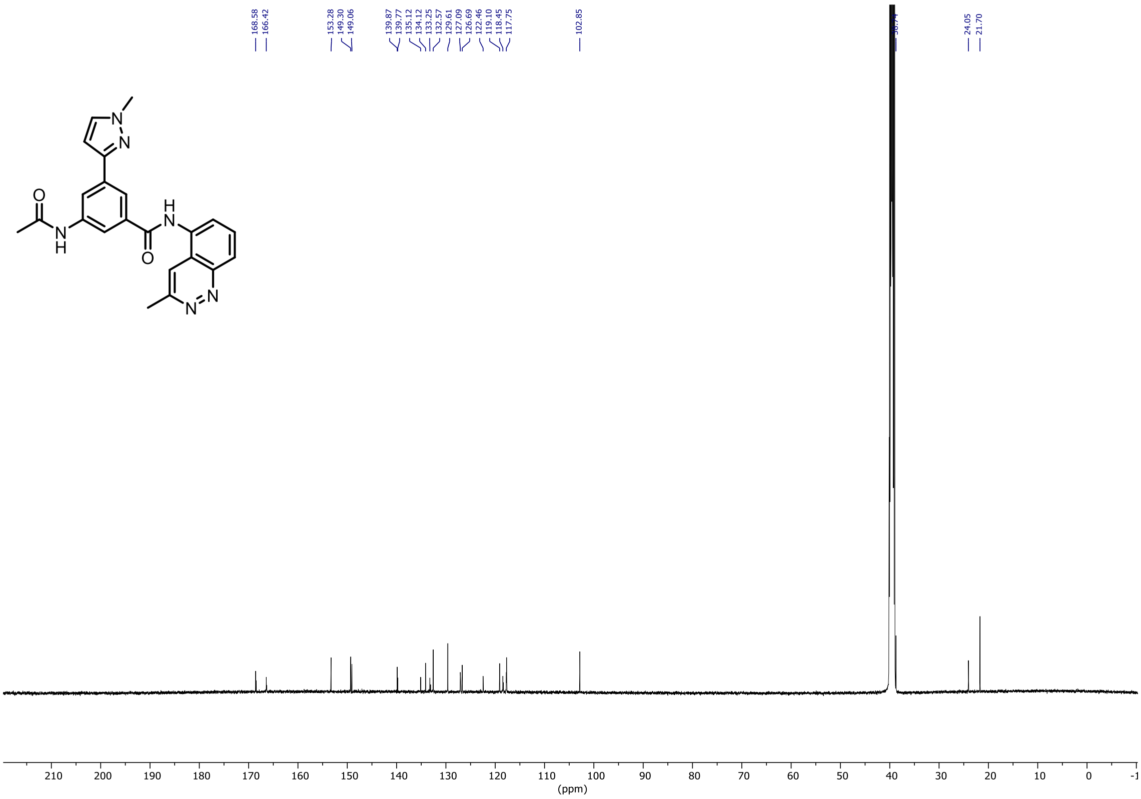

Compound 4. ^{1}H NMR (400 MHz, Methanol-d_4) and ^{13}C NMR (100 MHz, DMSO-d_6).

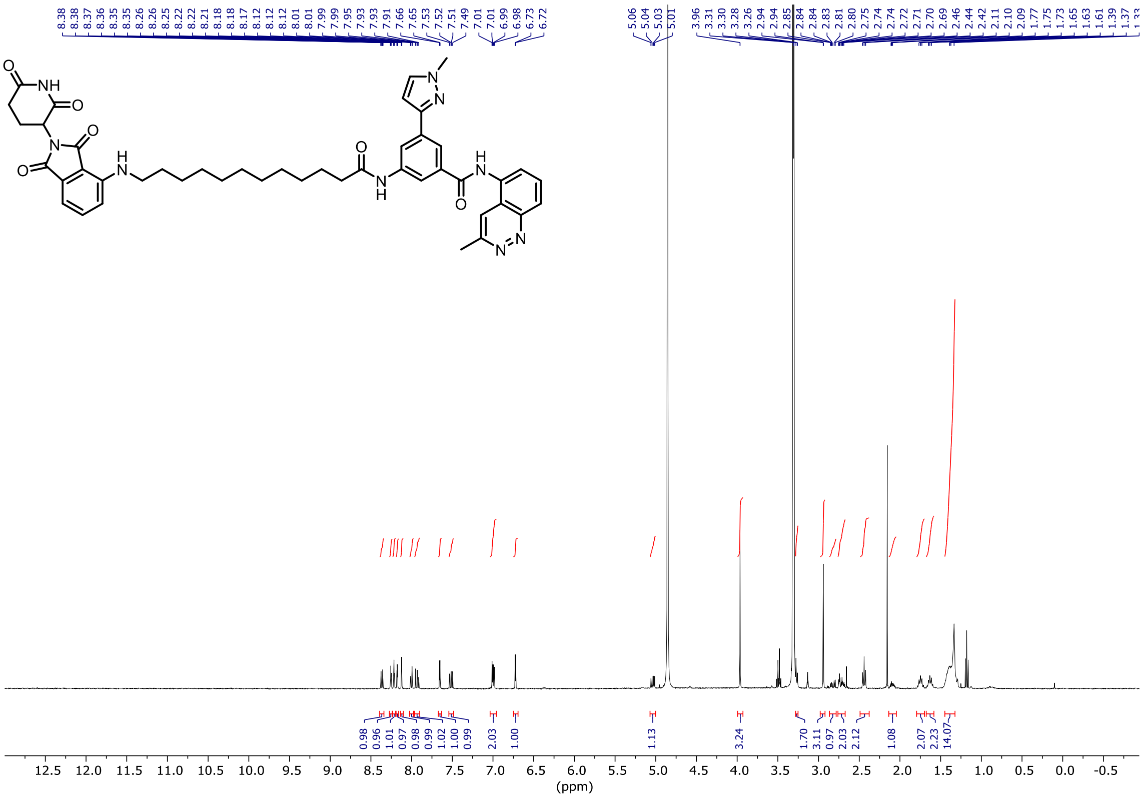

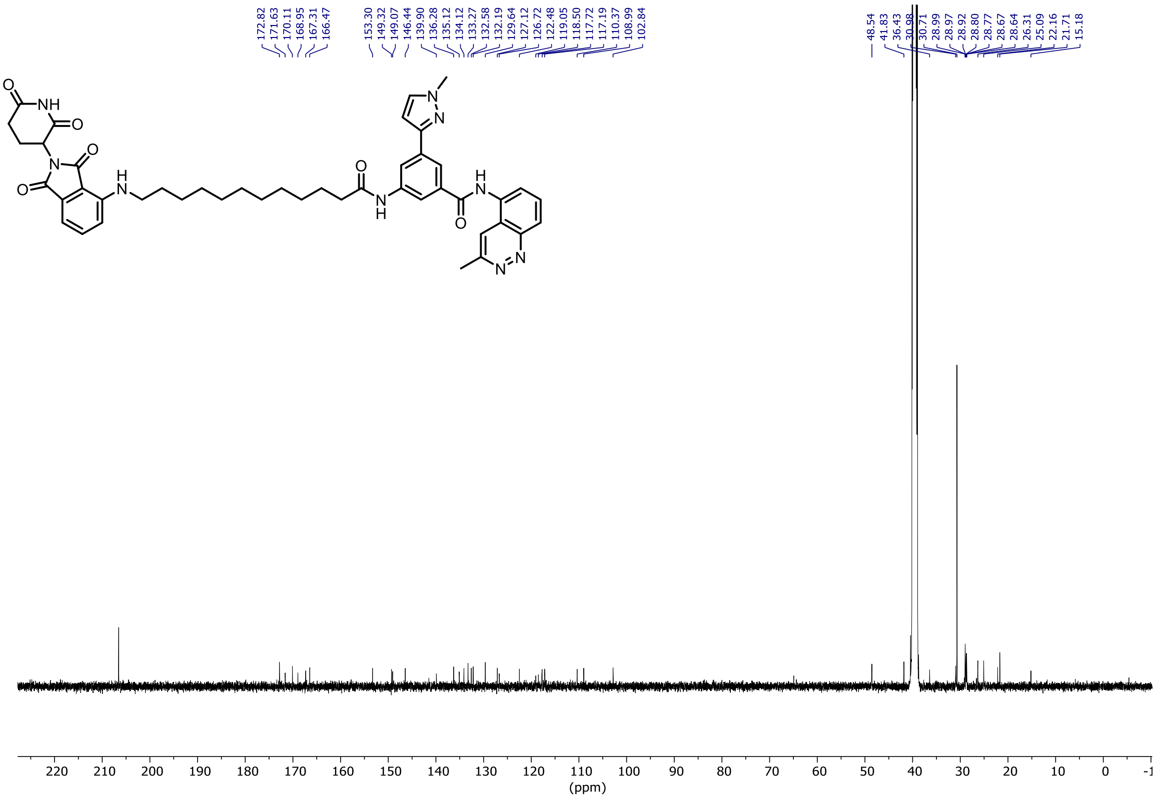

Compound S1. ^{1}HNMR (400 MHz, Acetone-d_6) and ^{13}C NMR (126 MHz, Acetone-d_6).

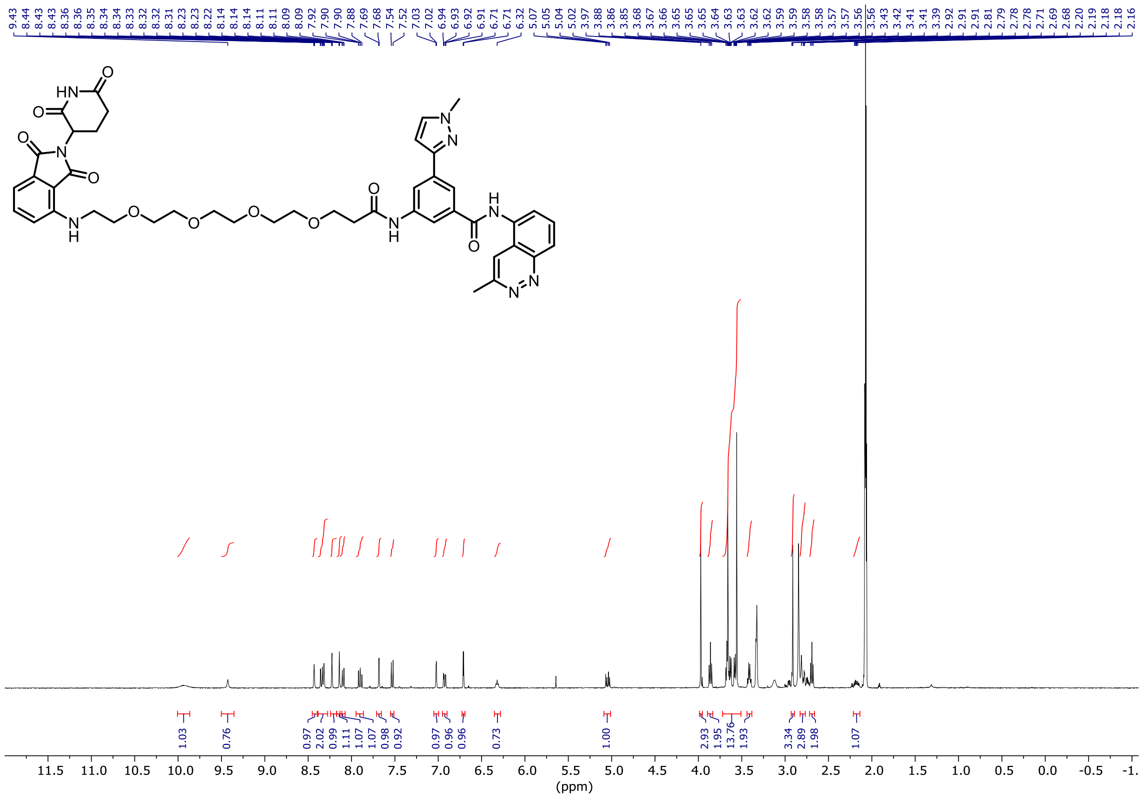

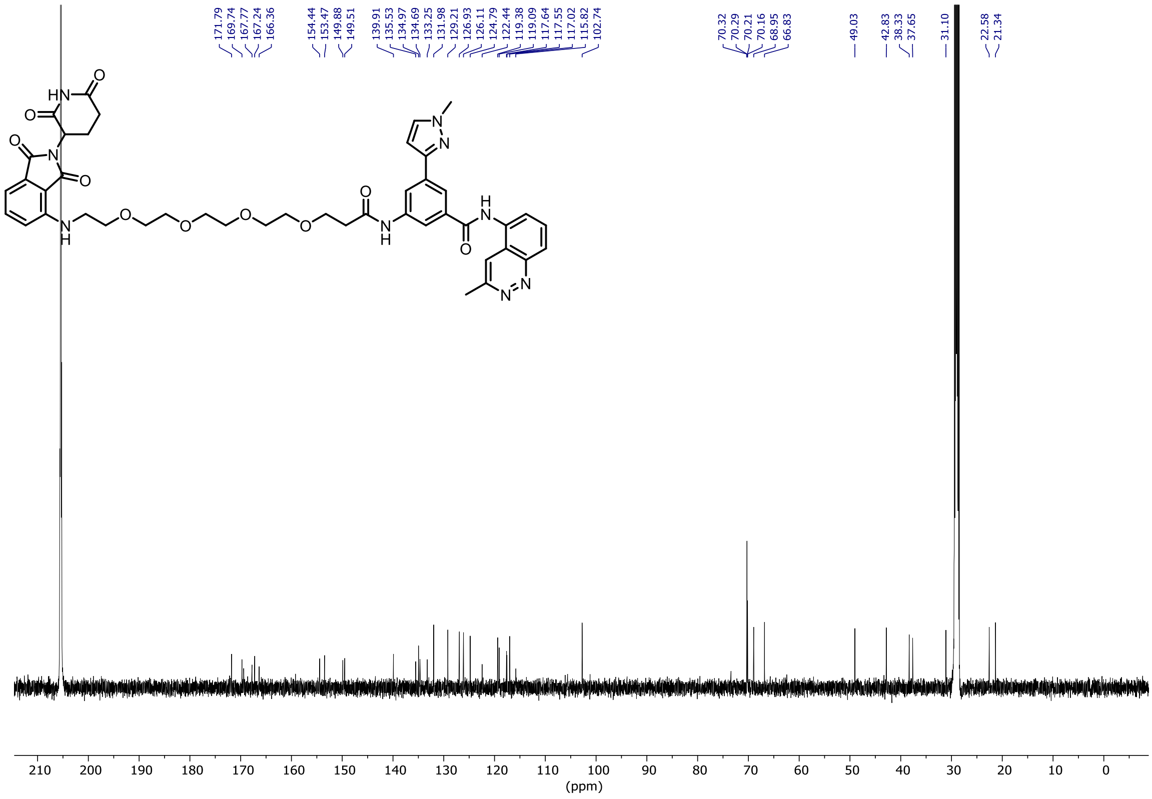

Compound S24. ^{1}H NMR (500 MHz, DMSO-d_6) and ^{13}C NMR (126 MHz, DMSO-d_6).

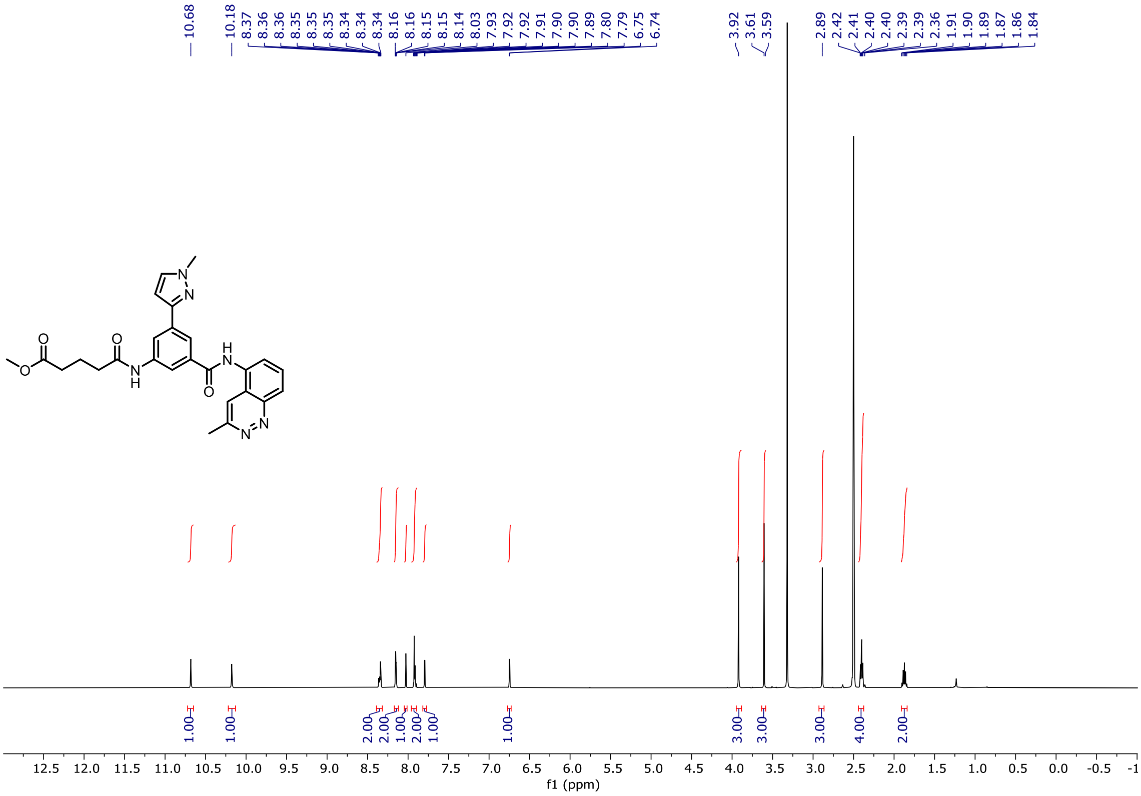

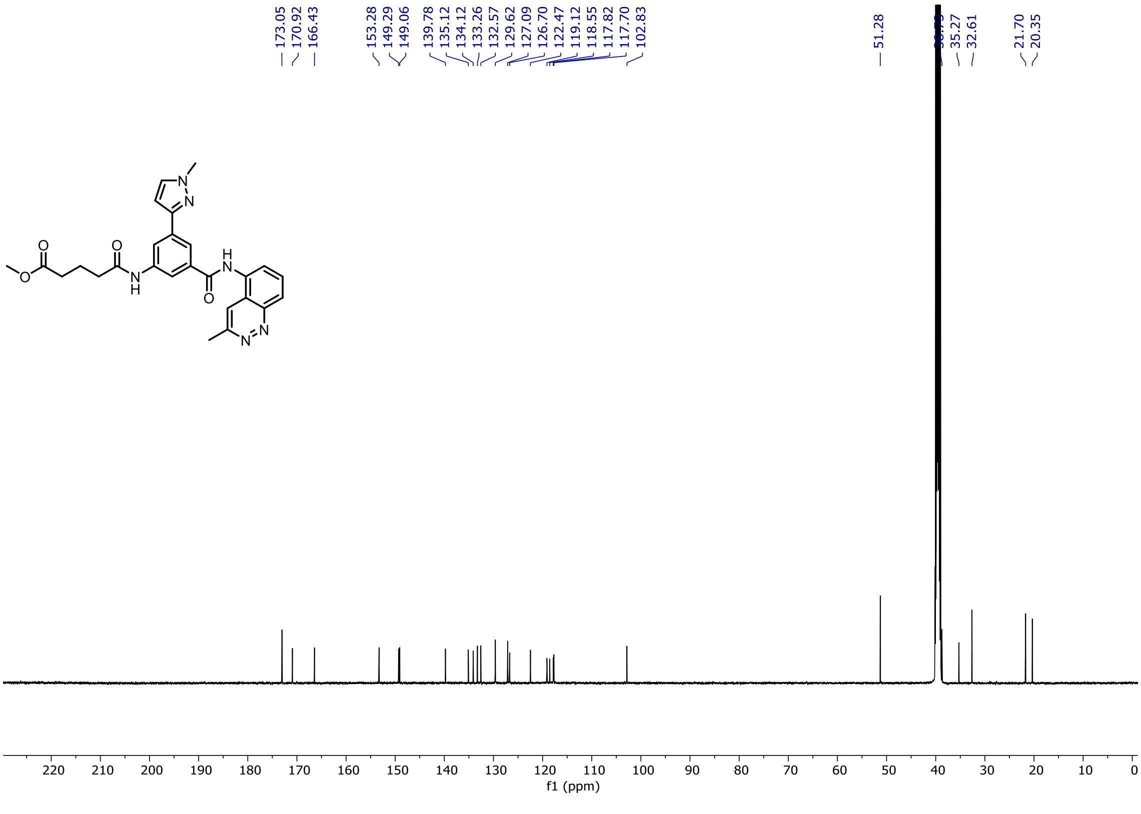

Compound S2. ^{1}H NMR (500 MHz, CD_2Cl_2) and ^{13}C NMR (126 MHz, CD_2Cl_2).

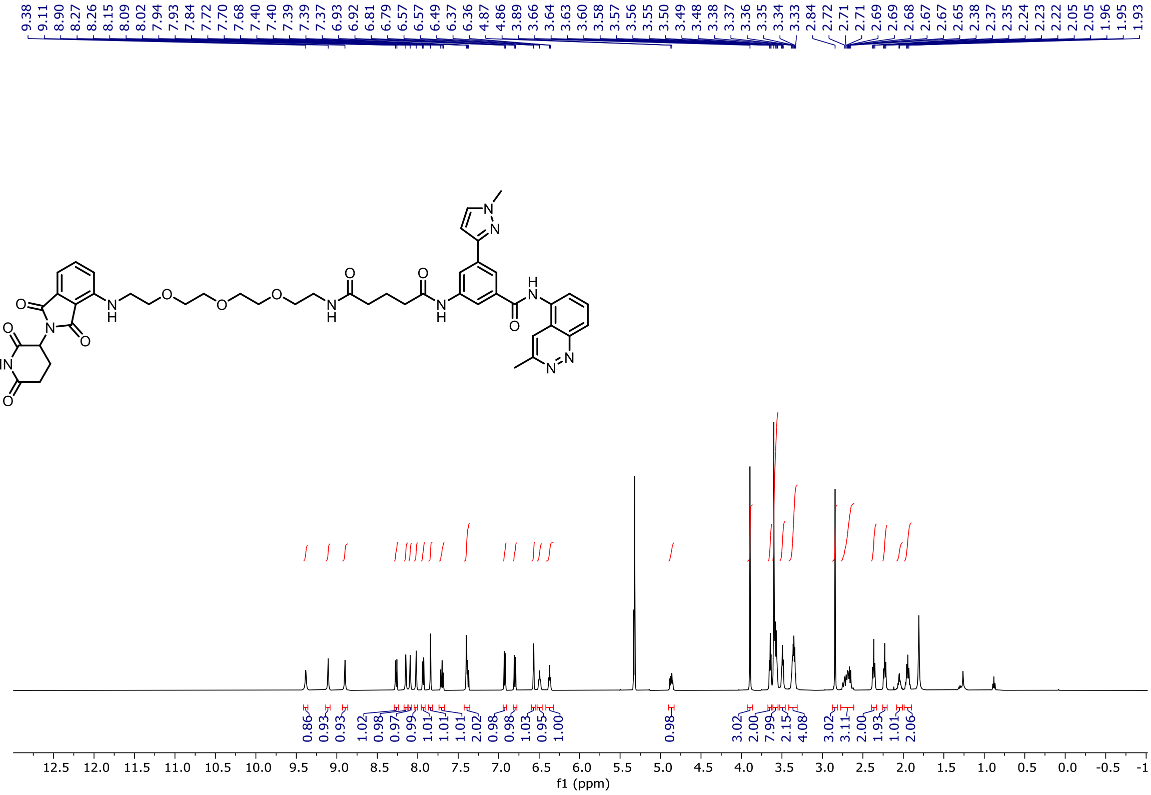

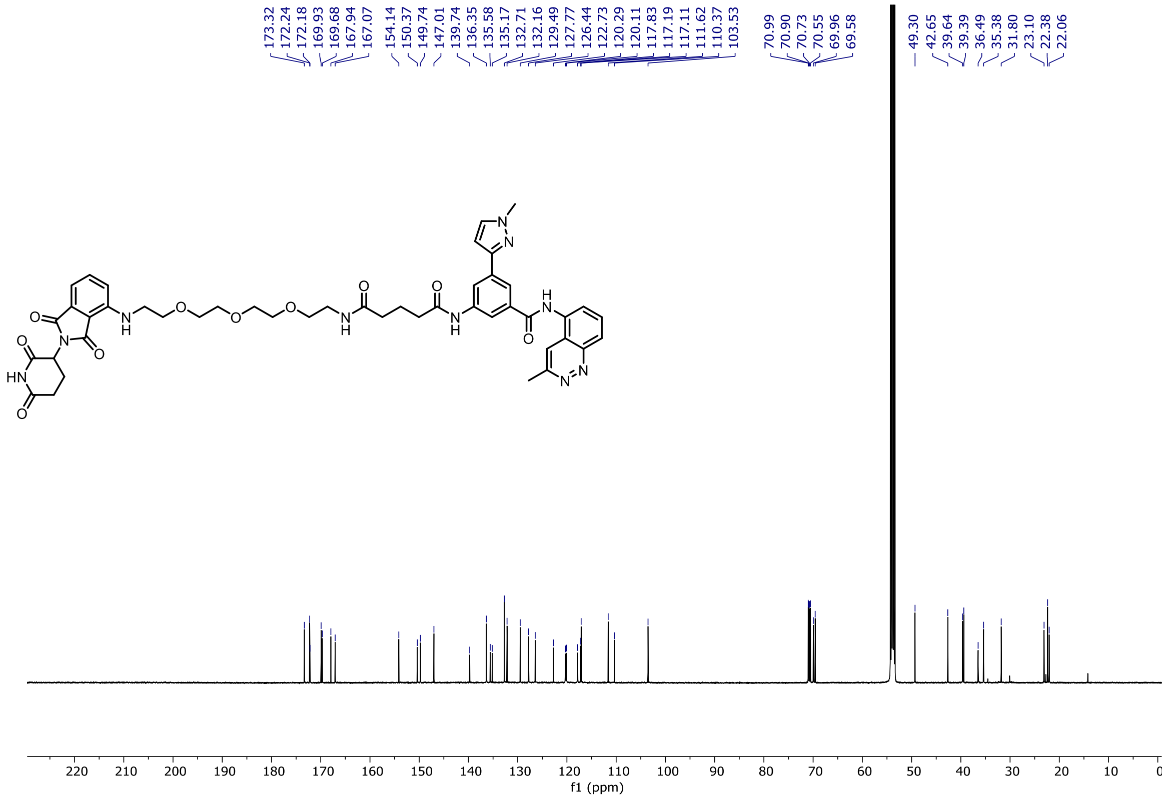

Compound S30. ^{1}HNMR (500 MHz, $CDCl_3$) and ^{13}C NMR (126 MHz, $CDCl_3$).

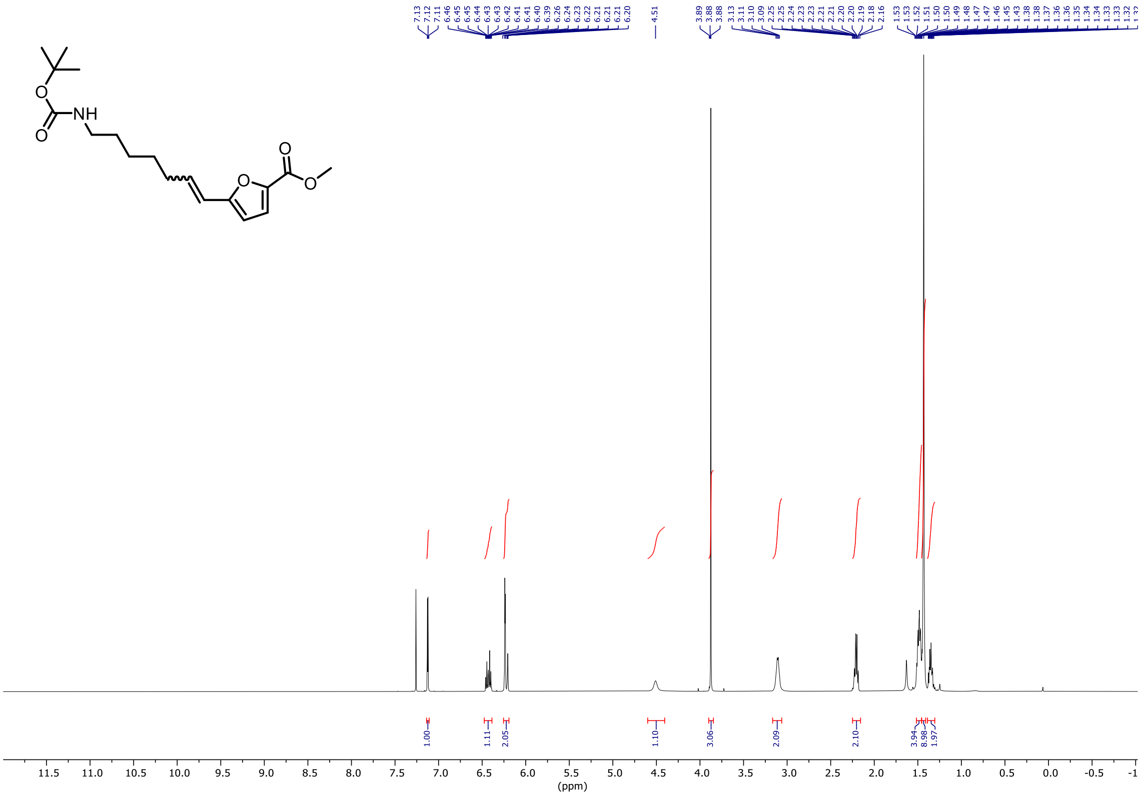

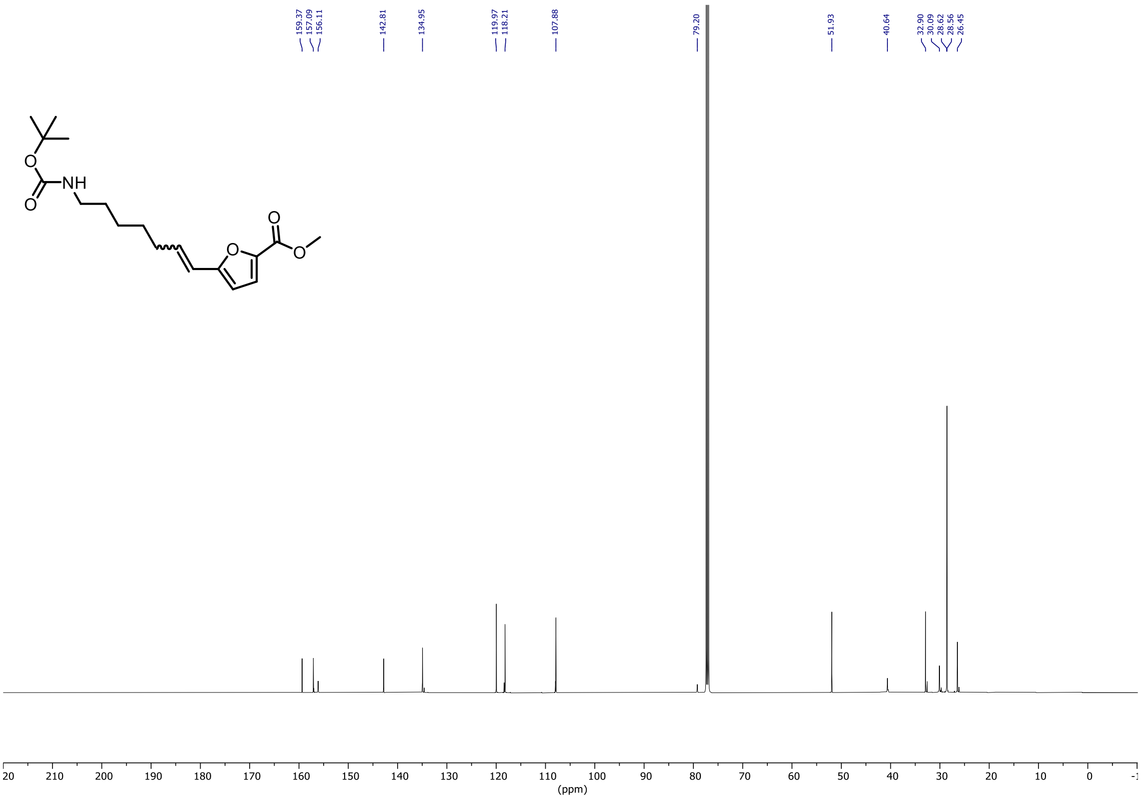

Compound S3 . ^{1}HNMR (500 MHz, Methanol-*d*$_4$) and ^{13}C NMR (126 MHz, CD_2Cl_2).

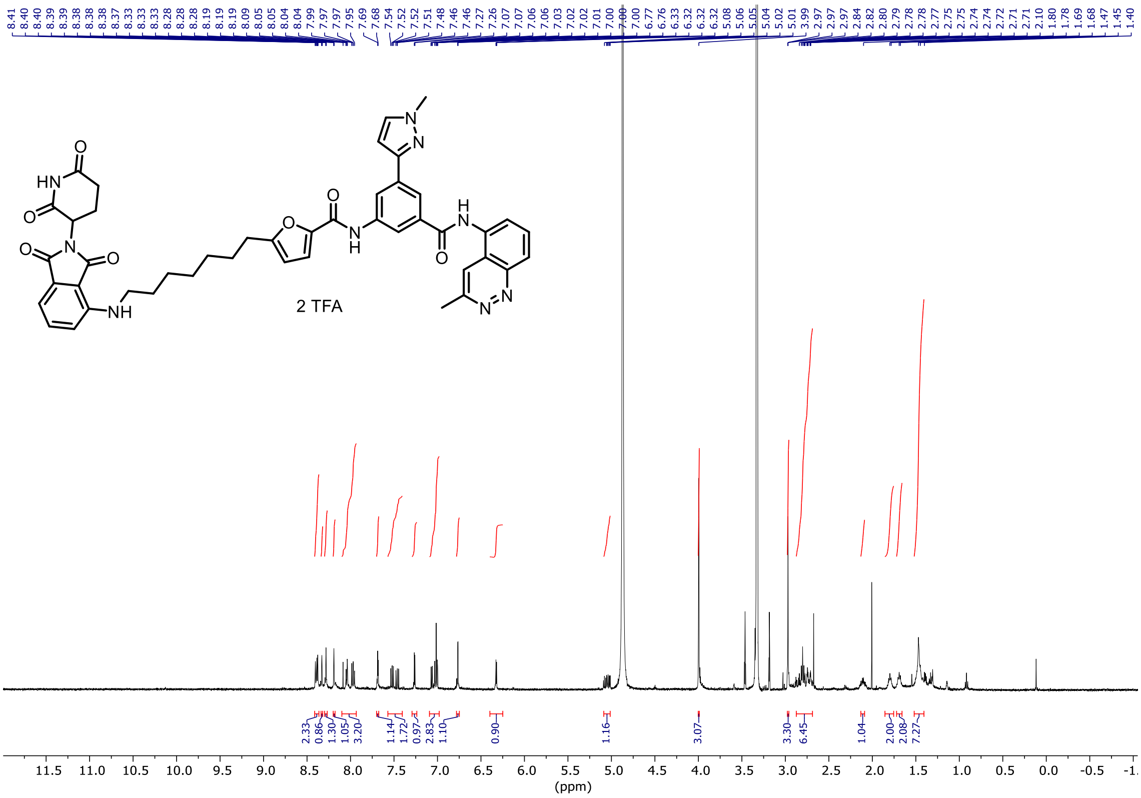

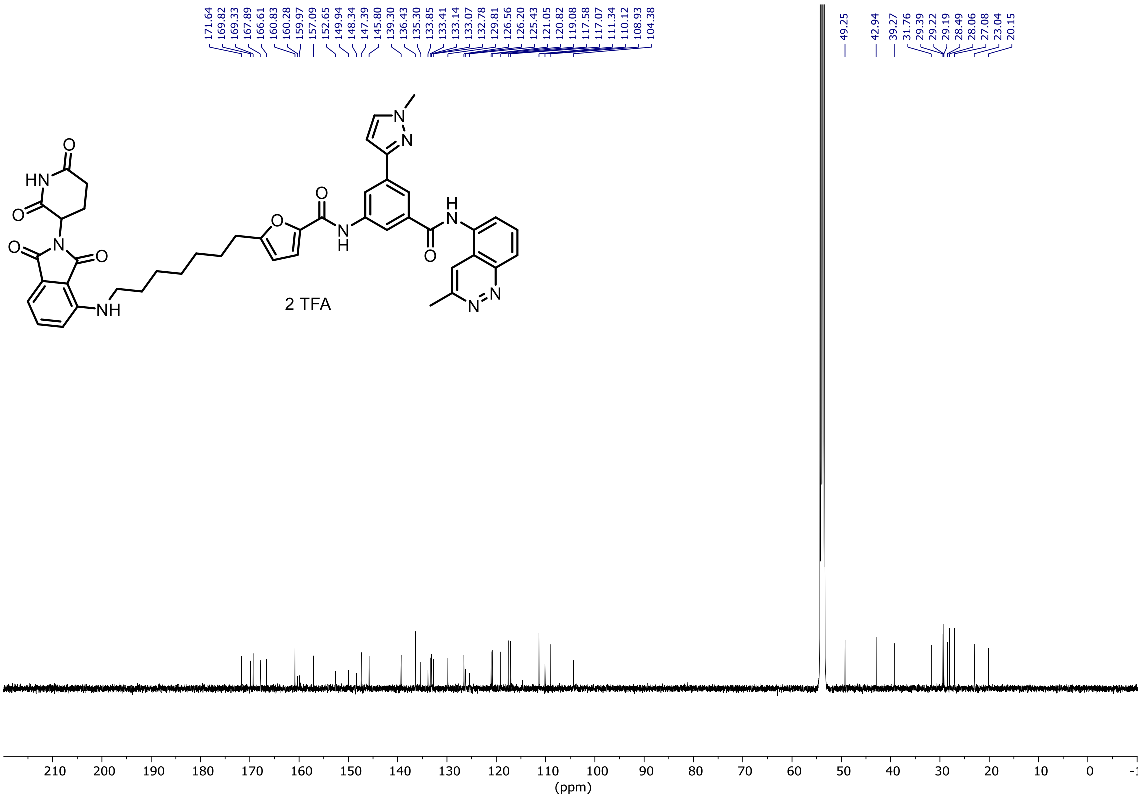

Compound S4. . ^{1}HNMR (500 MHz, DMSO-d_6) and ^{13}C NMR (126 MHz, DMSO-d_6).

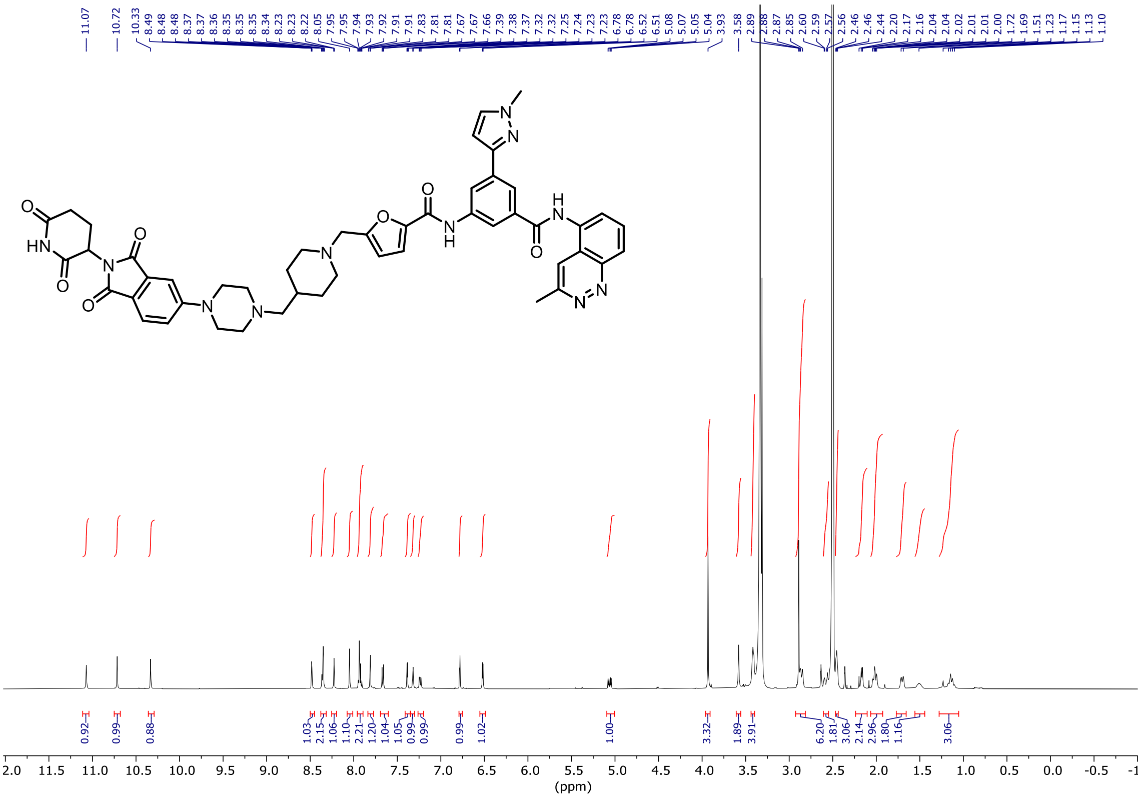

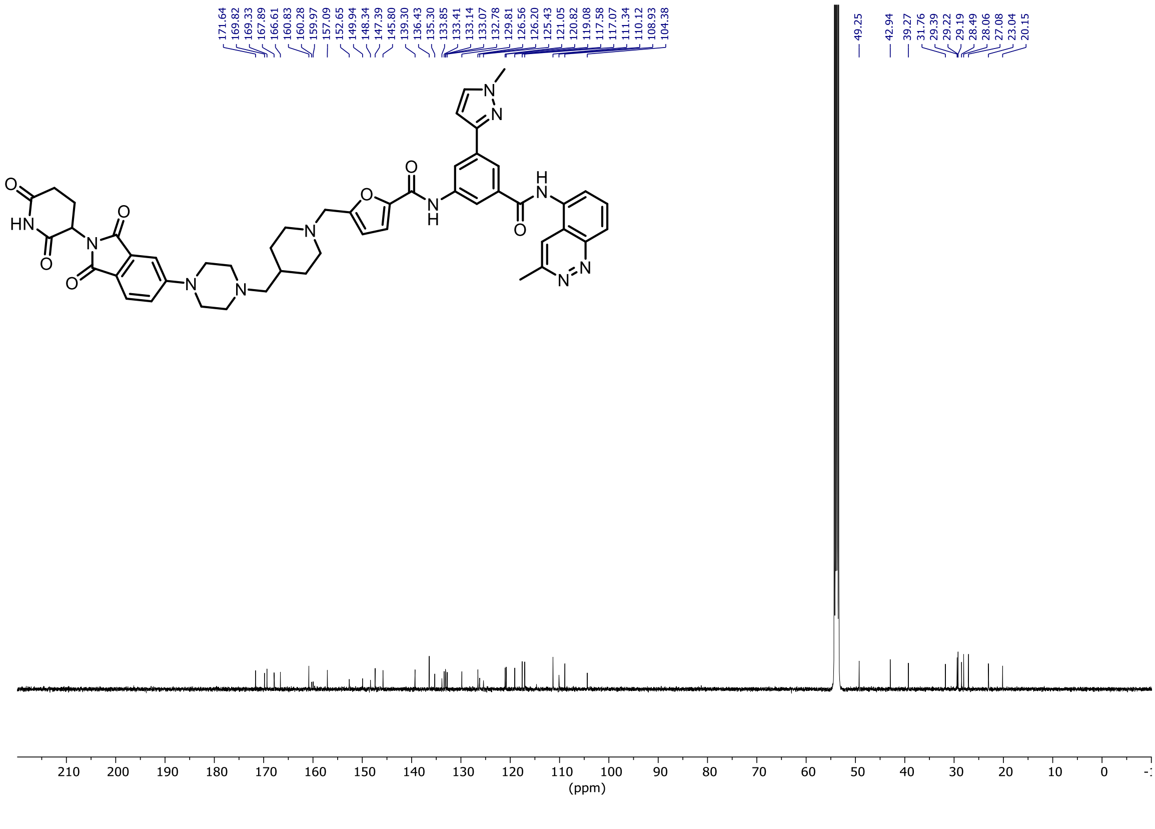

Compound S10. ^{1}HNMR (400 MHz, $CDCl_3$) and ^{13}C NMR (100 MHz, $CDCl_3$).

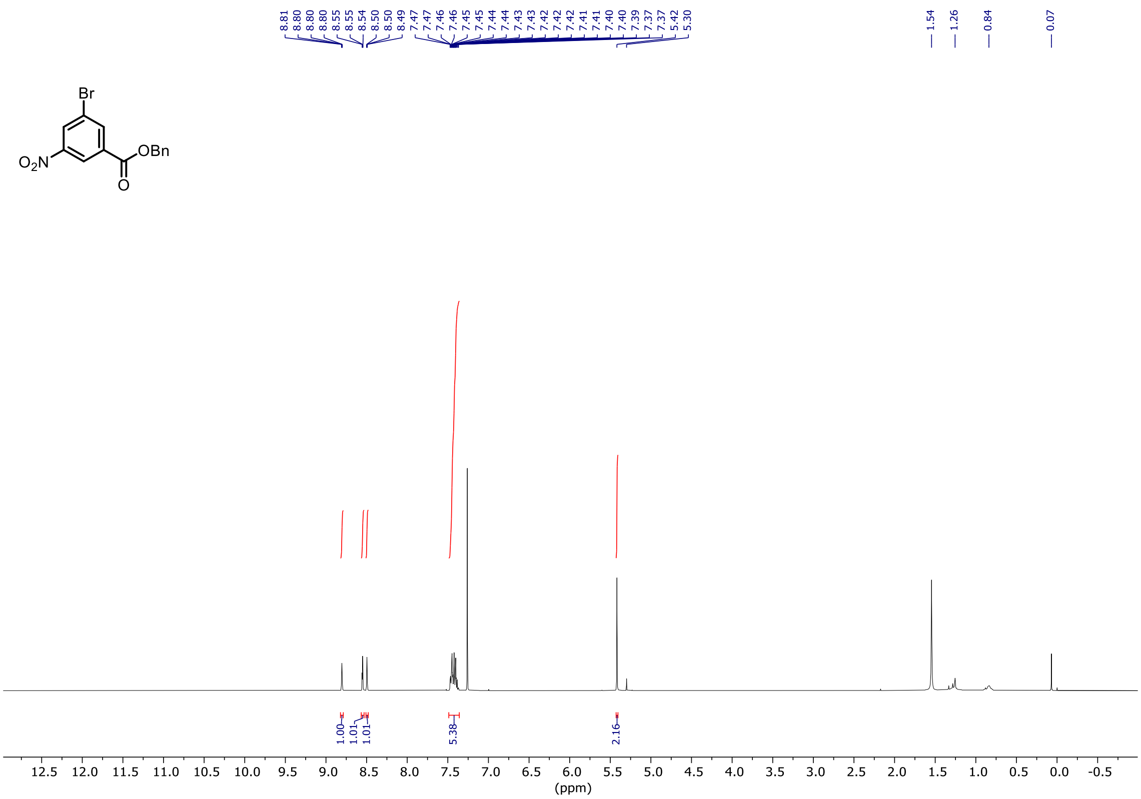

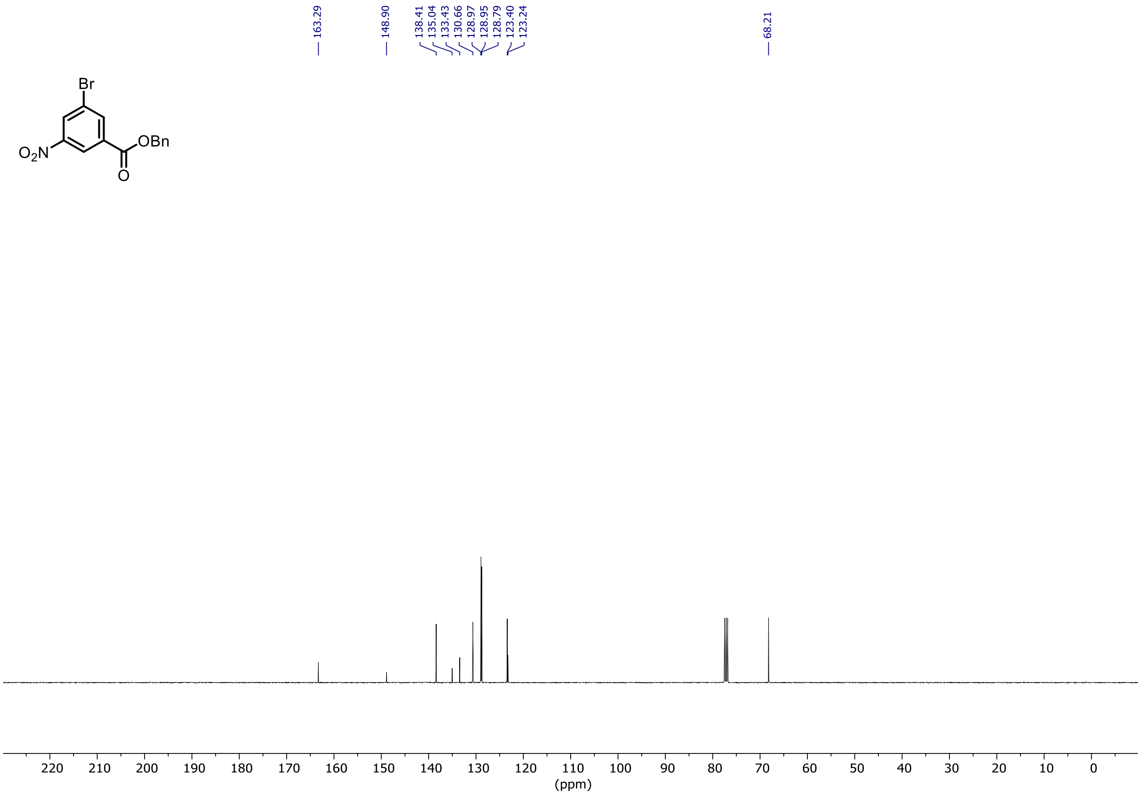

Compound S11. ^{1}HNMR (400 MHz, DMSO-d_6) and ^{13}C NMR (100 MHz, $CDCl_3$).

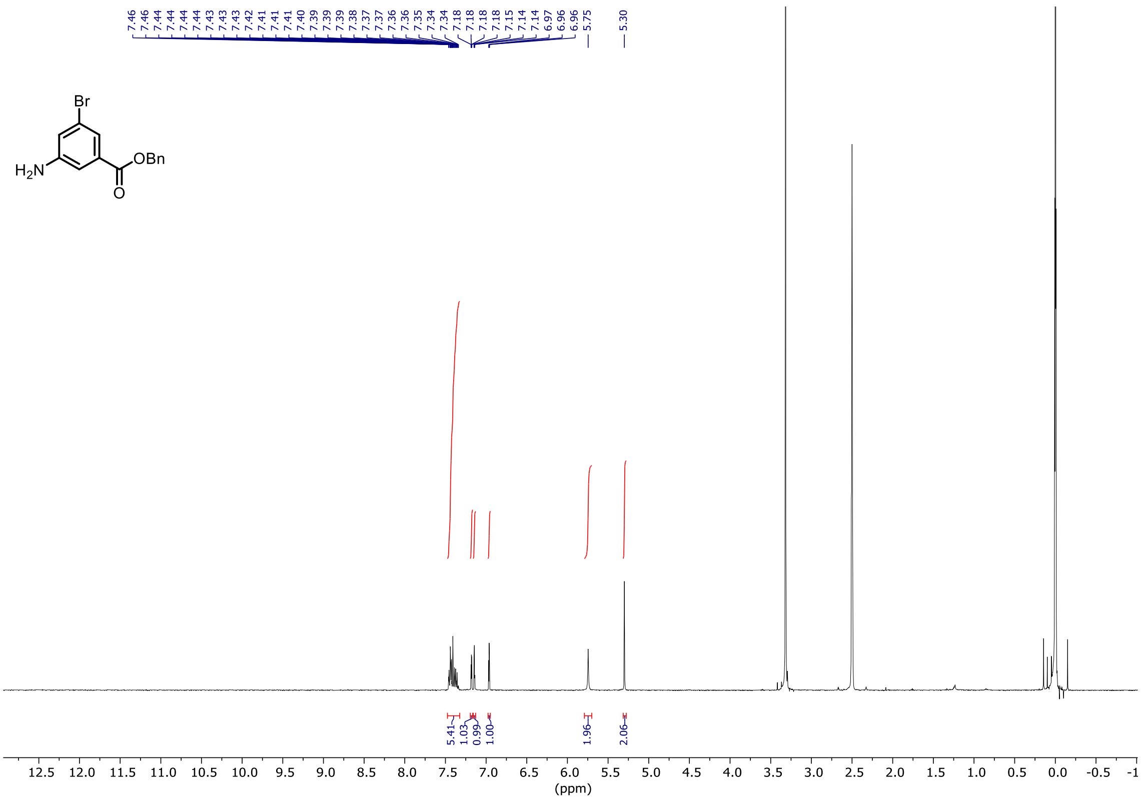

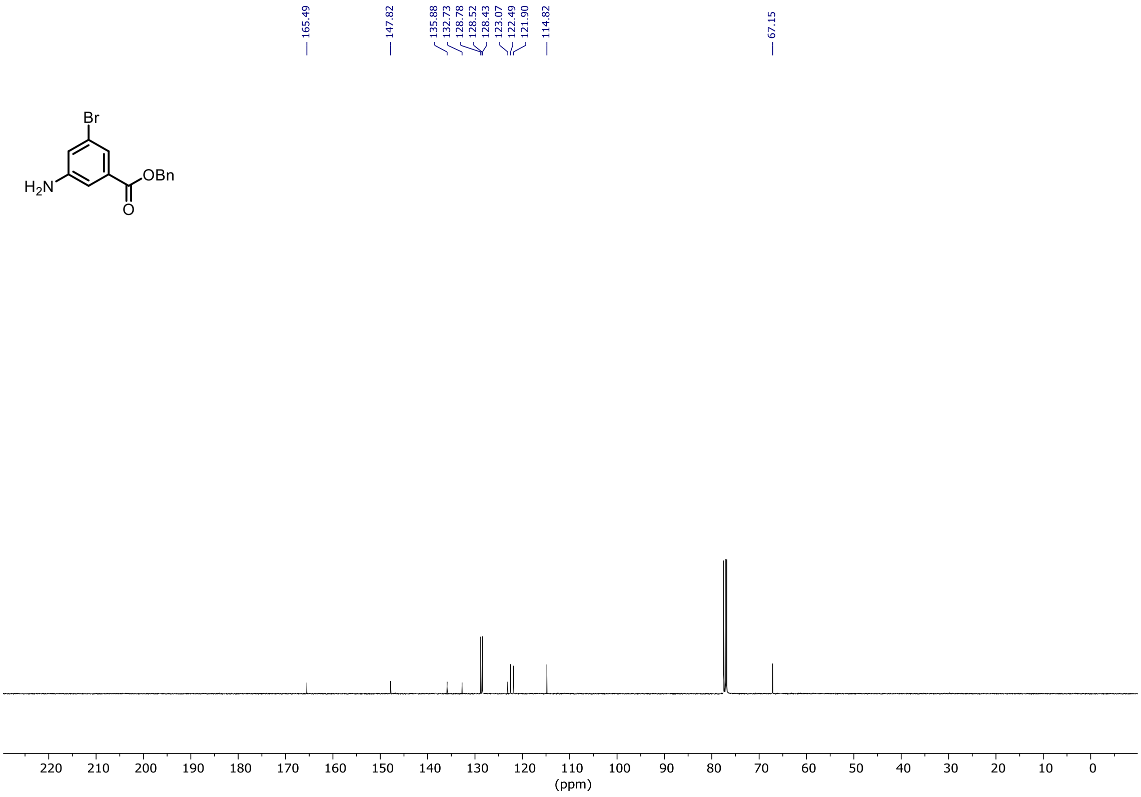

Compound S12. ^{1}HNMR (400 MHz, DMSO-d_6) and ^{13}C NMR (100 MHz, $CDCl_3$).

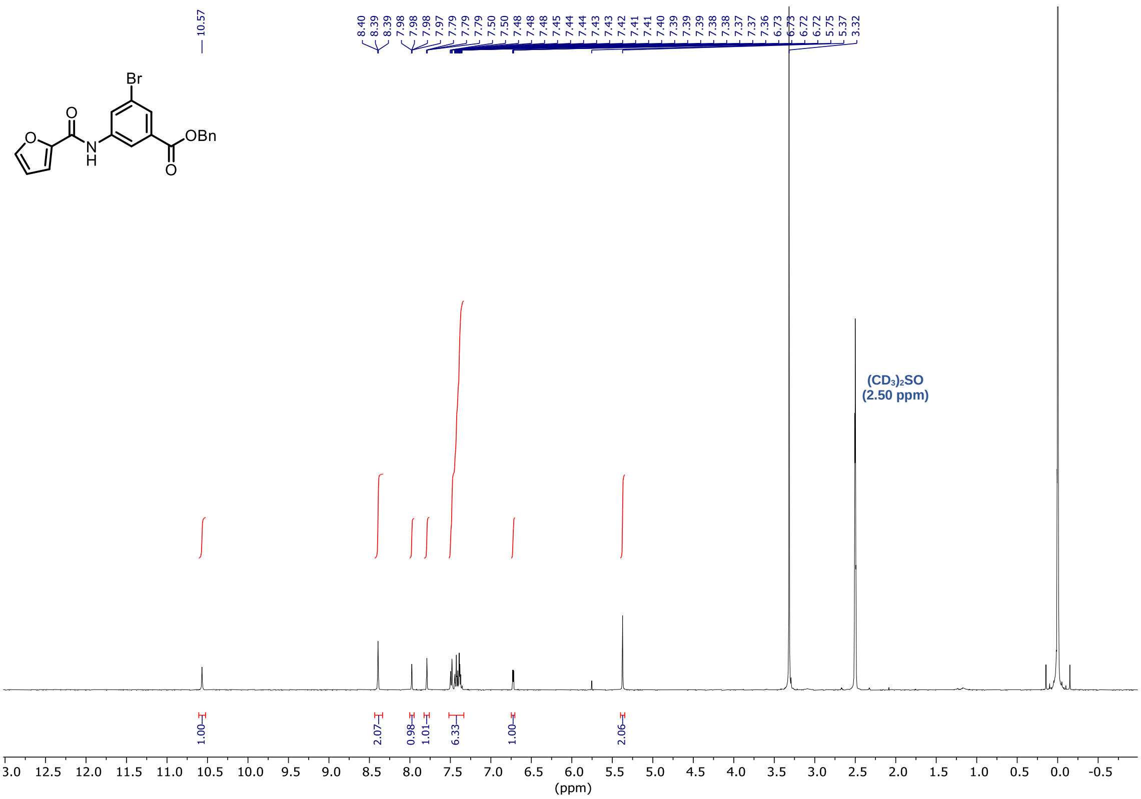

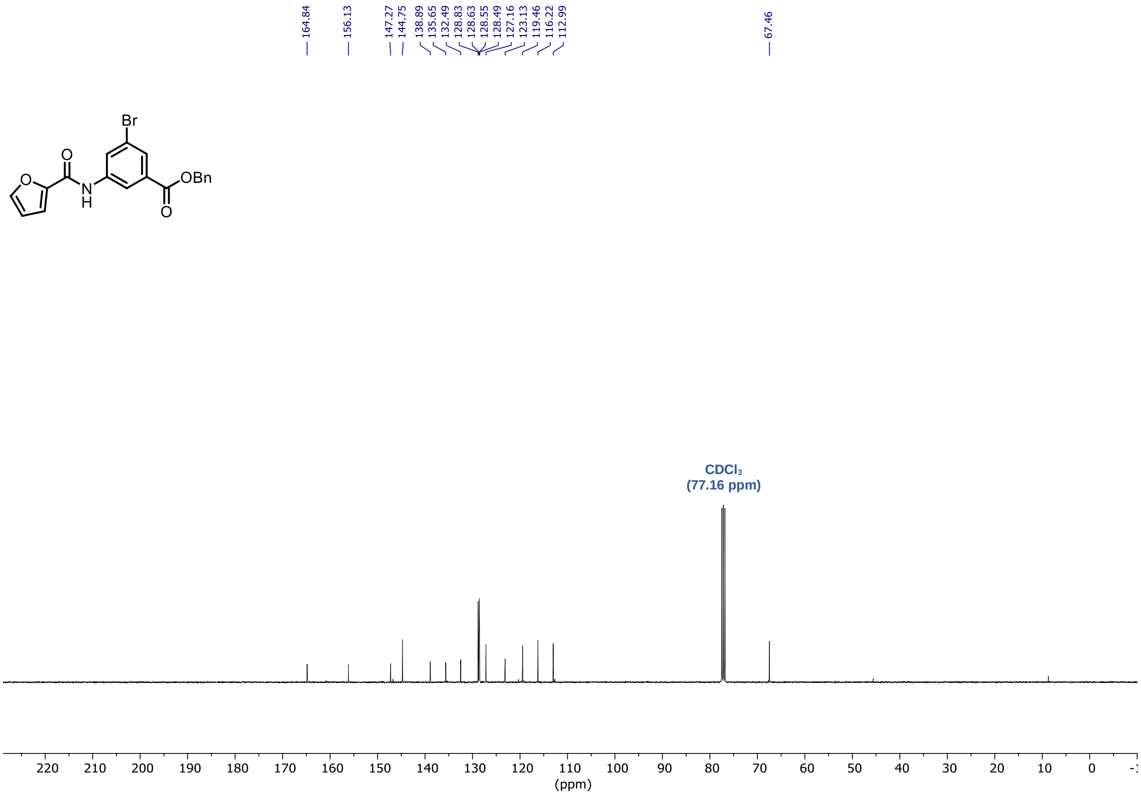

Compound S13. ^{1}HNMR (400 MHz, $CDCl_3$) and ^{13}C NMR (100 MHz, $CDCl_3$).

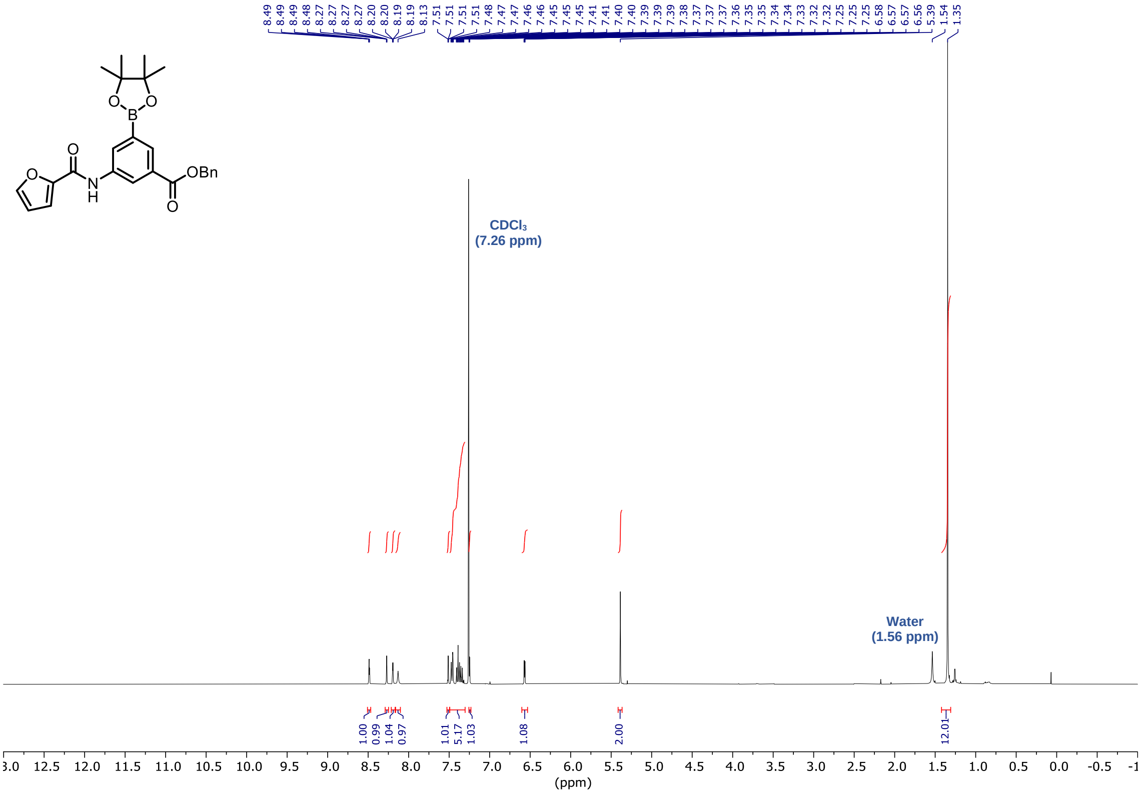

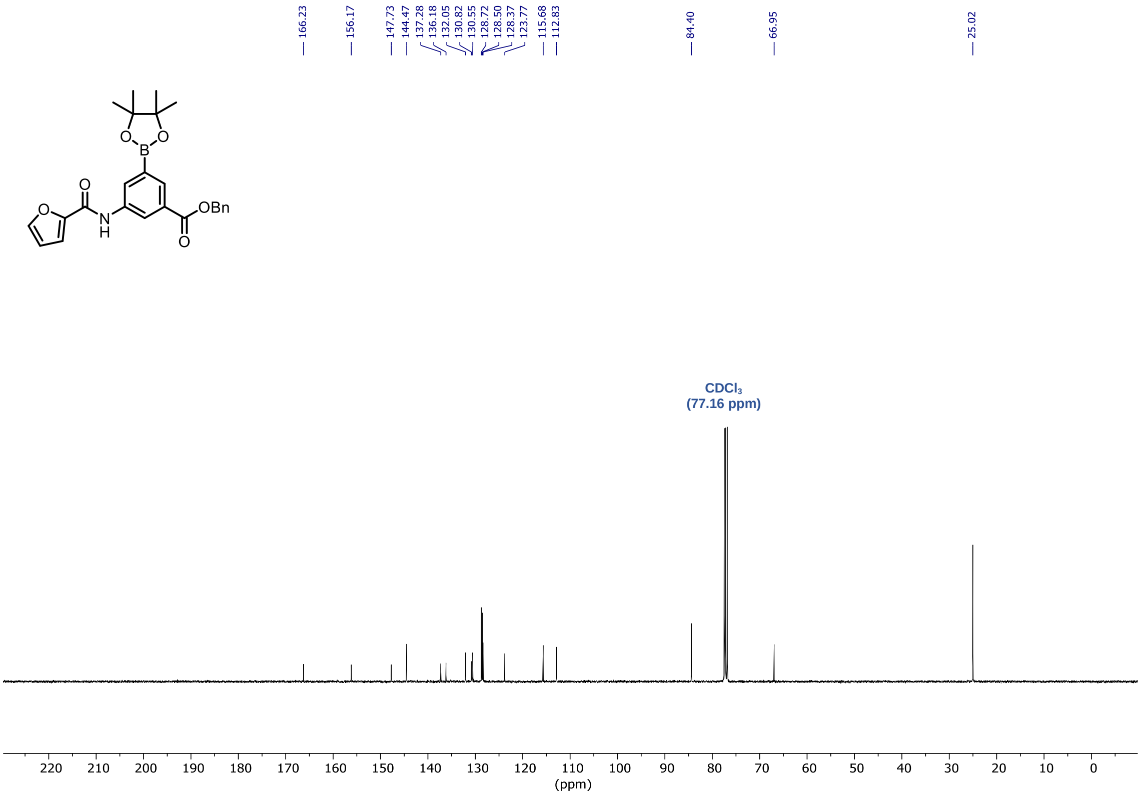

Compound S14. ^{1}HNMR (500 MHz, $CDCl_3$) and ^{13}C NMR (126 MHz, $CDCl_3$).

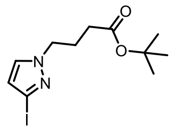

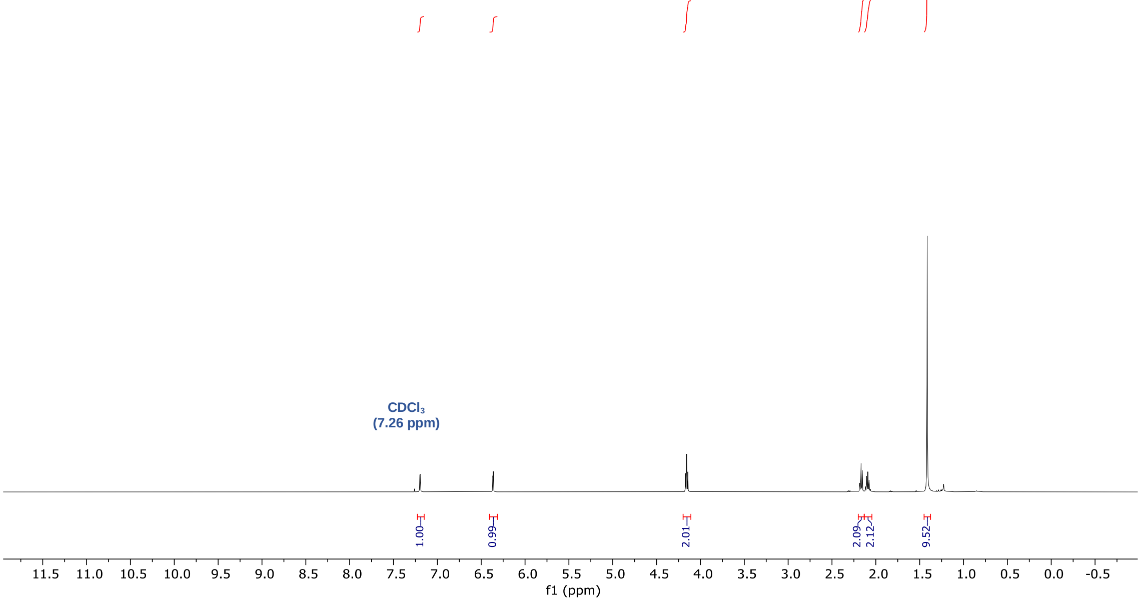

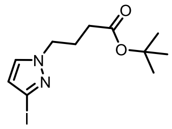

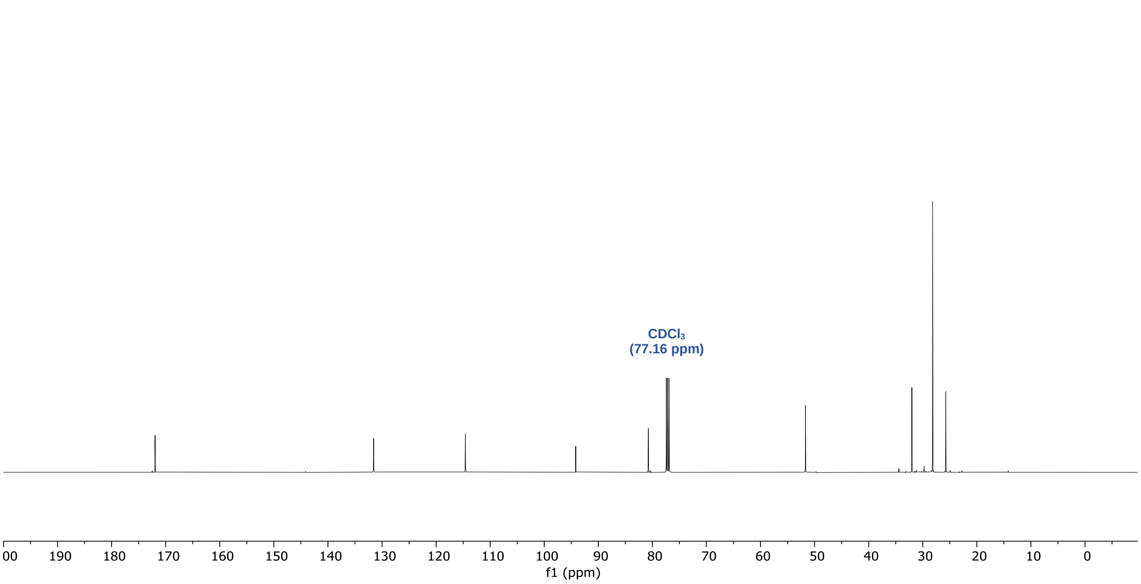

Compound S15. ^{1}HNMR (400 MHz, $CDCl_3$) and ^{13}C NMR (100 MHz, $CDCl_3$).

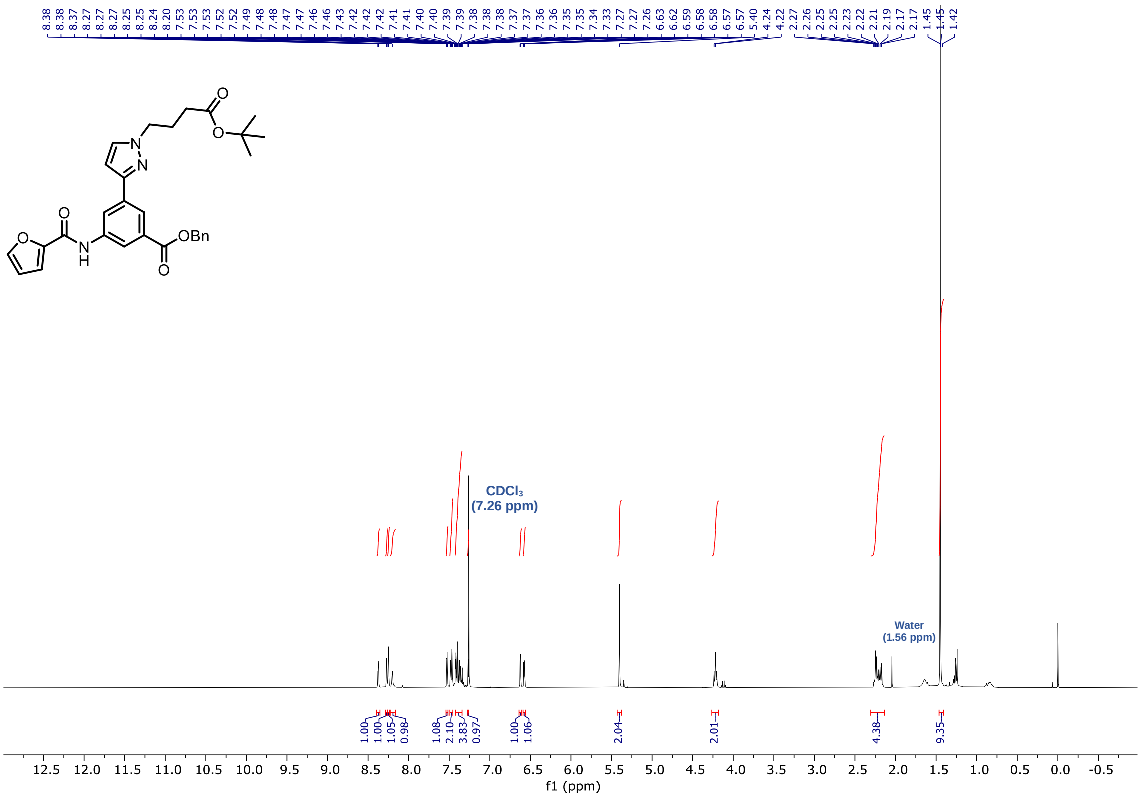

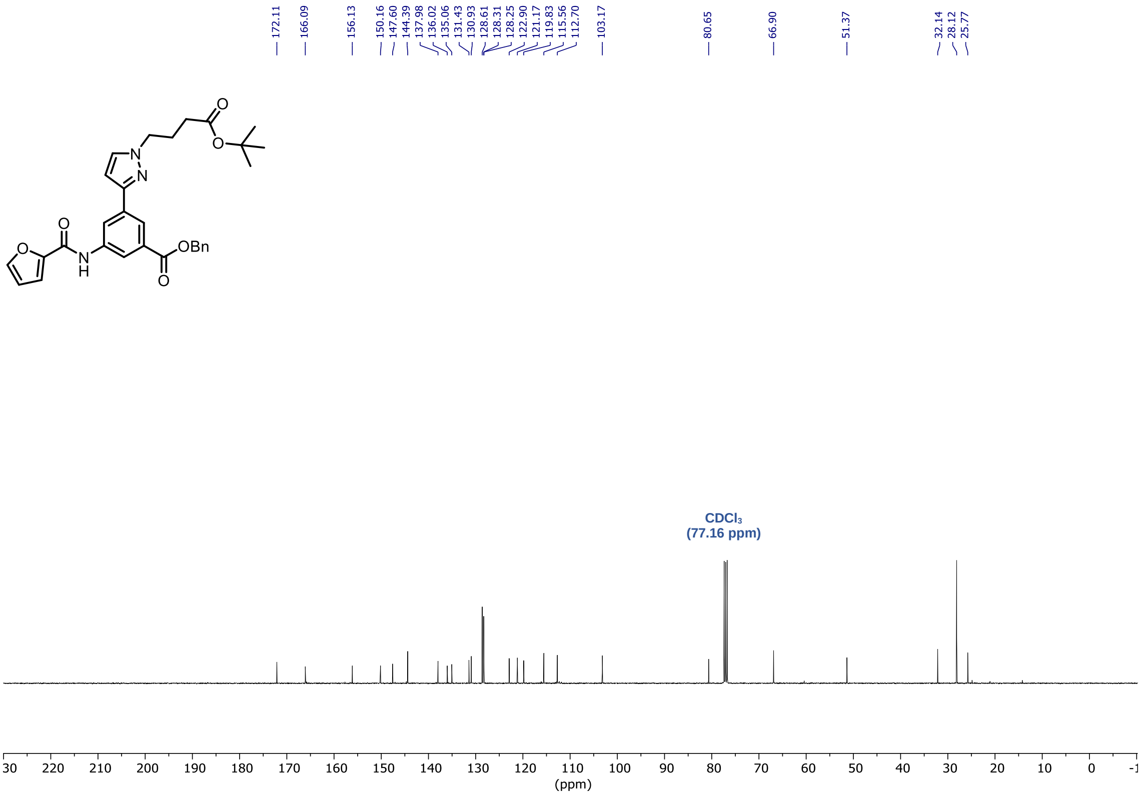

Compound S16. ^{1}HNMR (400 MHz, DMSO-d_6) and ^{13}C NMR (100 MHz, Methanol-d_4).

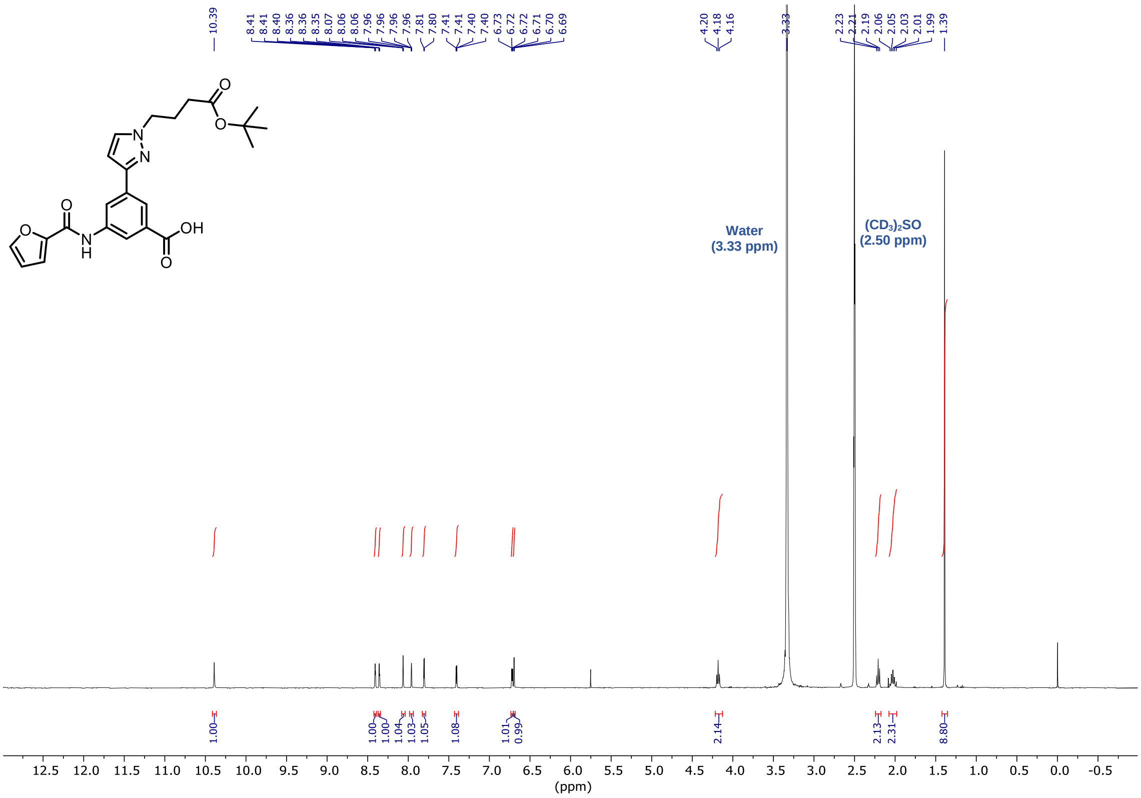

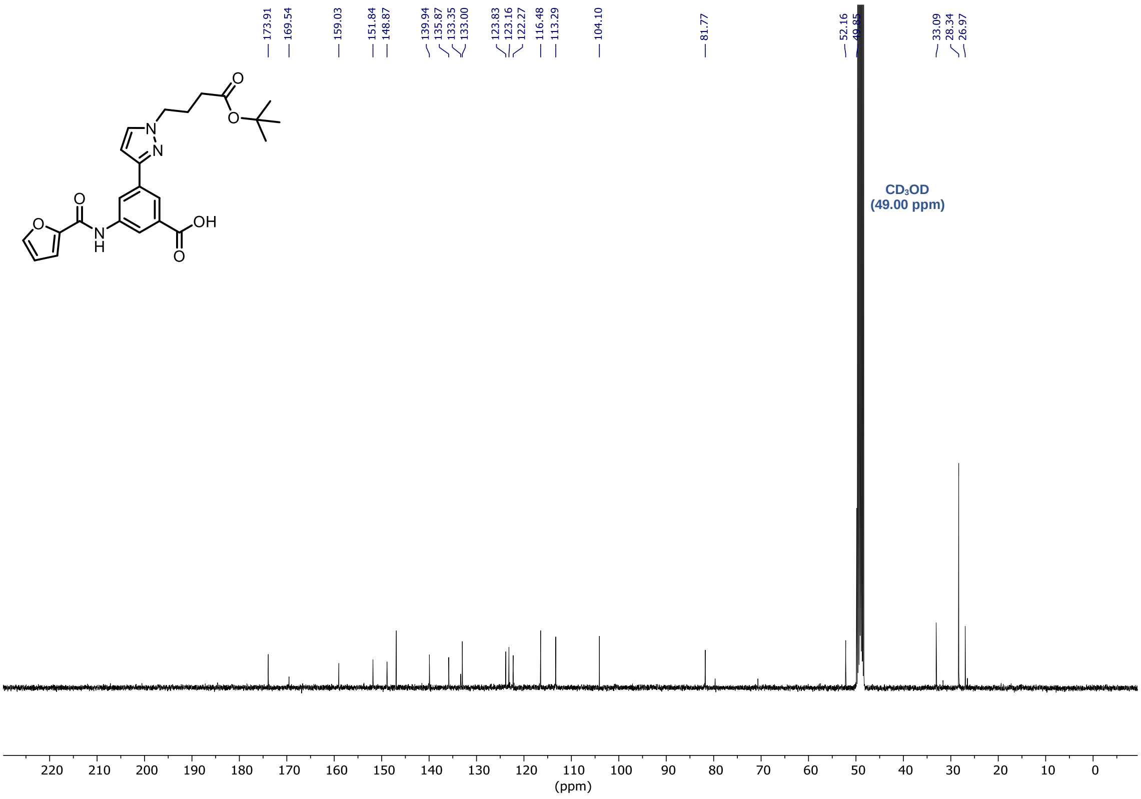

Compound S17. ^{1}HNMR (400 MHz, Methanol-d_4) and ^{13}C NMR (100 MHz, Methanol-d_4).

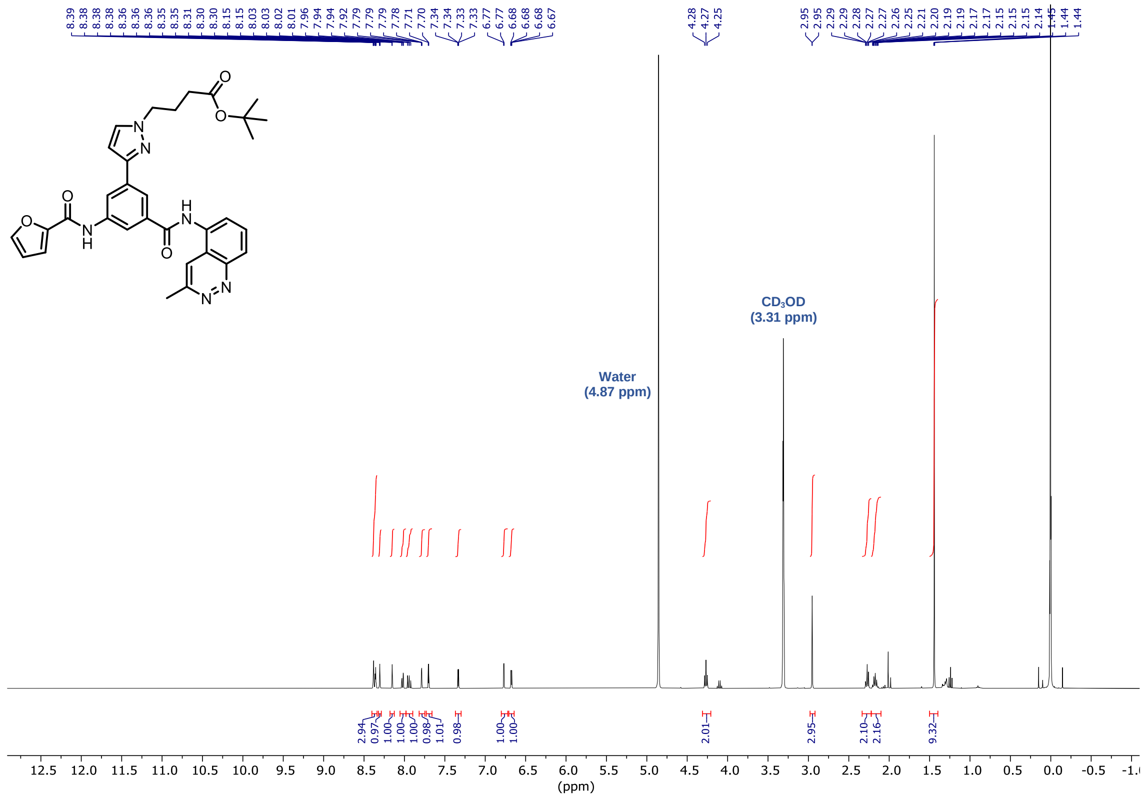

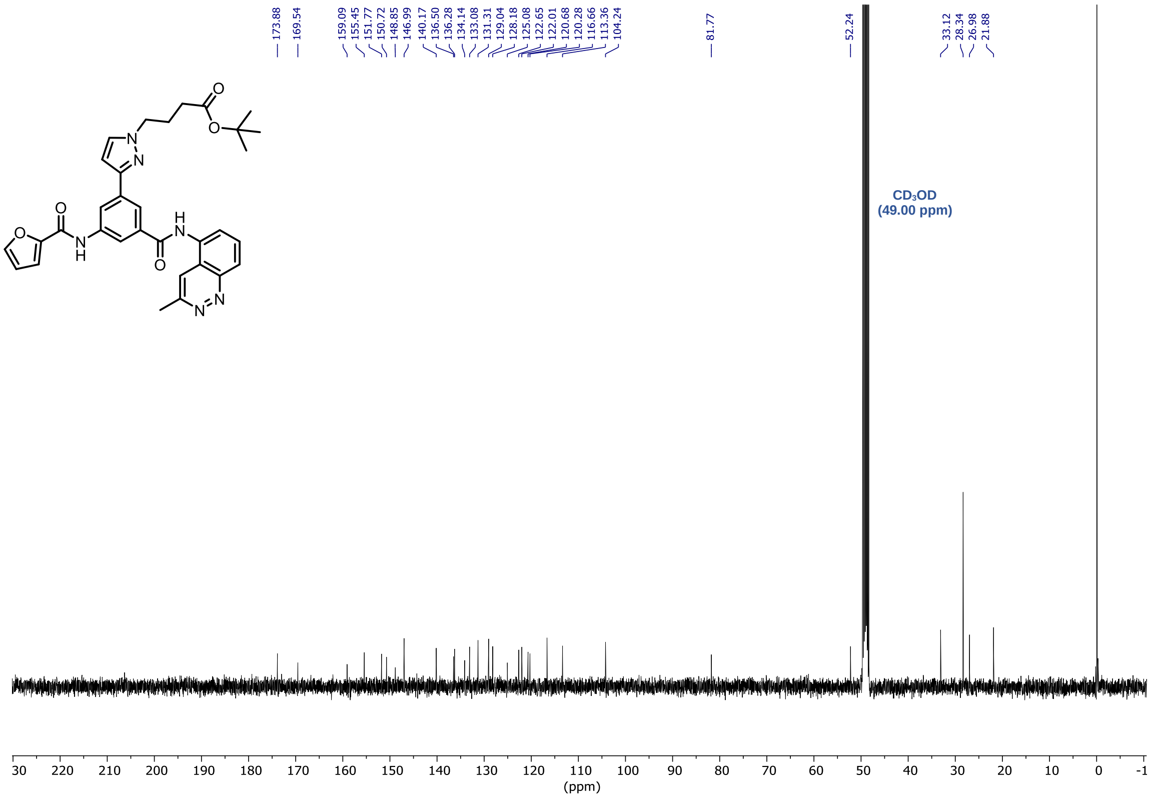

Compound S5. ^{1}HNMR (400 MHz, Acetone-d_6) and ^{13}C NMR (126 MHz, Acetone-d_6).

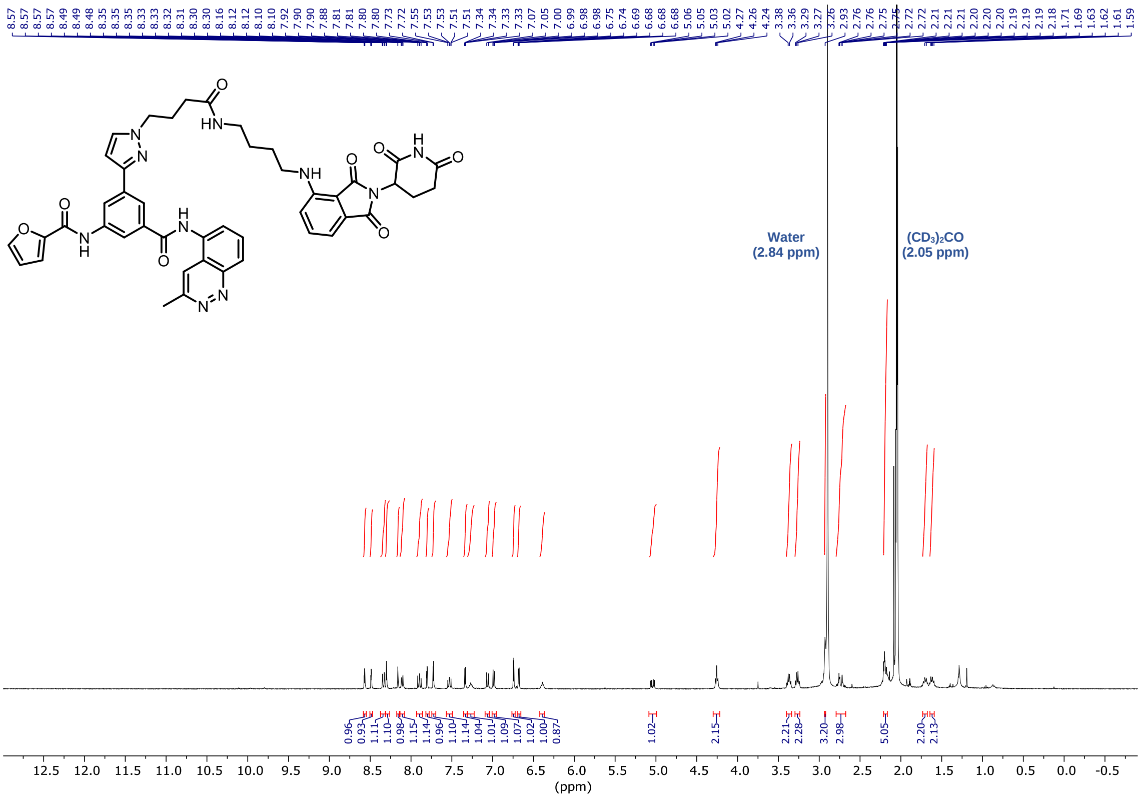

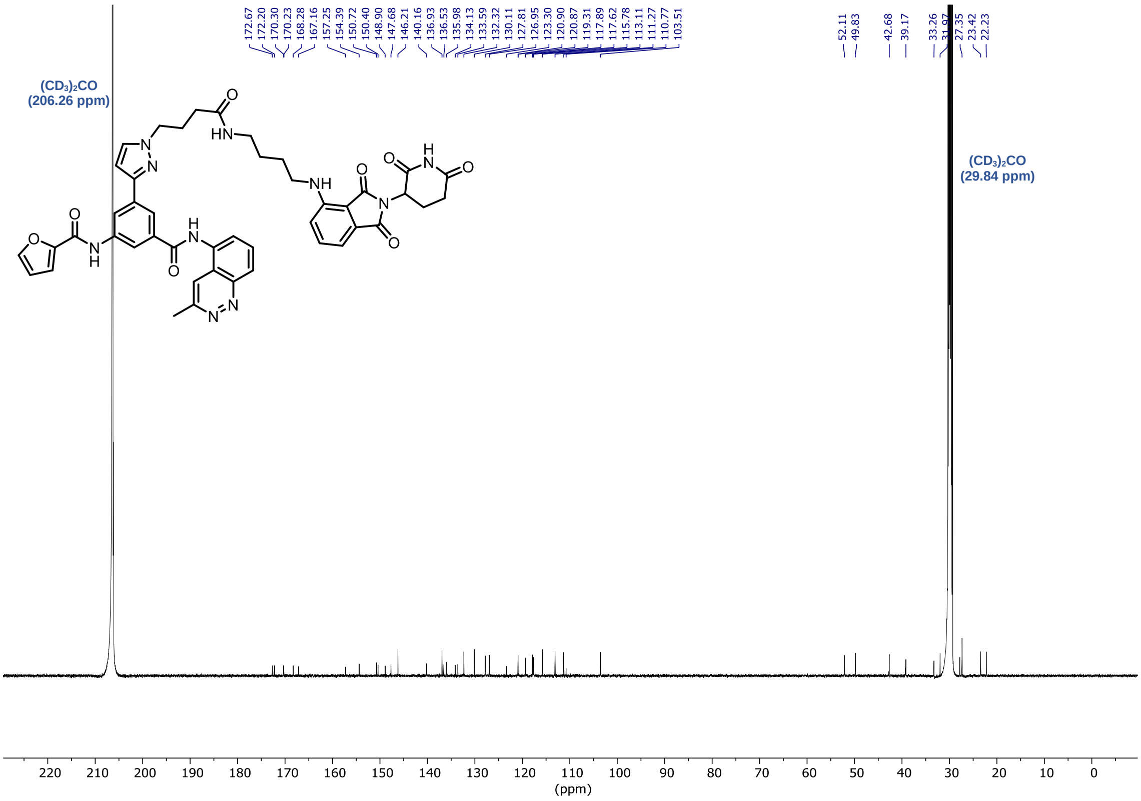

Compound S6. ^{1}HNMR (400 MHz, Acetone-d_6) and ^{13}C NMR (126 MHz, Acetone-d_6).

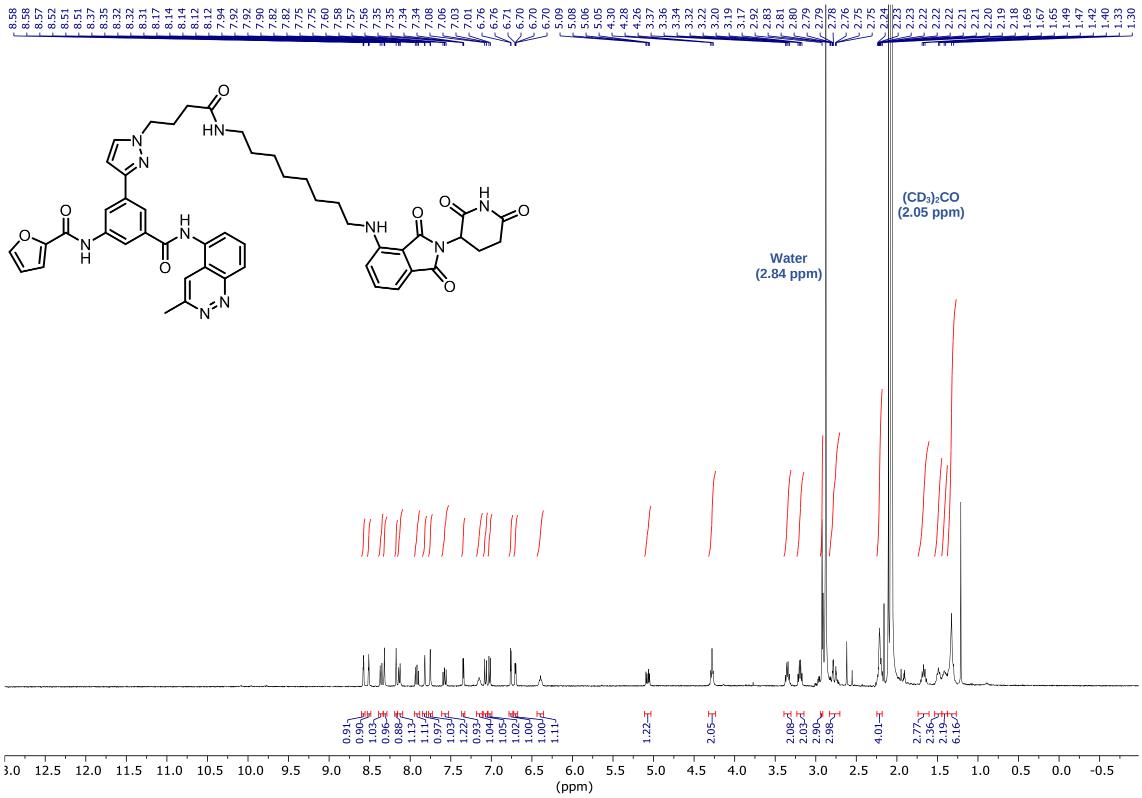

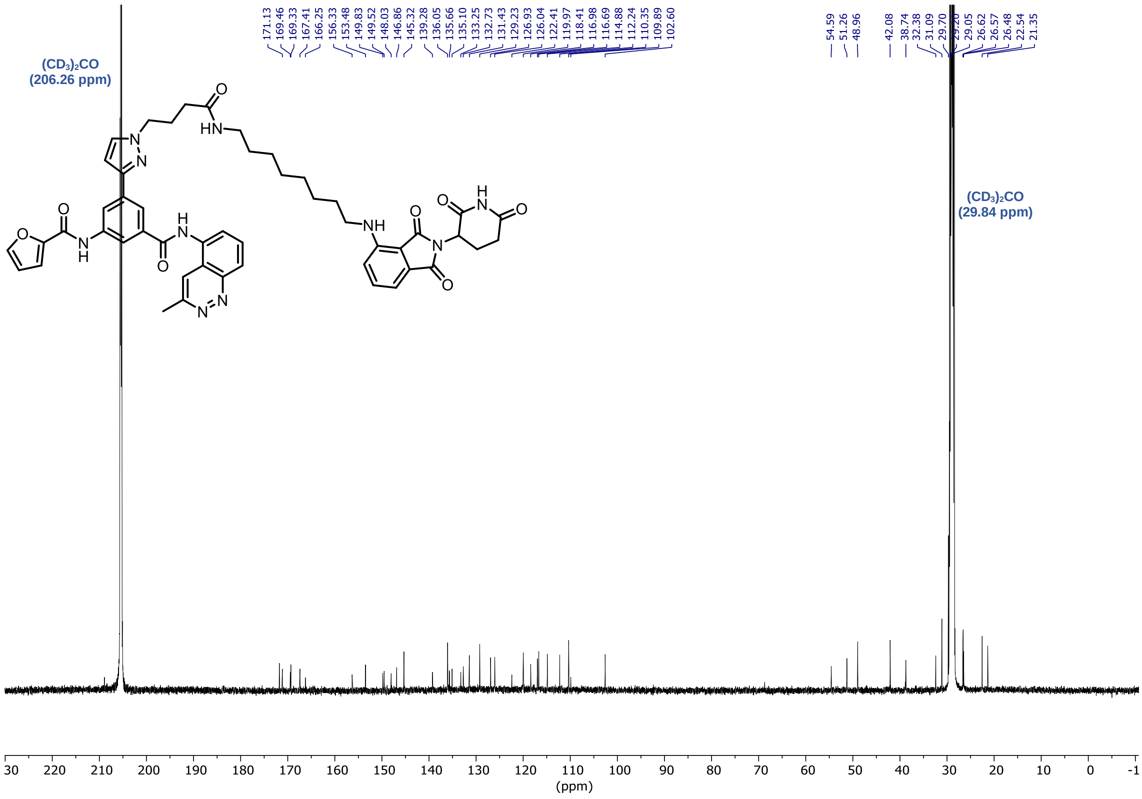

Compound S7. ^{1}HNMR (400 MHz, Acetone-d_6) and ^{13}C NMR (126 MHz, Acetone-d_6).

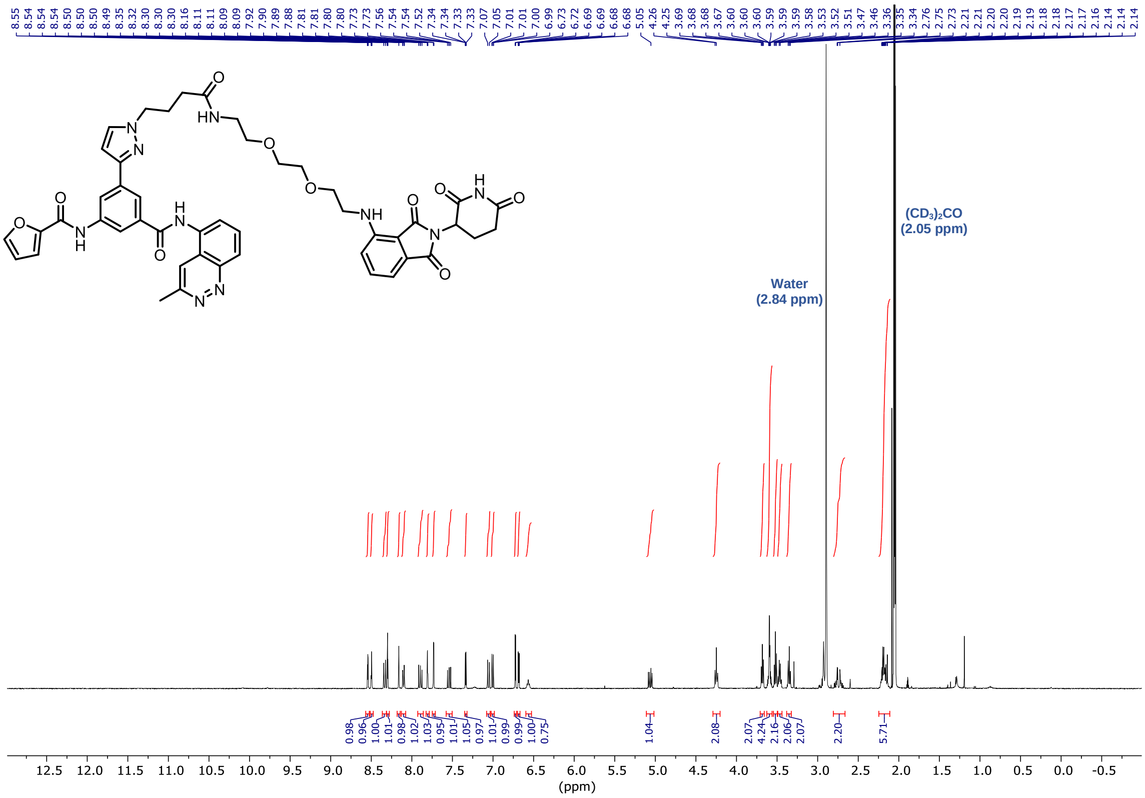

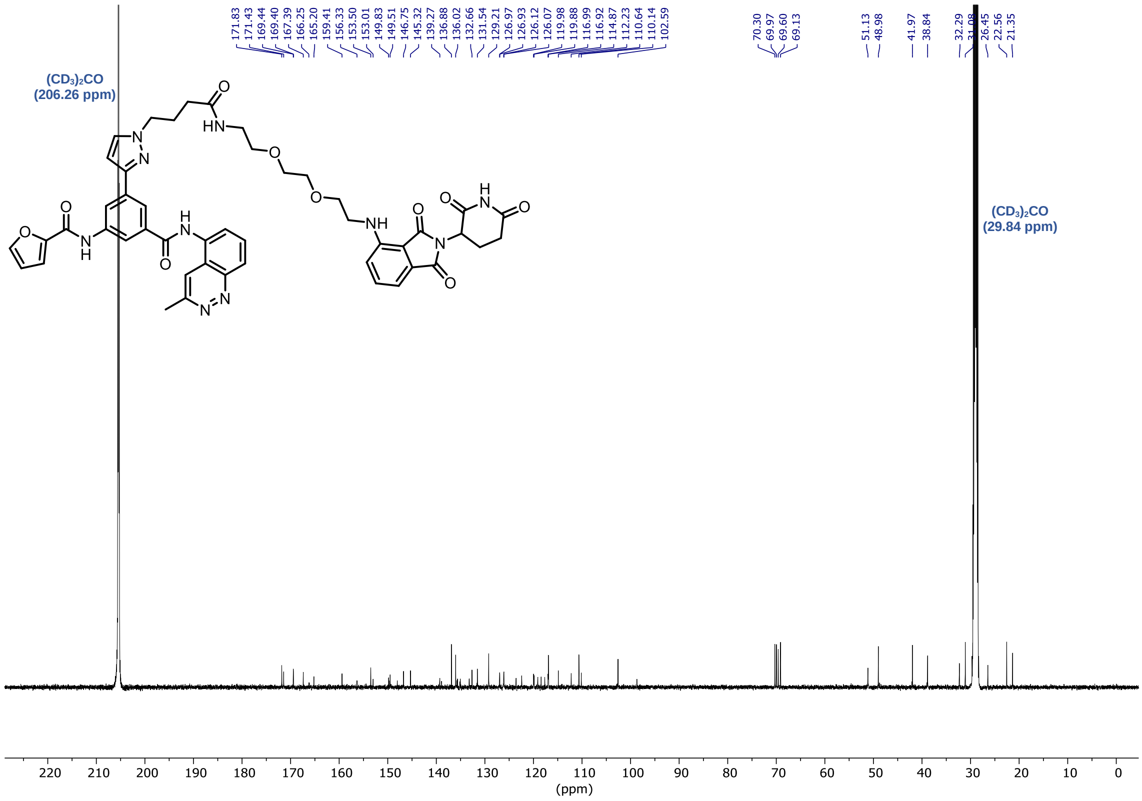

Compound S8. ^{1}HNMR (400 MHz, Acetone-d_6) and ^{13}C NMR (126 MHz, Acetone-d_6).

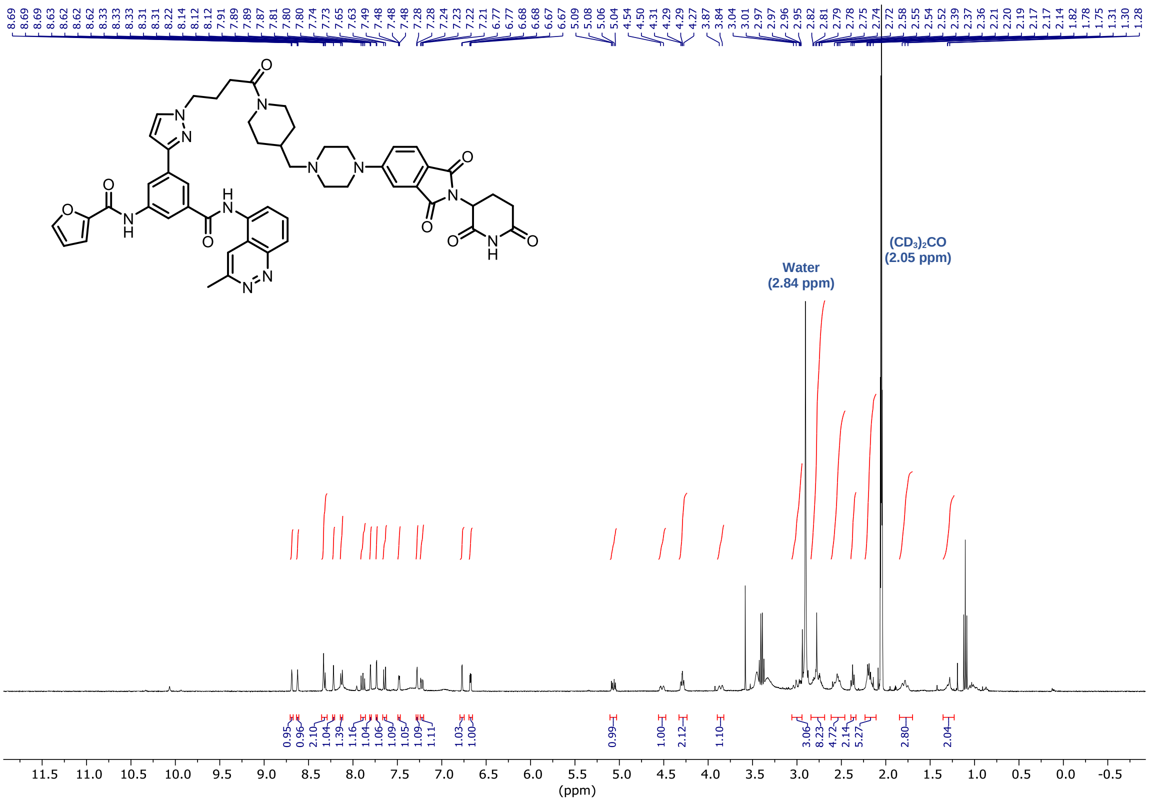

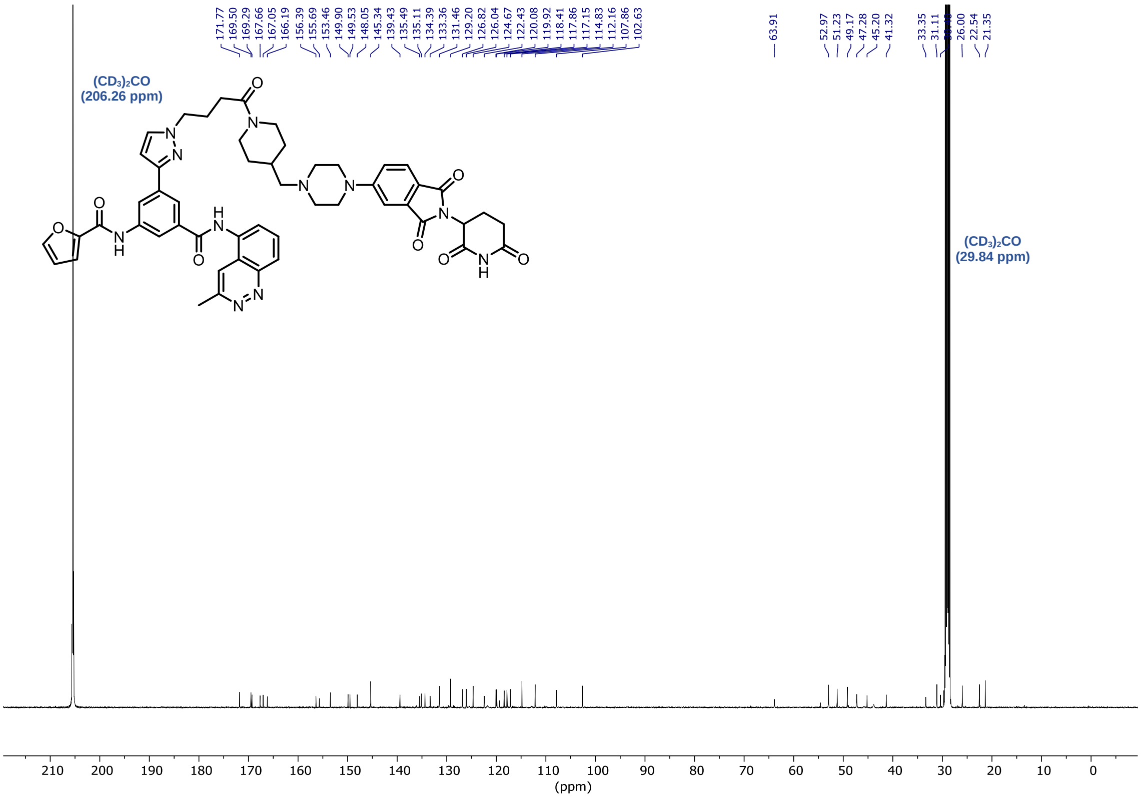

Compound S9. ¹HNMR (400 MHz, Acetone-*d*₆) and ¹³C NMR (126 MHz, Acetone-*d*₆).

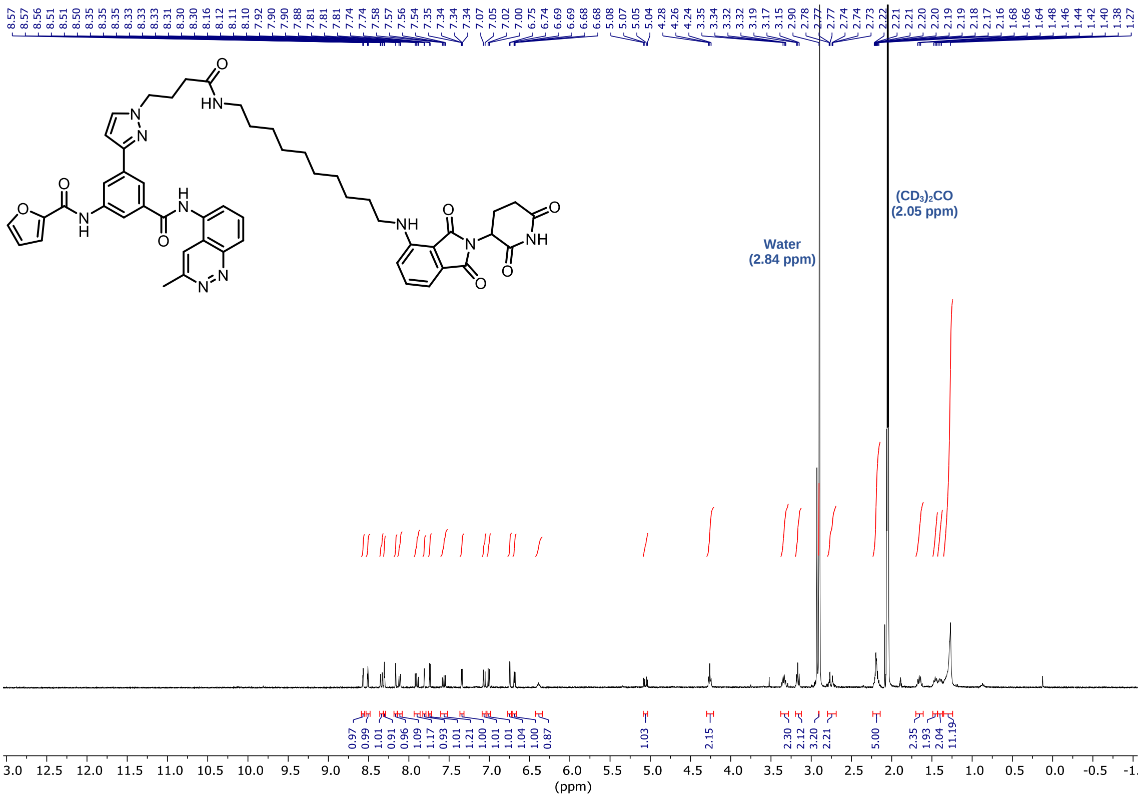

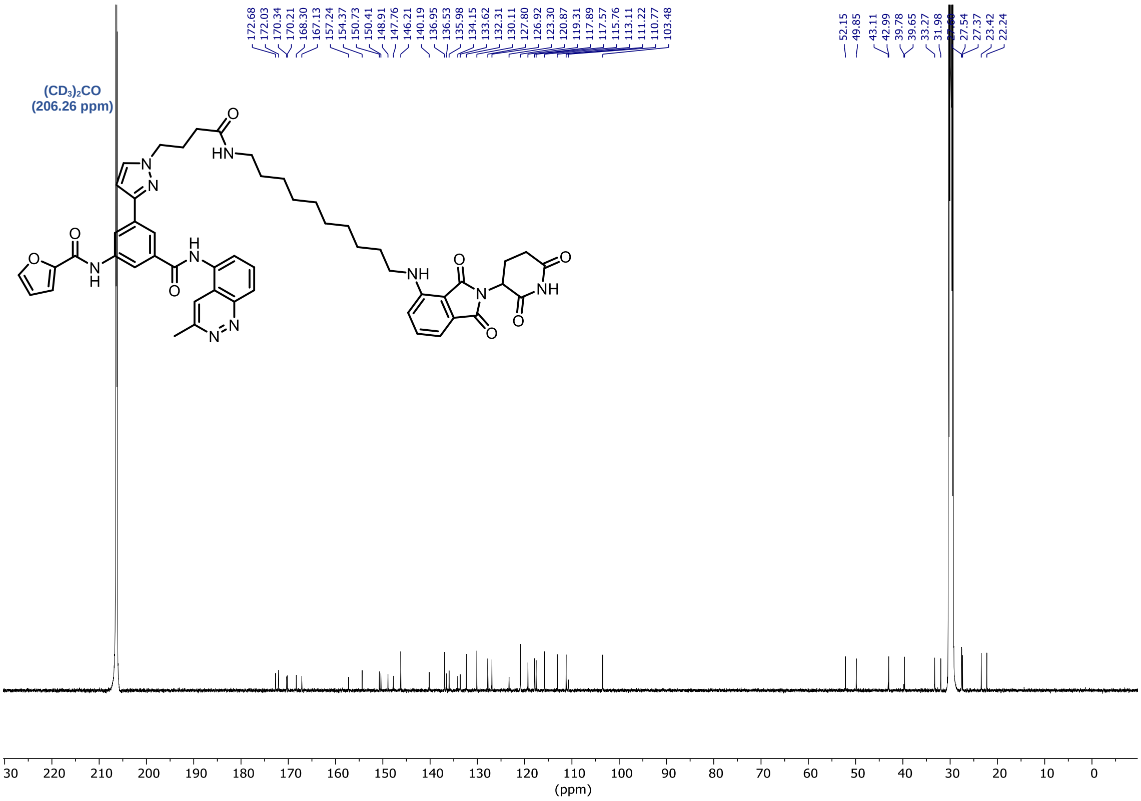

Compound dCE-2. ^{1}HNMR (400 MHz, DMSO-d_6) and ^{13}C NMR (126 MHz, DMSO-d_6).

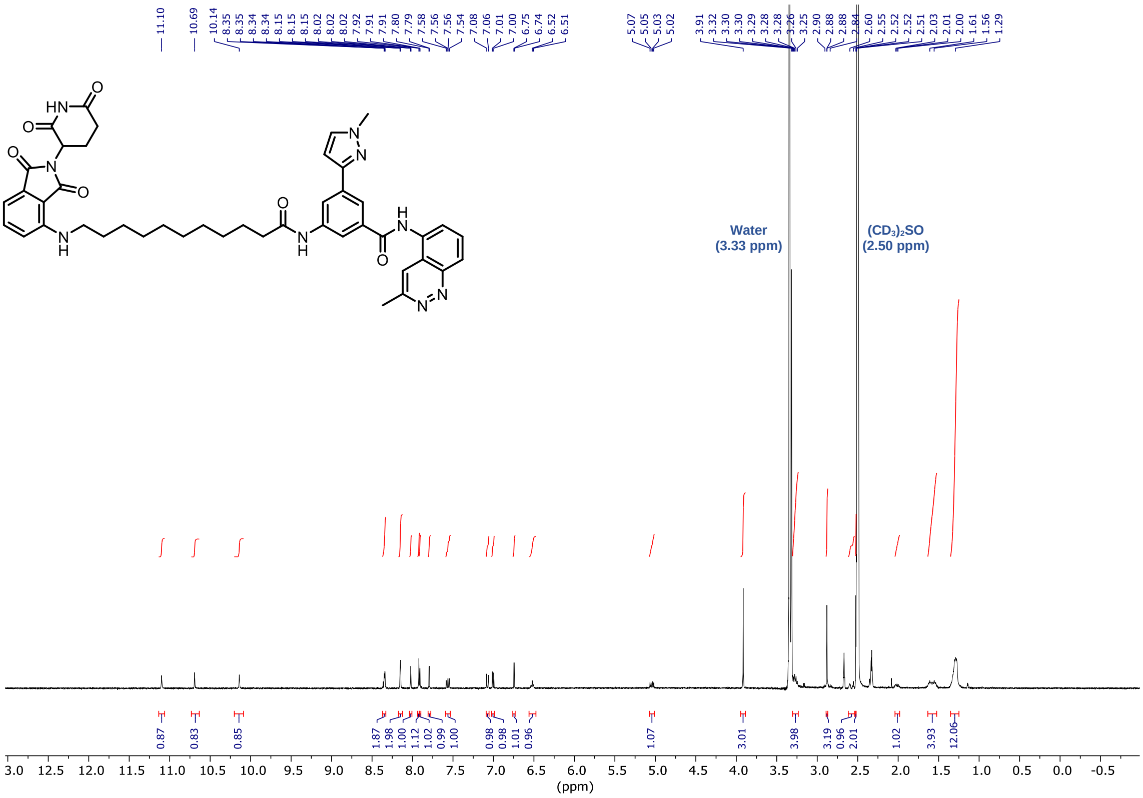

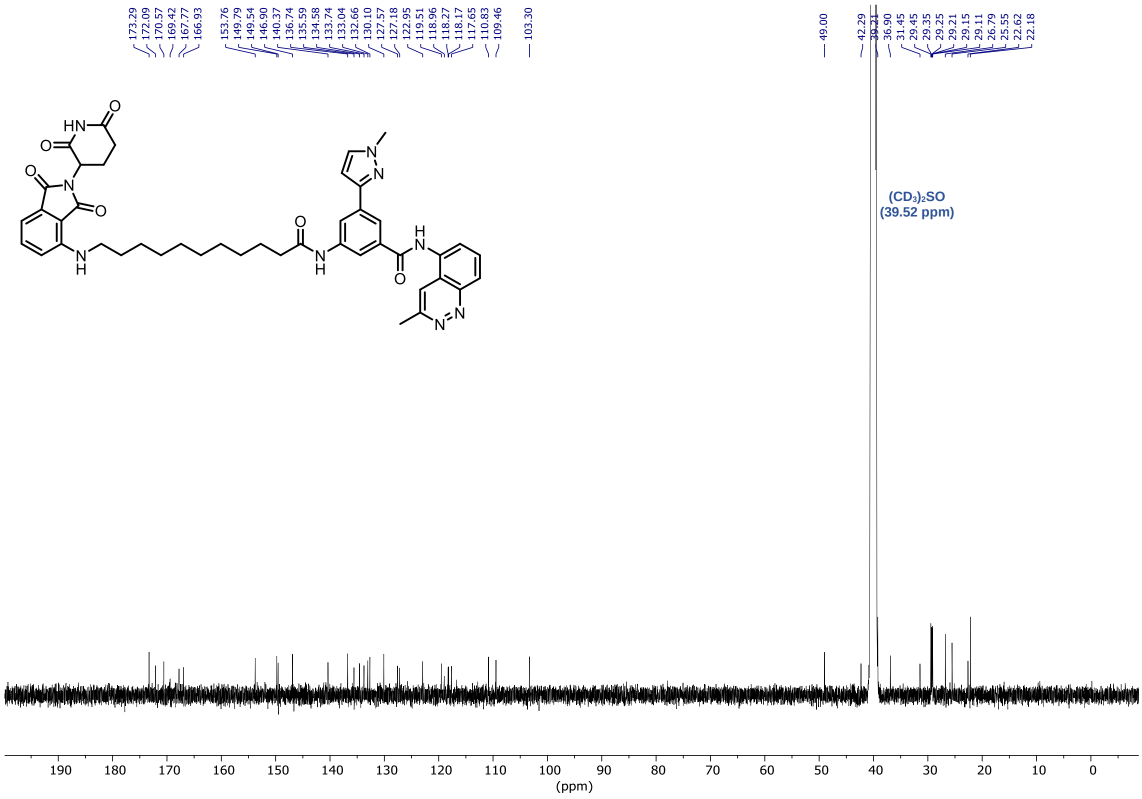

Compound 6. ^{1}H NMR (500 MHz, DMSO-d_6) and ^{13}C NMR (126 MHz, Methanol-d_4).

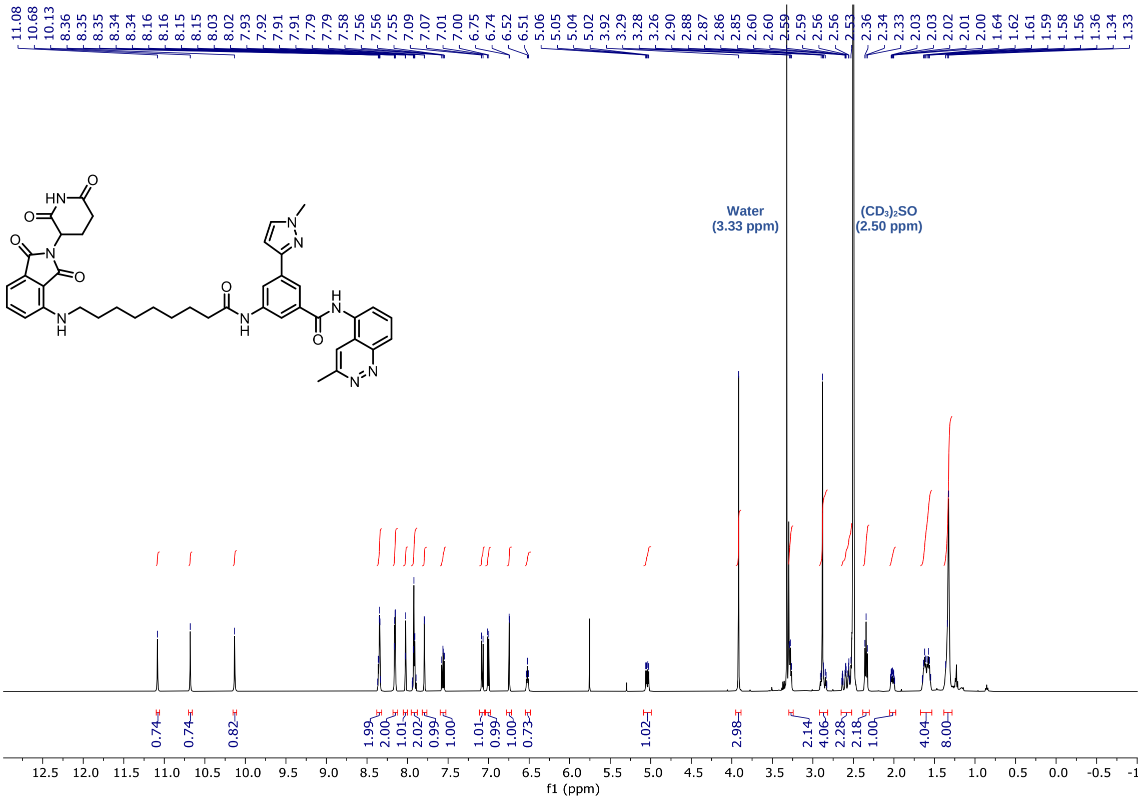

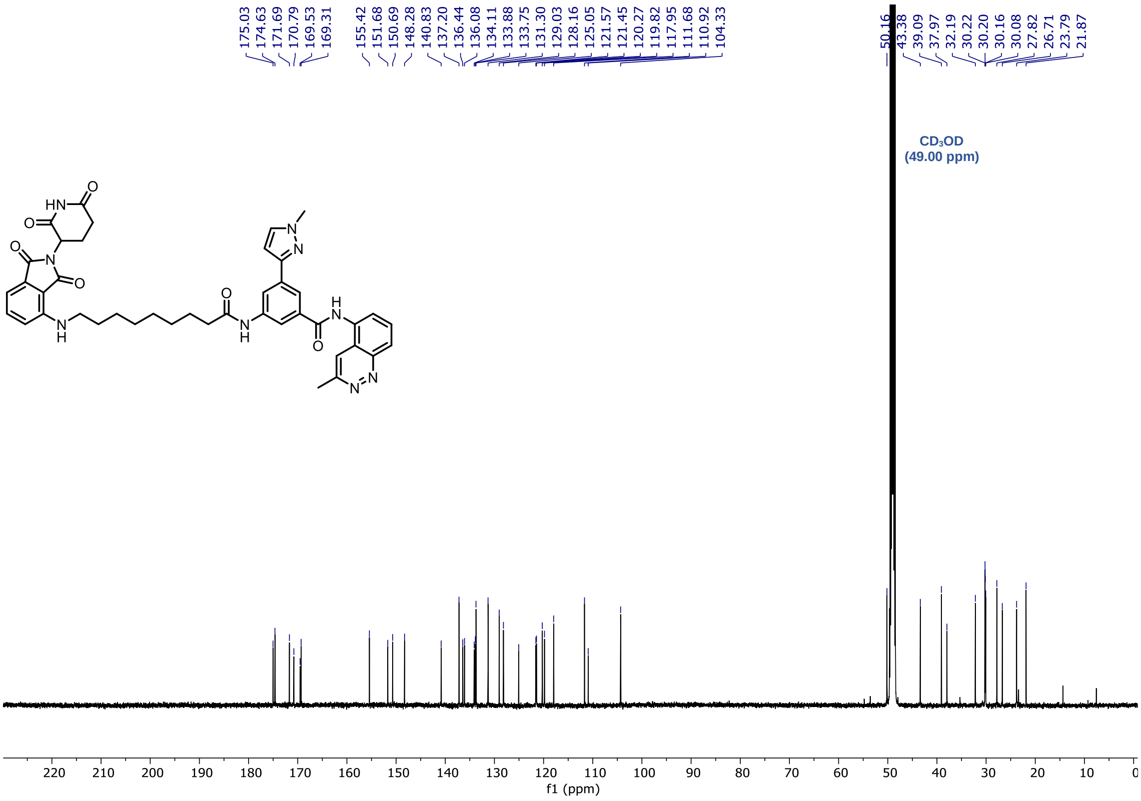

Compound 7 . ^{1}HNMR (500 MHz, DMSO-d_6) and ^{13}C NMR (126 MHz, DMSO-d_6).

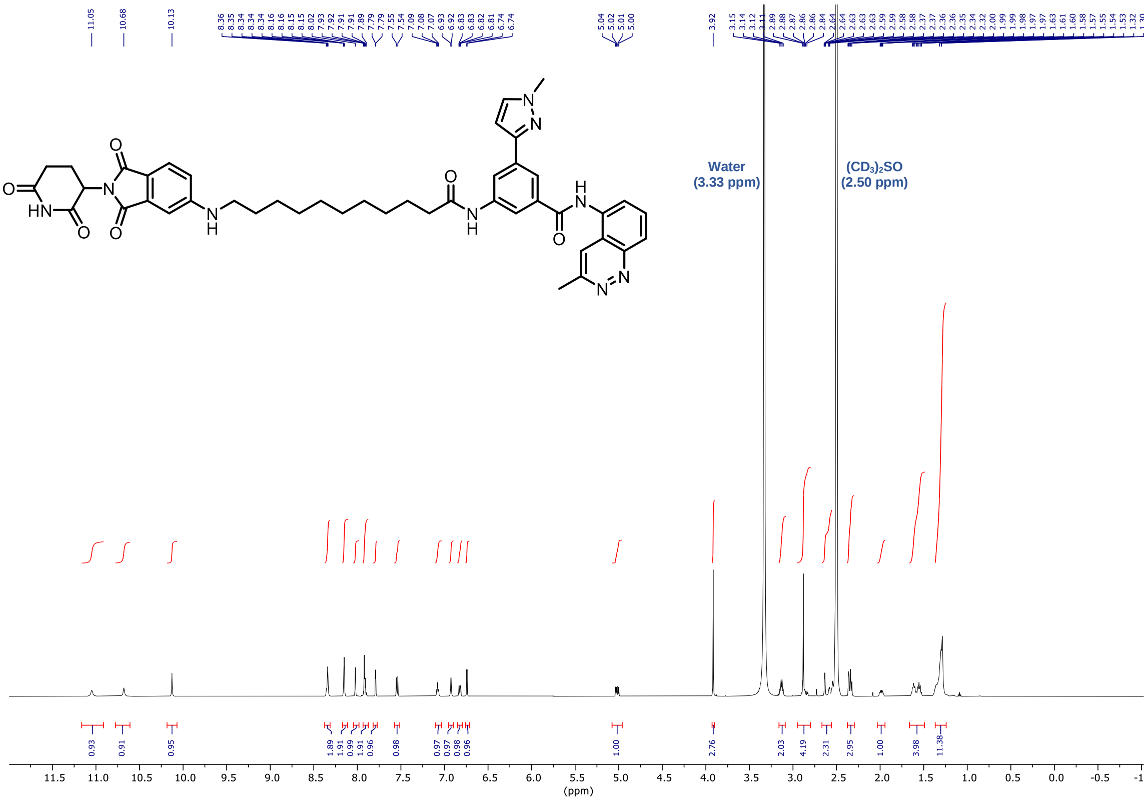

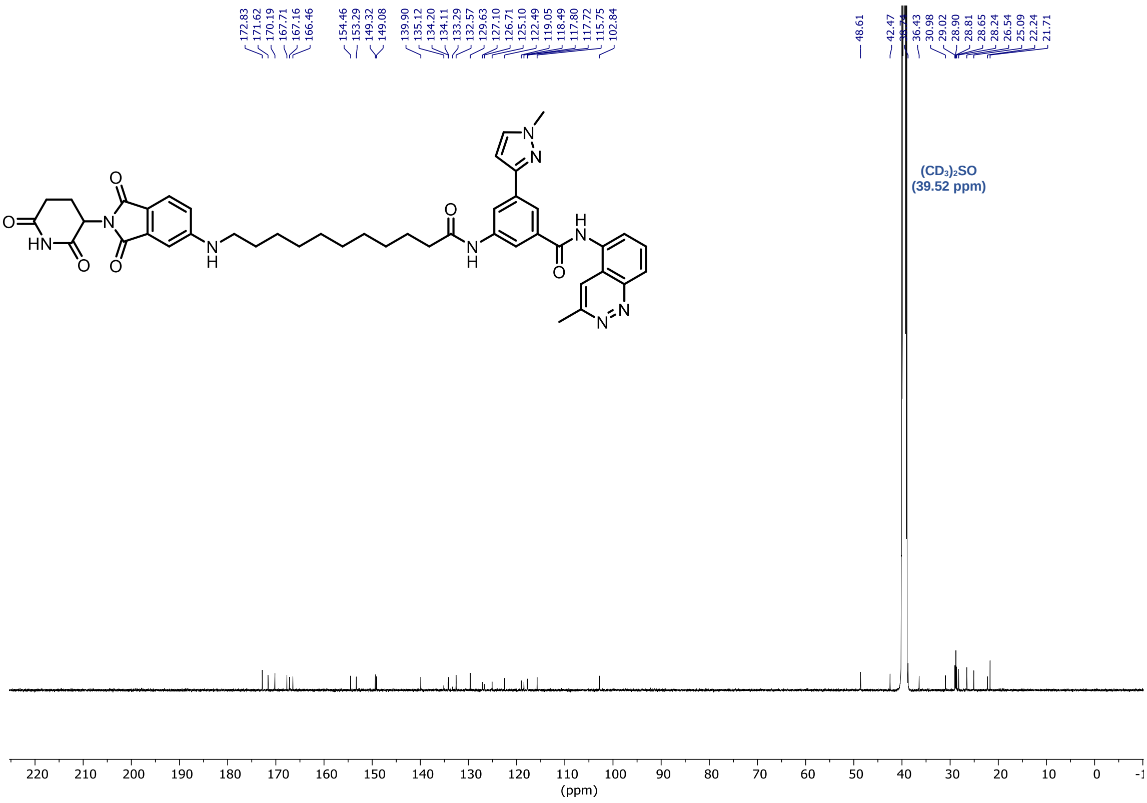

Compound 8. ^{1}HNMR (400 MHz, Acetone-d_6) and ^{13}C NMR (100 MHz, Acetone-d_6).

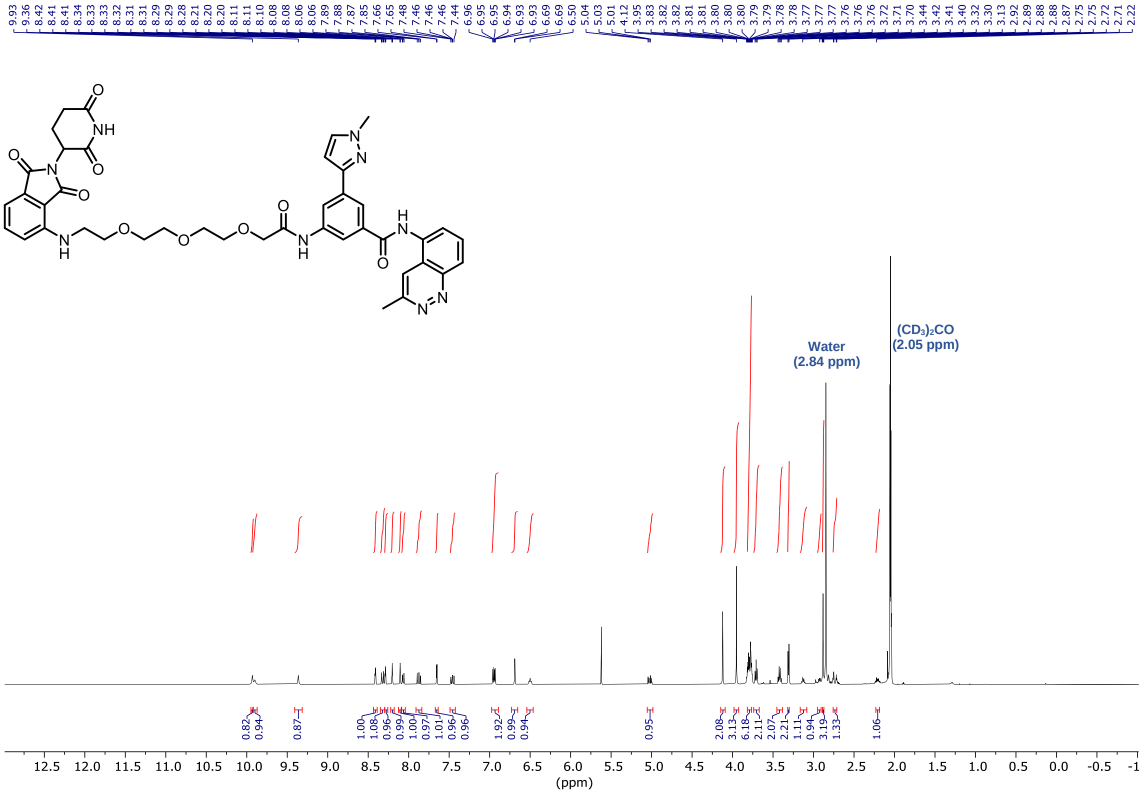

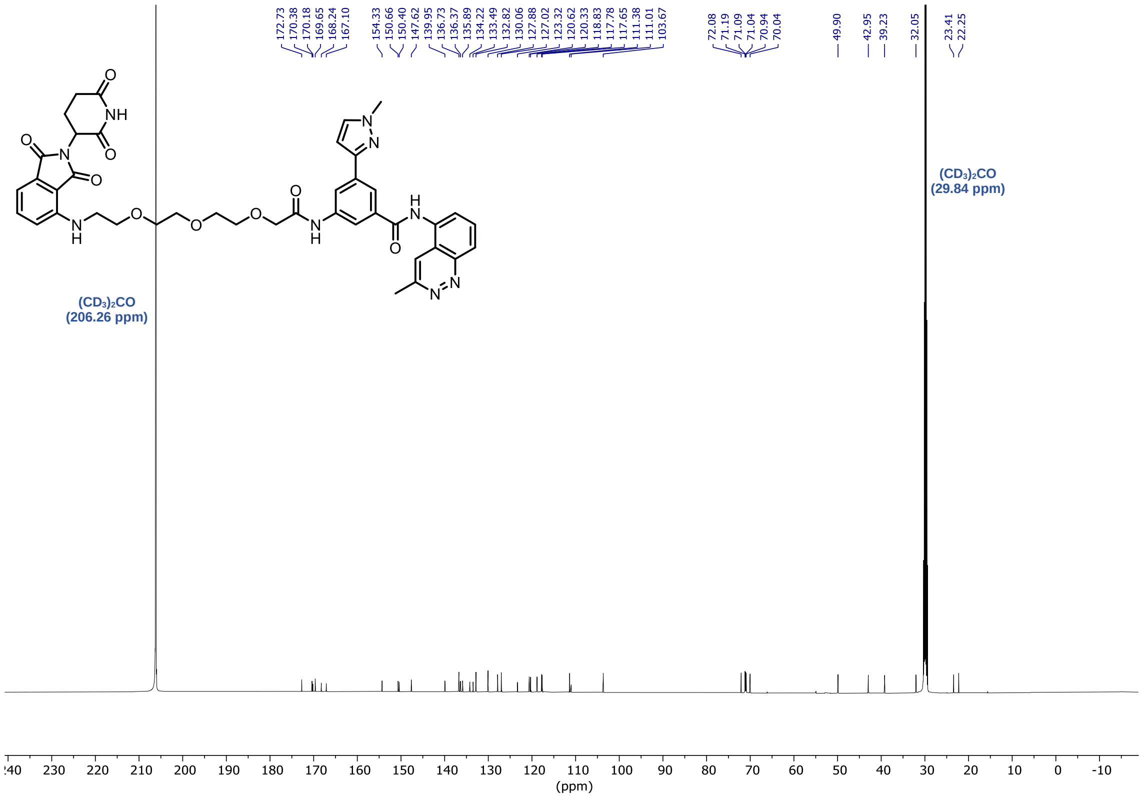

Compound S53. ^{1}H NMR (400 MHz, $CDCl_3$) and ^{13}C NMR (101 MHz, $CDCl_3$).

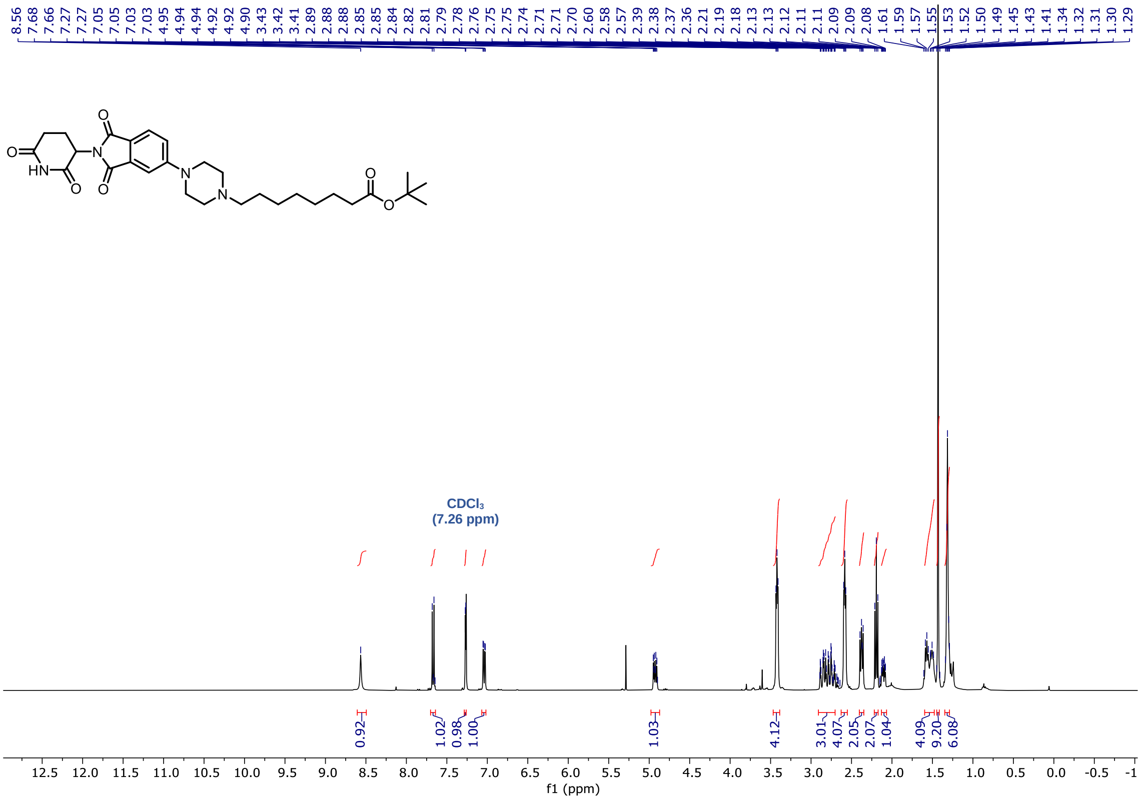

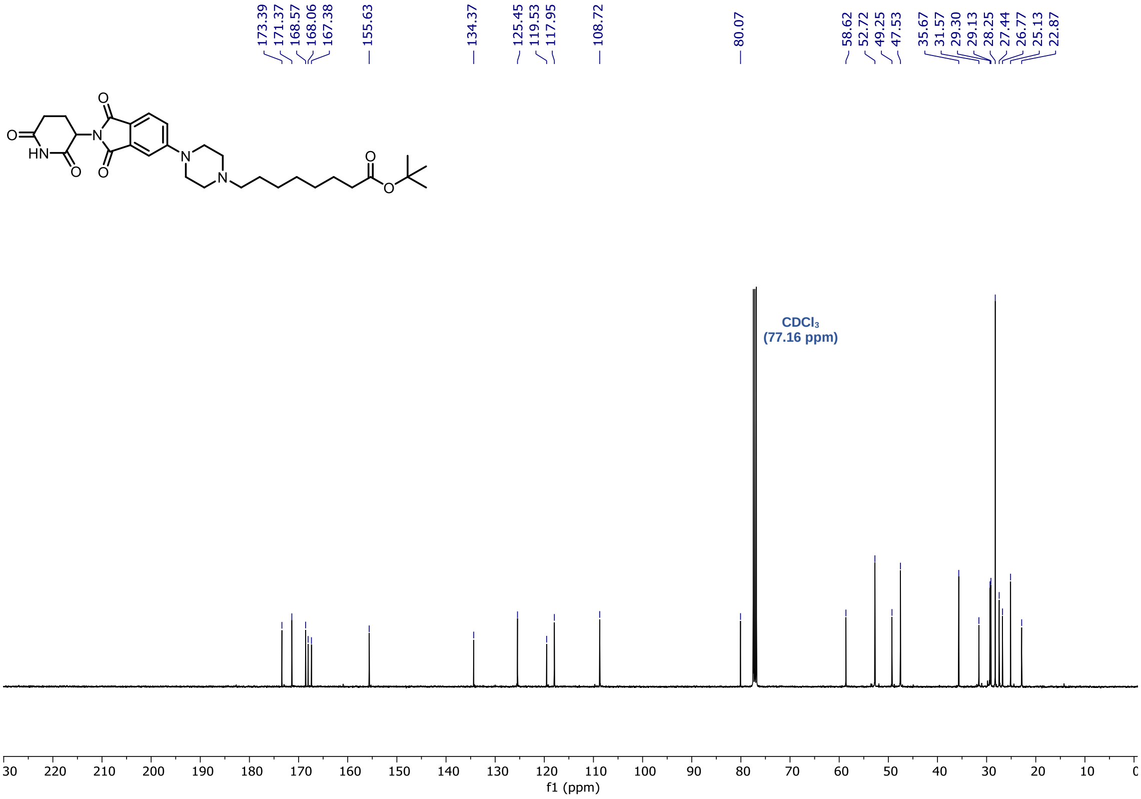

Compound 9. ^{1}H NMR (500 MHz, Acetone-d_6) and ^{13}C NMR (126 MHz, Acetone-d_6).

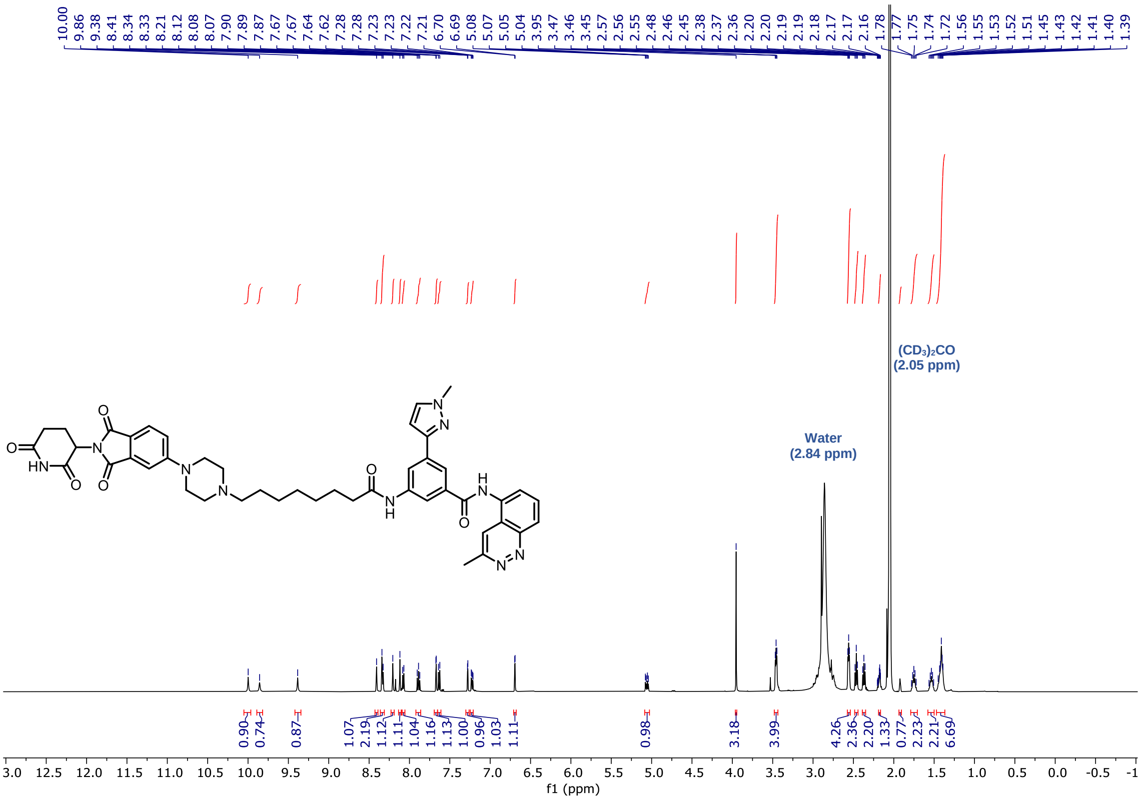

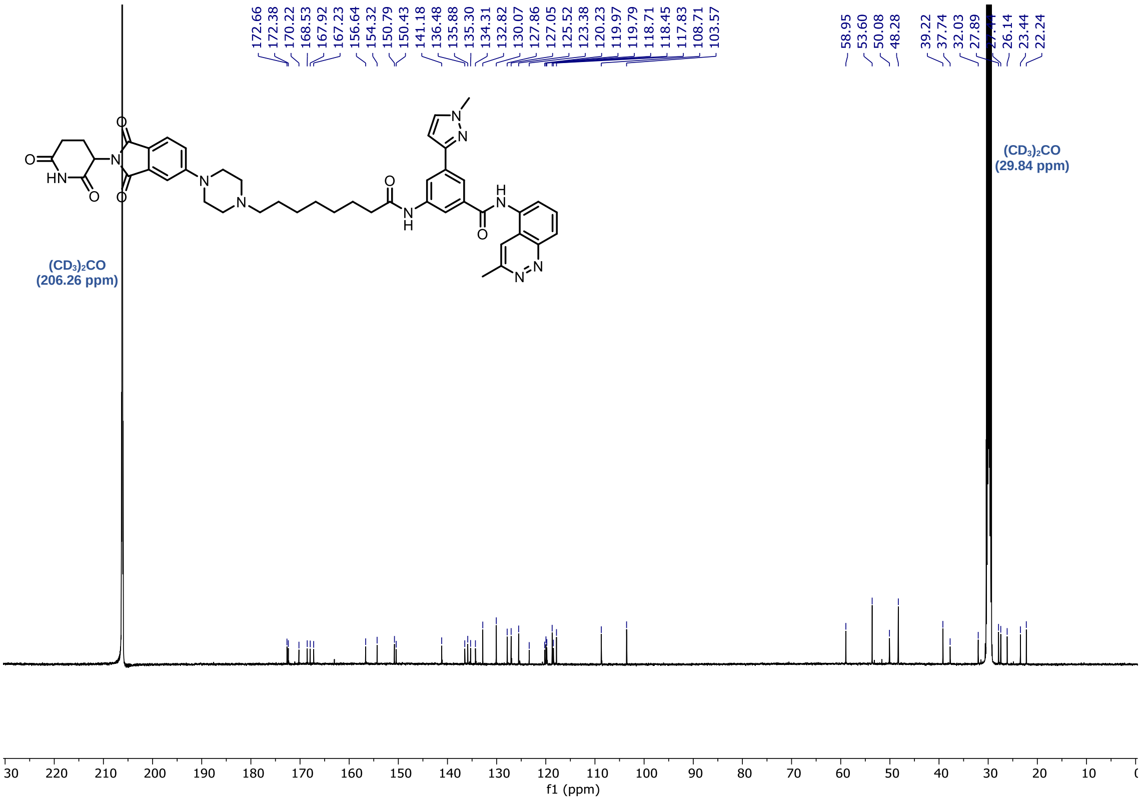

Compound S56. ^{1}HNMR (400 MHz, Methanol-d_4) and ^{13}C NMR (126 MHz, Methanol-d_4).

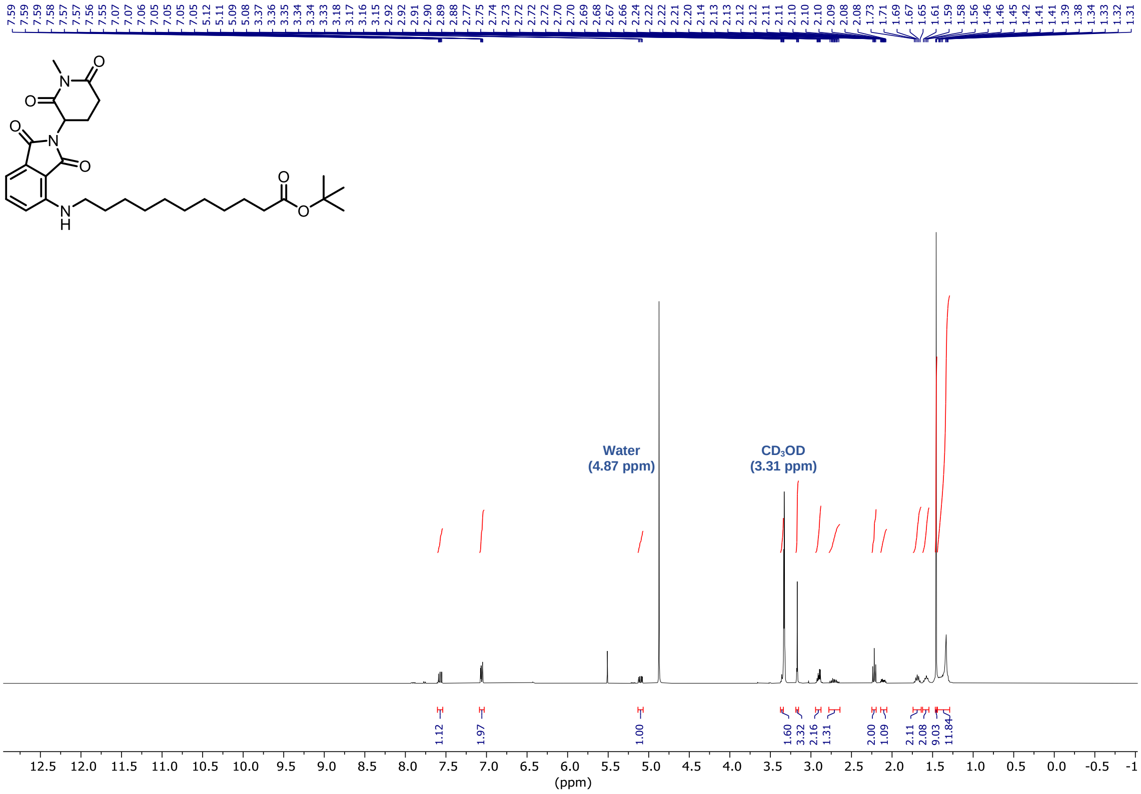

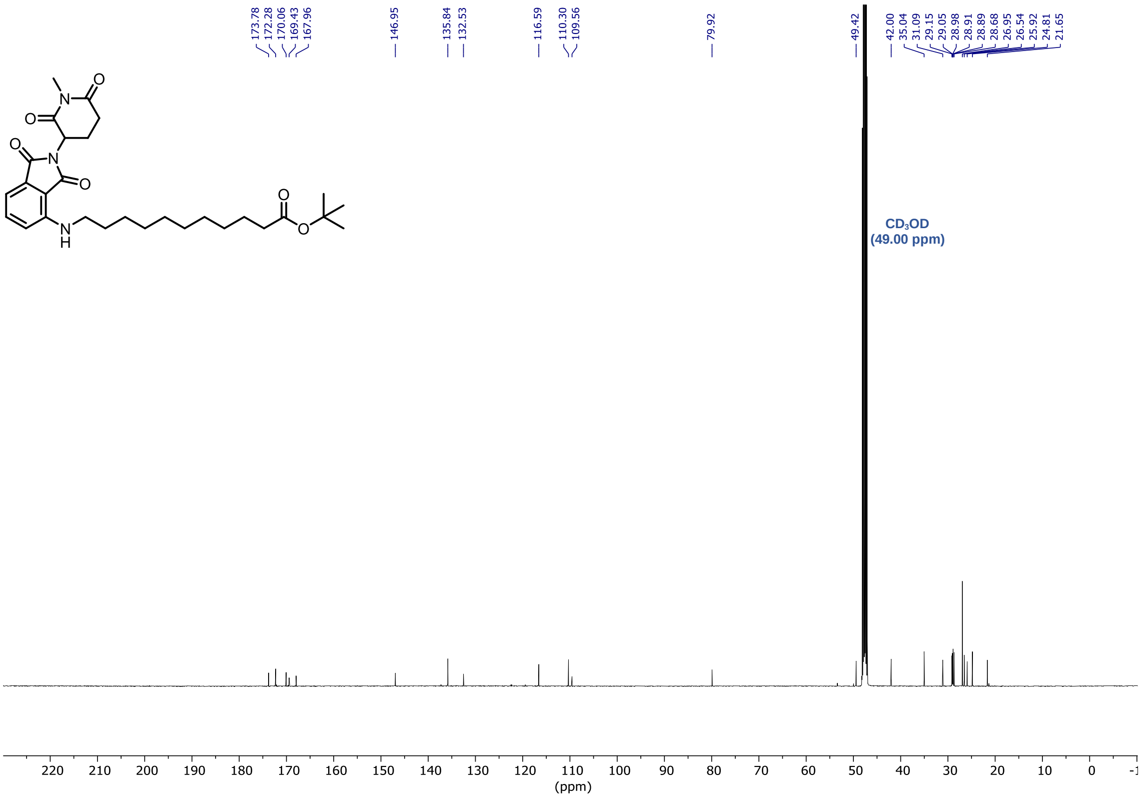

Compound 10. ^{1}HNMR (400 MHz, Acetone-d_6) and ^{13}C NMR (126 MHz, Acetone-d_6).

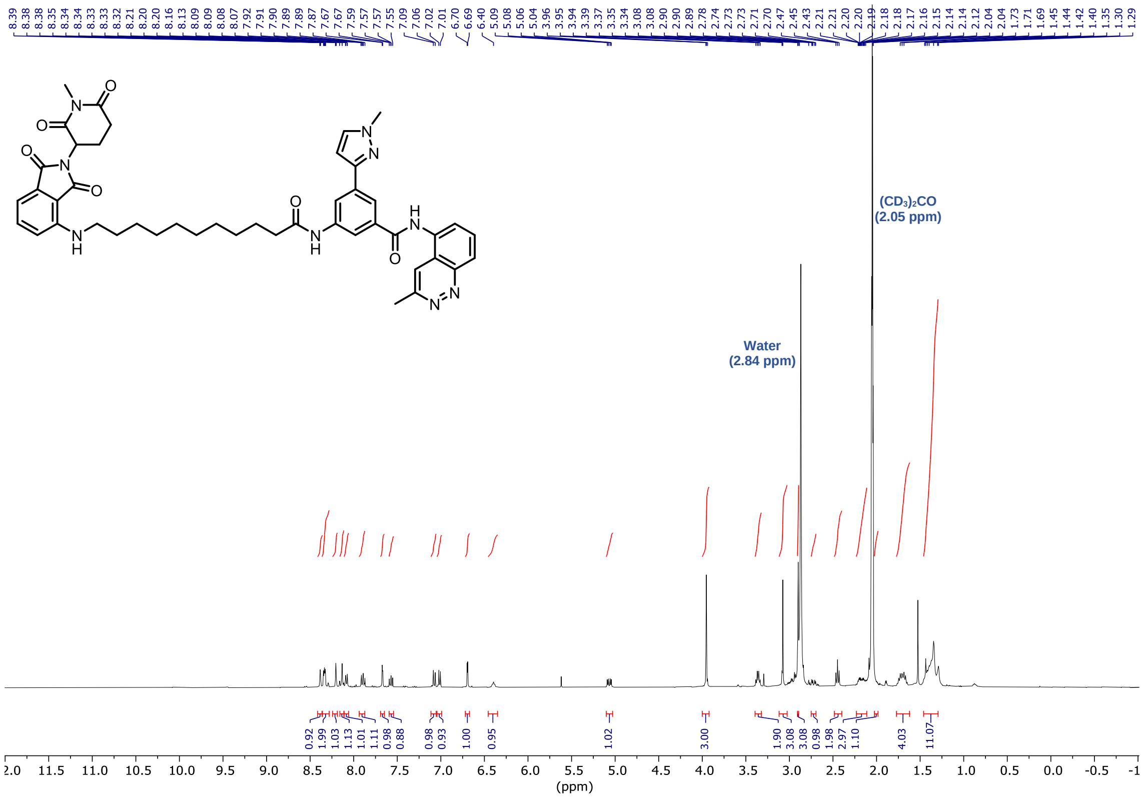

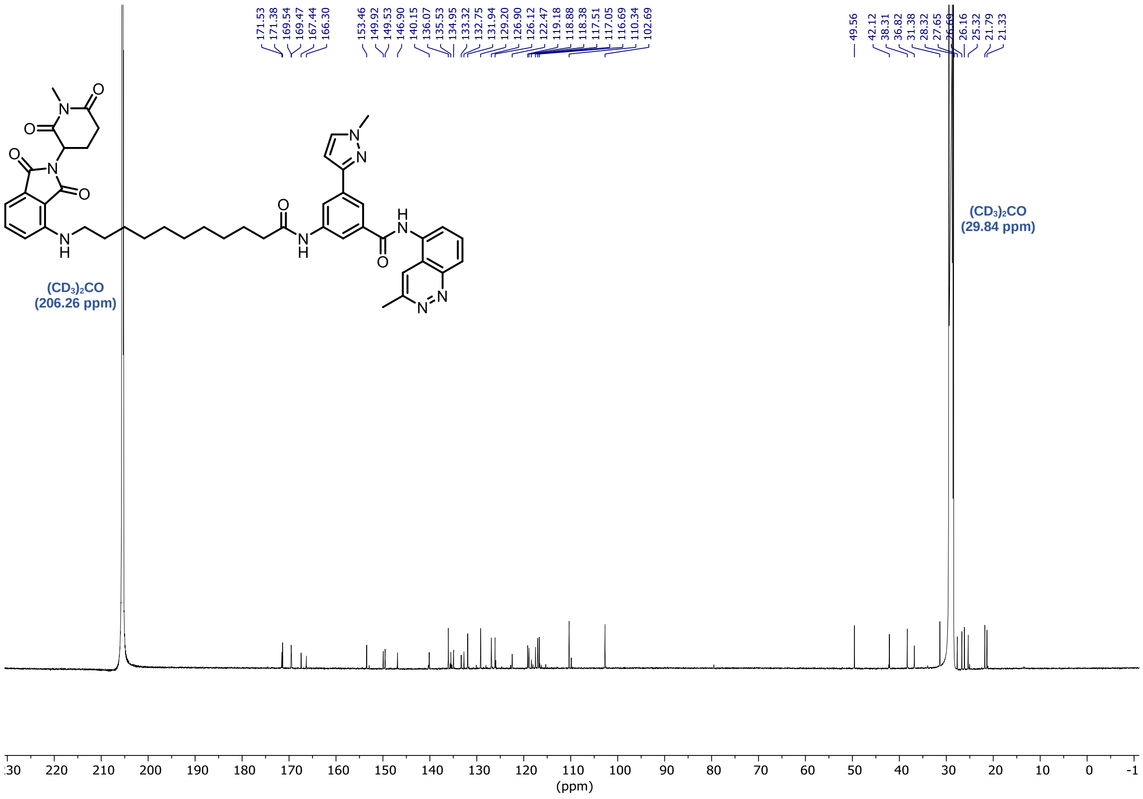

5. LCMS Data

Compound 3

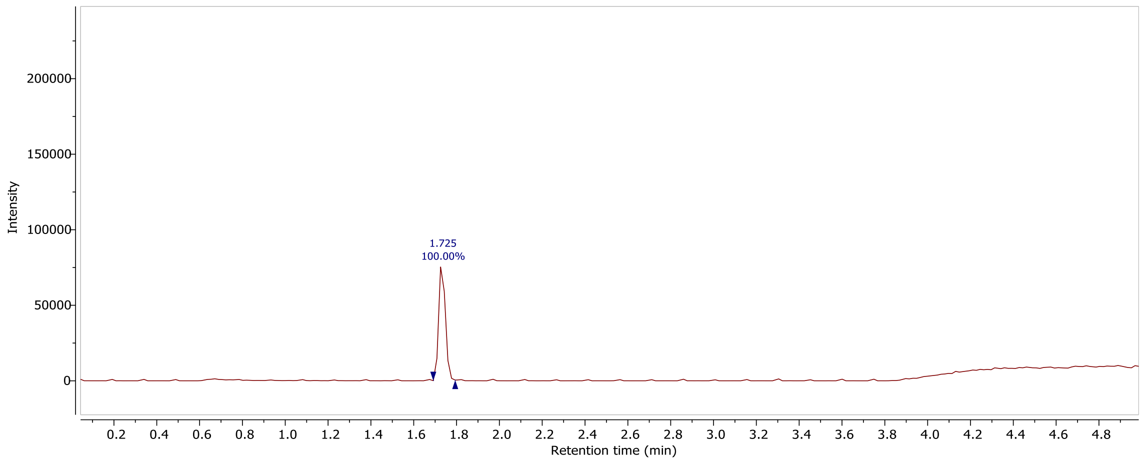

	RT	Area	Height	Total Height %	Total Area %	Start time	End time
1	1.725	163282.761	75230.533	100.00	100.00	1.691	1.794

Compound 4

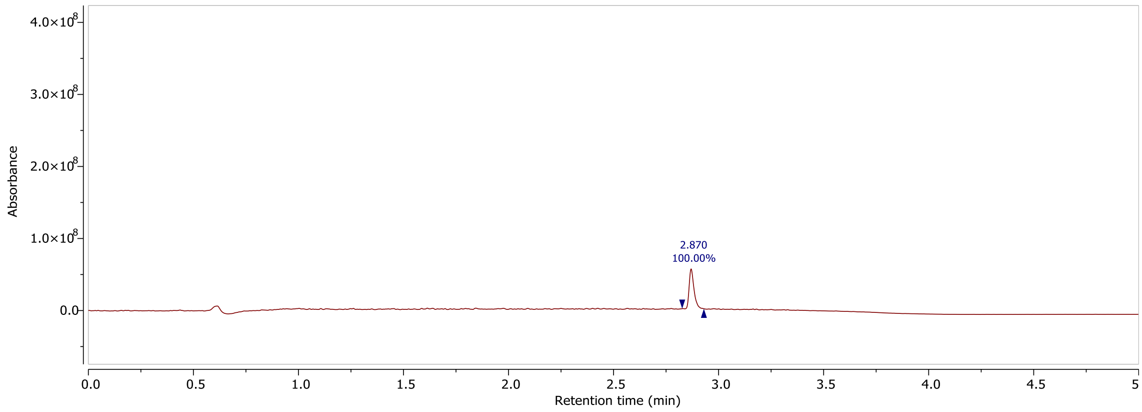

	RT	Area	Height	Total Height %	Total Area %	Start time	End time
1	2.870	881623149.403	55204983.387	100.00	100.00	2.827	2.930

Compound dCE-2

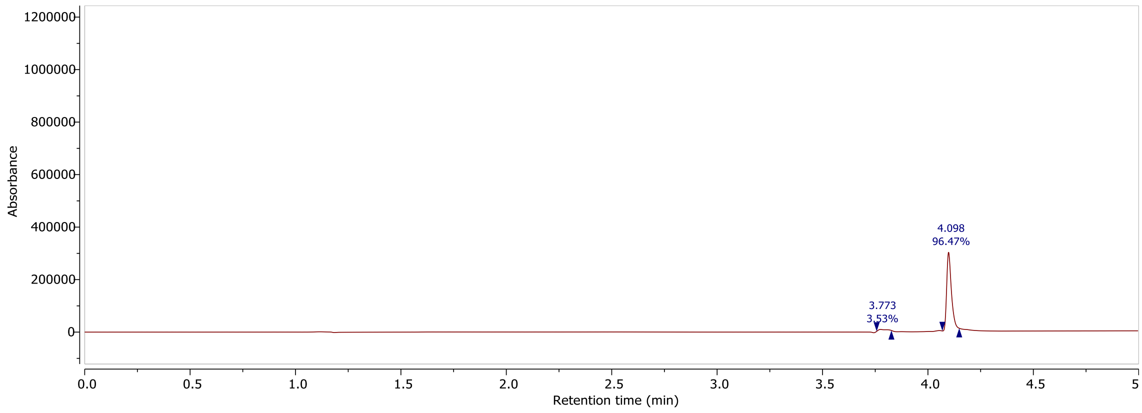

	RT	Area	Height	Total Height %	Total Area %	Start time	End time
1	3.773	166771.500	6520.095	2.16	3.53	3.757	3.827
2	4.098	4559891.219	296036.125	97.84	96.47	4.068	4.148

Compound 6

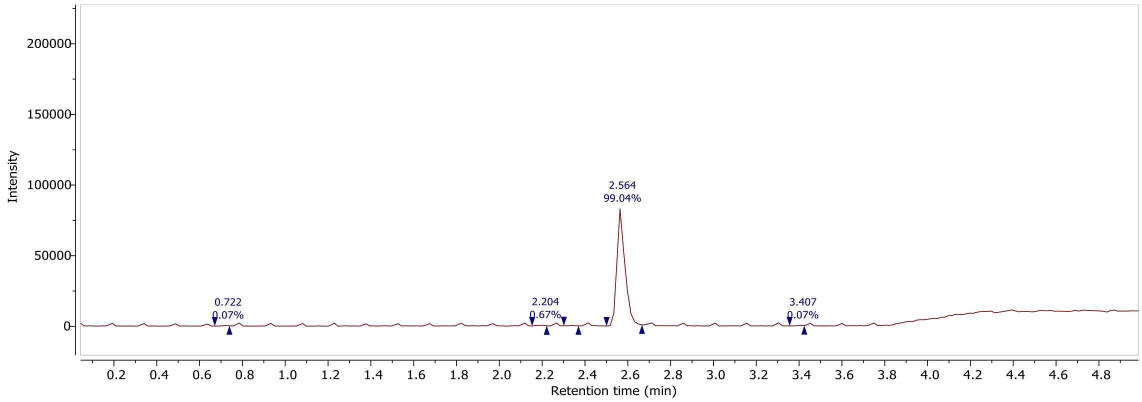

	RT	Area	Height	Total Height %	Total Area %	Start time	End time
1	0.722	134.888	123.335	0.15	0.07	0.671	0.739
2	2.204	1211.973	462.863	0.55	0.67	2.153	2.221
3	2.335	274.909	208.337	0.25	0.15	2.301	2.370
4	2.564	180267.024	82700.615	98.89	99.04	2.501	2.666
5	3.407	130.052	131.776	0.16	0.07	3.356	3.424

Compound 7

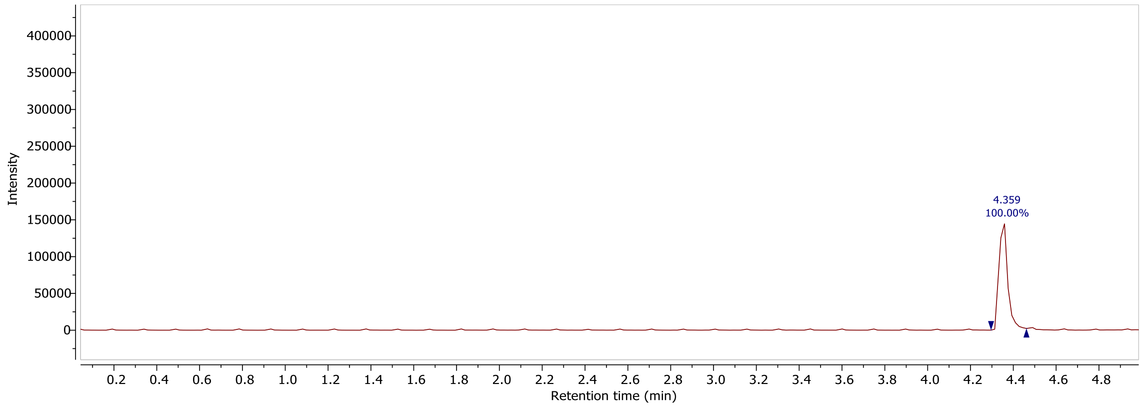

	RT	Area	Height	Total Height %	Total Area %	Start time	End time
1	4.359	357450.027	143943.969	100.00	100.00	4.296	4.461

Compound 8

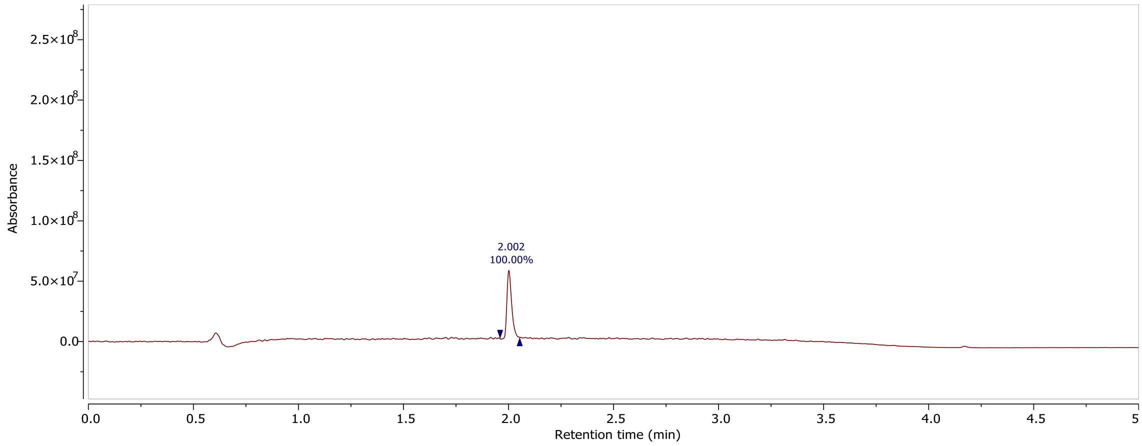

	RT	Area	Height	Total Height %	Total Area %	Start time	End time
1	2.002	868013712.929	56140102.821	100.00	100.00	1.960	2.053

Compound 9

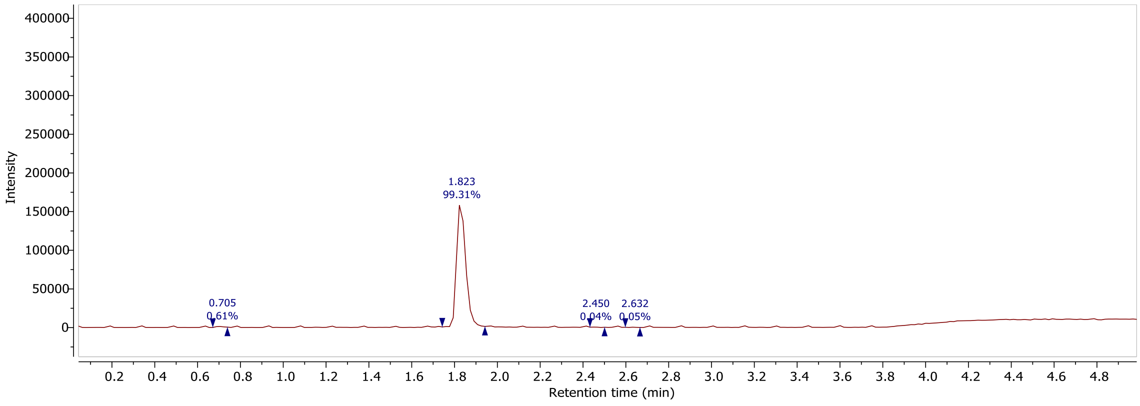

	RT	Area	Height	Total Height %	Total Area %	Start time	End time
1	0.705	2464.860	1044.632	0.66	0.61	0.671	0.739
2	1.823	402930.628	157131.768	99.15	99.31	1.743	1.942
3	2.450	143.079	114.466	0.07	0.04	2.432	2.501
4	2.632	194.634	193.227	0.12	0.05	2.598	2.666

Compound 10

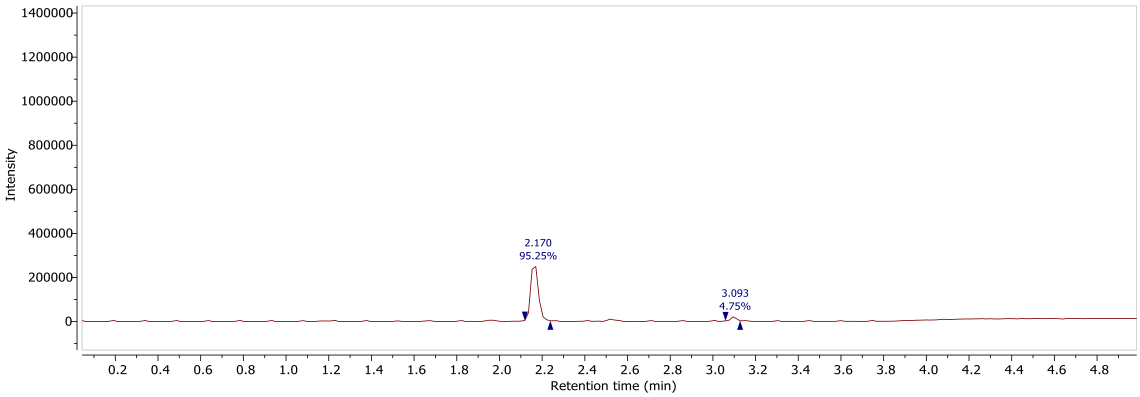

	RT	Area	Height	Total Height %	Total Area %	Start time	End time
1	2.170	628705.281	245975.594	93.10	95.25	2.119	2.238
2	3.093	31377.938	18225.683	6.90	4.75	3.059	3.128

6. References

(1) Vannam, R.; Sayilgan, J.; Ojeda, S.; Karakyriakou, B.; Hu, E.; Kreuzer, J.; Morris, R.; Lopez, X. I. H.; Rai, S.; Haas, W.; Lawrence, M.; Ott, C. J. Targeted Degradation of the Enhancer Lysine Acetyltransferases CBP and p300. *Cell Chem. Bio*. **2021**, *28*, 503–514.

(2) Chen, Z.; Wang, M.; Wu, D.; Bai, L.; Xu, T.; Metwally, H.; Wang, Y.; McEachern, D.; Zhao, L.; Li, R.; Takyi-Williams, J.; Wang, M.; Wang, L.; Li, Q.; Wen, B.; Sun, D.; Wang, S. Discovery of CBPD-268 as an Exceptionally Potent and Orally Efficacious CBP/p300 PROTAC Degrader Capable of Achieving Tumor Regression. *J. Med. Chem.* **2024**, *67*, 5275–5304.

(3) Luo, J.; Chen, Z.; Qiao, Y.; Ching-Yi J. T.; Young, E.; Mannan, R.; Mahapatra, S.; He, T.; Eyunni, S.; Zhang, Y.; Zheng, Y.; Su, F.; Cao, X.; Wang, R.; Cheng, Y.; Seri, R.; George, J.; Shahine, M.; Miner, S. J.; Vaishampayan, U.; Wang, M.; Wang, S.; Parolia, A.; Chinnaiyan, A. M. p300/CBP Degradation is Required to Disable the Active AR Enhanceosome in Prostate Cancer. *BioRxiv* **2024**. https://doi.org/10.1101/2024.03.29.587346

(4) Romero, F. A.; Murray, J.; Lai, K. W.; Tsui, V.; Albrecht, B. K.; An, L.; Beresini, M. H.; de Leon Boenig, G.; Bronner, S. M.; Chan, E. W.; Chen, K. X.; Chen, Z.; Choo, E. F.; Clagg, K.; Clark, K.; Crawford, T. D.; Cyr, P.; de Almeida Nagata, D.; Gascoigne, K. E.; Grogan, J. L.; Hatzivassiliou, G.; Huang, W.; Hunsaker, T. L.; Kaufman, S.; Koenig, S. G.; Li, R.; Li, Y.; Liang, X.; Liao, J.; Liu, W.; Ly, J.; Maher, J.; Masui, C.; Merchant, M.; Ran, Y.; Taylor, A. M.; Wai, J.; Wang, F.; Wei, X.; Yu, D.; Zhu, B.-Y.; Zhu, X.; Magnuson, S. GNE-781, A Highly Advanced Potent and Selective Bromodomain Inhibitor of Cyclic Adenosine Monophosphate Response Element Binding Protein, Binding Protein (CBP). *J. Med. Chem*. **2017**, *60*, 9162–9183.

(5) Gosselé K.; Latino I.; Laul E.; Kirillova M.; Pascanu V.; Carloni E.; Bedi, R. K.; Caflisch, A.; Gonzalez, S. F.; Nevado, C. Development of a Novel Class of CBP/EP300 Bromodomain Inhibitors which Block TNF-α Induced NFκB Signaling. *ChemRxiv.* **2024** doi:10.26434/chemrxiv-2024-tkd72.

(6) Wiedmer, L.; Eberle, S. A.; Kumar Bedi, R.; Śledź, P.; Caflisch, A. A Reader-Based Assay for m6A Writers and Erasers. *Anal. Chem*. **2019**, *91*, 3078–3084.

(7) Hughes, C. S.; Foehr S.; Garfield D. A.; Furlong E. E.; Steinmetz L. M.; Krijgsveld J. Ultrasensitive Proteome Analysis Using Paramagnetic Bead Technology. *Mol. Syst. Biol.* **2014**, *10*:757.

(8) Leutert M.; Rodriguez-Mias R. A.; Fukuda N. K.; Villén J. R2-P2 Rapid-Robotic Phosphoproteomics Enables Multidimensional Cell Signaling Studies. *Mol. Syst. Biol.* **2019**, *15*: e9021.

(9) Panse, C.; Trachsel, C.; Türker, C. Bridging Data Management Platforms and Visualization Tools to Enable Ad-Hoc and Smart Analytics in Life Sciences. *J. Integr. Bioinform.* **2022**; *19*: 20220031.

(10) Djomehri, S. I.; Gonzalez, M. E.; da Veiga Leprevost, F.; Tekula, S. R.; Chang, H.-Y.; White, M. J.; Cimino-Mathews, A.; Burman, B.; Basrur, V.; Argani, P.; Nesvizhskii, A. I.; Kleer, C. G. Quantitative Proteomic Landscape of Metaplastic Breast Carcinoma Pathological Subtypes and Their Relationship to Triple-Negative Tumors. *Nat. Commun.* **2020**, *11*, 1723.

(11) Wolski, W. E.; Nanni, P.; Grossmann, J.; d'Errico, M.; Schlapbach, R.; Panse, C. A Comprehensive *R*-Package for Proteomics Differential Expression Analysis. *J. Proteome. Res.* **2023**, *22*,1092–1104.

(12) Huber, W.; von Heydebreck, A.; Sueltmann, H.; Poustka, A.; Vingron, M.; Variance Stabilization Applied to Microarray Data Calibration and to the Quantification of Differential Expression. *Bioinformatics* **2022**, *18*, S96–S104.

(13) Smyth, G. K. Linear Models and Empirical Bayes Methods for Assessing Differential Expression in Microarray Experiments. *Stat. Appl. Genet. Mol*. **2004**, 3, 1.

(14) Benjamini, Y.; Hochberg, Y. Controlling the False Discovery Rate: A Practical and Powerful Approach to Multiple Testing. J. R. Stat. Soc., Ser. B (Methodological) **1995**, *57*, 289–300.

(15) Vanommeslaeghe, K.; Hatcher, E.; Acharya, C.; Kundu, S.; Zhong, S.; Shim, J.; Darian, E.; Guvench, O.; Lopes, P.; Vorobyov, I.; Mackerell Jr., A. D. CHARMM General Force Field: A Force Field for Drug-Like Molecules Compatible with The CHARMM All-Atom Additive Biological Force Fields. *J. Comput. Chem*. **2010**, *31*, 671–690.

(16) MacKerell, A. D.; Feig, M.; Brooks, C. L. Improved Treatment of the Protein Backbone in Empirical Force Fields *J. Am. Chem. Soc.* **2004**, *126*, 698–699.

(17) Scarsi, M.; Apostolakis, J.; Caflisch, A. Continuum Electrostatic Energies of Macromolecules in Aqueous Solutions. *J. Phys. Chem. A* **1997**, *101*, 8098–8106.

(18) Maestro 11.5, S., LLC, New York, NY, **2018**.

(19) MarvinSketch 21.20.0. ChemAxon, http://www.chemaxon.com.

(20) Price, D. J.; Brooks III, C. L. A Modified TIP3P Water Potential for Simulation with Ewald Summation. *J. Chem. Phys.* **2004**, *121*, 10096–10103.

(21) Brooks, B.R.; Brooks, C.L.; Mackerell, A.D.; Nilsson, L.; Petrella, R.J.; Roux, B.; Won, Y.; Archontis, G.; Bartels, C.; Boresch, S.; Caflisch, A.; Caves, L.; Cui, Q.; Dinner, A.R.; Feig, M.; Fischer, S.; Gao, J.; Hodoscek, M.; Im, W.; Kuczera, K.; Lazaridis, T.; Ma, J.; Ovchinnikov, V.; Paci, E.; Pastor, R.W.; Post, C.B.; Pu, J. Z.; Schaefer, M.; Tidor, B.; Venable, R.M.; Woodcock, H.L.; Wu, X.; Yang, W.; York, D. M.; Karplus, M. CHARMM: The Biomolecular Simulation Program. *J. Comput. Chem*. **2009**, *30*, 1545–1614.

(22) Essmann, U.; Perera, L.; Berkowitz, M.L.; Darden, T.; Lee, H.; Pedersen, L.G. A Smooth Particle Mesh Ewald Method. *J. Chem. Phys*. **1995**, *103*, 8577–8593.

(23) Phillips, J. C.; Braun, R.; Wang, W.; Gumbart, J.; Tajkhorshid, E.; Villa, E; Chipot, C.; Skeel, R. D.; Kale, L.; Schulten, K. Scalable Molecular Dynamics with NAMD. *J. Comput. Chem*. **2005**, *26*, 1781–1802.

(24) Martyna, G. J.; Tobias, D. J.; Klein, M. L. Constant Pressure Molecular Dynamics Algorithms. *J. Chem. Phys*. **1994**, *101*, 4177–4189.

(25) Feller, S. E.; Zhang, Y. H.; Pastor, R. W.; Brooks, B. R.; Constant-Pressure Molecular-Dynamics Simulation-the Langevin Piston Method. *J. Chem. Phys.* **1995**, *103*, 4613– 4621.

(26) OriginPro, Version 2021. OriginLab Corporation, Northampton, MA, USA. https://www.originlab.com/2021.

(27) Jun, Q.; Scott, A.; Lei, W. Compounds, Compositions, and Methods for Protein Degradation. WO 2020264172 A1, **2020**.

(28) Hong, J. Y.; Jing, H.; Price, I. R.; Cao, J.; Bai, J. J.; Lin. H. Simultaneous Inhibition of SIRT2 Deacetylase and Defatty-Acylase Activities via a PROTAC Strategy. ACS Med. Chem. Lett. 2020, 11, 2305–2311.

(29) Steinebach, C.; Sosič, I.; Lindner, S.; Bricelj, A.; Kohl, F.; Dora Ng, Y. L.; Monschke, M.; Wagner, K. G.; Krönke, J.; Gütschow. M. A MedChem Toolbox for Cereblon-Directed PROTACs. *MedChemComm.* **2019**, *10*, 1037–1041.

(30) Yu, Z.; Liu, Q.; Li, Q.; Huang, Z.; Yang, Y.; You, J. Remote Editing of Stacked Aromatic Assemblies for Heteroannular C−H Functionalization by a Palladium Switch between Aromatic Rings. *Angew. Chem. Int. Ed*. **2022**, *61*, e202212079.

(31) Hu, X.; Nguyen, K. T.; Jiang, V. C.; Lofland, D.; Moser, H. E.; Pei, D. Macrocyclic Inhibitors for Peptide Deformylase: A Structure−Activity Relationship Study of the Ring Size. *J. Med. Chem.* **2004**, *47*, 4941–4949.

(32) Xiao, Z.; Song, S.; Chen, D.; van Merkerk, R.; van der Wouden, P. E.; Cool, R. H.; Quax, W. J.; Poelarends, G. J.; Melgert, B. N.; Dekker, F. J. Proteolysis Targeting Chimera (PROTAC) for Macrophage Migration Inhibitory Factor (MIF) Has Anti-Proliferative Activity in Lung Cancer Cells. *Angew. Chem. Int. Ed.* **2021**, *60*, 17514–17521.

(33) Sparks, S. M.; Araujo, E.; Berlin, M.; Zhang, W.; Wang, J. Selective Modulators of Mutant Lrrk2 Proteolysis and Associated Methods of Use. WO 2021194878 A1, **2021**.

(34) Konstantinidou, M.; Oun, A.; Pathak, P.; Zhang, B.; Wang, Z.; Brake, F.; Dolga, A. M.; Kortholt, A.; Dömling, A. The tale of proteolysis targeting chimeras (PROTACs) for Leucine-Rich Repeat Kinase 2 (LRRK2). *ChemMedChem* **2021**,*16*, 959–965.

(35) Qi, Z.; Yang, G.; Deng, T.; Wang, J.; Zhou, H.; Popov, S. A.; Shults, E. E.; Wang, C. Design and Linkage Optimization of Ursane-Thalidomide-based PROTACs and Identification of their Targeted-Degradation Properties to MDM2 Protein. *Bioorg. Chem*. **2021**, *111*, 104901.

(36) Brownsey, D. K.; Rowley, B. C.; Gorobets, E.; Gelfand, B. S.; Derksen, D. J. Rapid Synthesis of Pomalidomide-Conjugates for the Development of Protein Degrader Libraries. *Chem. Sci.* **2021**, *12*, 4519–4525.

(37) Yang, X.; Wang, Z.; Pei, Y.; Song, N.; Xu, L.; Feng, B.; Wang, H.; Luo, X.; Hu, X.; Qiu, X.; Feng, H.; Yang, Y.; Zhou, Y.; Li, J.; Zhou, B. Discovery of Thalidomide-based PROTAC Small Molecules as the Highly Efficient SHP2 Degraders. *Eur. J. Med. Chem*. **2021**, *218*, 113341.

(38) Yeon, H. J.; Sunhong, K.; Du, H. J.; Yun, C. S.; Pilho, K.; Soo, Y. C.; Jin, K. H.; Goo, P. S.; Chul, P. B.; Hoon, K. J. Target Protein Eed Degradation-inducing Degraducer, Preparation Method Thereof, and Pharmaceutical Composition for Preventing or Treating Diseases Related to Eed, Ezh2, or Prc2, Comprising Same as Active Ingredient. WO 2020162725 A1, **2020**.

(39) Hwang, J. Y.; Ha, J. D.; Cho, S.Y.; Kim, P.; Park, B. C.; Kim, S.; Kim, J. H.; Park, S. G. Cereblon Protein Degradation Inducing Compound, Preparation Method Therefor and Pharmaceutical Composition for Preventing or Treating Cancer, Containing Same as Active Ingredient. WO 2019078522 A1, **2019**.

(40) Mainolfi, N.; Ji, N.; Kluge, A. F.; Weiss, M. M.; Zhang, Y.; Zheng, X. Irak Degraders and Uses Thereof. WO 2020113233 A1, **2020**.

(41) Liu, X.; Kalogeropulou, A. F.; Domingos, S.; Makukhin, N.; Nirujogi, R. S.; Singh, F.; Shpiro, N.; Saalfrank, A.; Sammler, E.; Ganley, I. G.; Moreira, R.; Alessi, D. R.; Ciulli, A. Discovery of XL01126: A Potent, Fast, Cooperative, Selective, Orally Bioavailable, and Blood–Brain Barrier Penetrant PROTAC Degrader of Leucine-Rich Repeat Kinase 2. *J. Am. Chem. Soc*. **2022**, *144*, 16930–16952.

(42) Li, Q.; Guo, Q.; Wang, S.; Wan, S.; Li, Z.; Zhang, J.; Wu, X. Design and Synthesis of Proteolysis Targeting Chimeras (PROTACs) as an EGFR Degrader based on CO-1686. *Eur. J. Med. Chem*. **2022**, *238*, 114455.
